# Novel HLA class I associations with HIV-1 control in a unique genetically admixed population

**DOI:** 10.1038/s41598-018-23849-7

**Published:** 2018-04-17

**Authors:** Humberto Valenzuela-Ponce, Selma Alva-Hernández, Daniela Garrido-Rodríguez, Maribel Soto-Nava, Thalía García-Téllez, Tania Escamilla-Gómez, Claudia García-Morales, Verónica Sonia Quiroz-Morales, Daniela Tapia-Trejo, Silvia del Arenal-Sánchez, Francisco-Javier Prado-Galbarro, Ramón Hernández-Juan, Edna Rodríguez-Aguirre, Akio Murakami-Ogasawara, Carlos Mejía-Villatoro, Ingrid Y. Escobar-Urias, Rodolfo Pinzón-Meza, Juan Miguel Pascale, Yamitzel Zaldivar, Guillermo Porras-Cortés, Carlos Quant-Durán, Ivette Lorenzana, Rita I. Meza, Elsa Y. Palou, Marvin Manzanero, Rolando A. Cedillos, Carmen Aláez, Mark A. Brockman, P. Richard Harrigan, Chanson J. Brumme, Zabrina L. Brumme, Santiago Ávila-Ríos, Gustavo Reyes-Terán, The Mesoamerican HIV Project Group, Karla A. Romero-Mora, María Gómez-Palacio, Sandra Pinto-Cardoso, Sabrina Navas, Leticia García, Cristina Quintana, Yaxelis Mendoza, Sumaya Moreira, Bismarck Hernández, Wendy Murillo, Candy Carbajal, Leda Parham, Diana Valladares, Luisa Pineda, Dixiana Flores, Roxana Motiño, Víctor Umanzor, Oneyda Méndez, Nadina Romero, Jonahi Lizama, María L. Méndez, David de los Santos Cebrero, César Rivera-Benitez, Juan Sierra-Madero, Audelia Alanis-Vega, Luz A. González-Hernández, Jaime Andrade-Villanueva, Jaime Álvarez-Zayas, Héctor Carrillo-Martínez, José L. Centeno, Everardo Barreto, Tanya Campos, Jesús Oaxaca-Navarro, Ricardo Aya de la Fuente, César A. Carrasco-Ayala, Lesvia M. Rivera-Abarca, Gabriela Velázquez, Elizabeth Papaqui-Limón, Indiana Torres-Escobar, María J. del Carmen-Ricalde, David Valenzo-Loaeza, Carlos A. Barrera-Arellano, Adrián Flores-Gaxiola, Carlos A. Avilez-Gaxiola, Adonay Jiménez-Jiménez, Juan Beltrán-Saldaña, Arturo Artega-Martínez, Elizabeth Domínguez-Ramírez, Jorge M. de la Roca-Chiapas, Miriam J. García-Collins, Hilda Basilio-Badillo, Dulce M. Cruz-Lavadores, Carlos R. González-Álvarez, Luis E. Arias-Tlaculio, Samuel Navarro-Álvarez

**Affiliations:** 1 National Institute of Respiratory Diseases, CIENI Center for Research in Infectious Diseases, Mexico City, Mexico; 2 Hospital Roosevelt, Infectious Diseases Clinic, Guatemala City, Guatemala; 3 Instituto Conmemorativo Gorgas de Estudios de la Salud, Panama City, Panama; 4 Hospital Vivian Pellas, Managua, Nicaragua; 5 Hospital Roberto Calderón, Managua, Nicaragua; 6 Universidad Nacional Autónoma de Honduras, Tegucigalpa, Honduras; 7 Honduras HIV National Laboratory, Tegucigalpa, Honduras; 8 Hospital Escuela Universitario, Tegucigalpa, Honduras; 9 Ministry of Health, Belmopan, Belize; 10 Hospital Nacional Rosales, San Salvador, El Salvador; 11 National Institute of Genomic Medicine, Translational Medicine Laboratory, Mexico City, Mexico; 12 Simon Fraser University, Faculty of Health Sciences, Burnaby, Canada; 13 British Columbia Centre for Excellence in HIV/AIDS, Vancouver, Canada; 14 Present Address: Institut Pasteur, Unité HIV, Inflammation and Persistence, Paris, France; 15 Mario Catarino Rivas Hospital, San Pedro Sula, Honduras; 16 Atlantida Hospital, La Ceiba, Honduras; 17 Hospital del Sur, Choluteca, Honduras; 18 CAPASITS Acapulco, Guerrero, Mexico; 19 General Hospital, Mexico City, Mexico; 20 National Institute of Medical Sciences and Nutrition, Mexico City, Mexico; 21 Civil Hospital Fray Antonio Alcalde, Guadalajara, Mexico; 22 CAPASITS Puerto Vallarta, Jalisco, Mexico; 23 CAPASITS Nezahualcóyotl, State of Mexico, Mexico; 24 CAPASITS Tlalnepantla, State of Mexico, Mexico; 25 CAPASITS Cuernavaca, Morelos, Mexico; 26 CAPASITS Monterrey, Nuevo Leon, Mexico; 27 SEAI Oaxaca, Oaxaca, Mexico; 28 CAPASITS Puebla, Puebla, Mexico; 29 CAPASITS Cancún, Quintana Roo, Mexico; 30 CAPASITS Culiacán, Sinaloa, Mexico; 31 CAPASITS Hermosillo, Sonora, Mexico; 32 Dr. Juan Graham Casasus Hospital, Tabasco, Mexico; 33 CAPASITS Tampico, Tamaulipas, Mexico; 34 General Hospital, Veracruz, Mexico; 35 Subregional Hospital (Coatzacoalcos), Veracruz, Mexico; 36 Subregional Hospital (Río Blanco), Veracruz, Mexico; 37 Subregional Hospital (Poza Rica), Veracruz, Mexico; 38 CAPASITS Mérida, Yucatan, Mexico; 39 CAPASITS Valladolid, Yucatan, Mexico; 40 General Hospital Tijuana, Baja California, Mexico

## Abstract

Associations between HLA class I alleles and HIV progression in populations exhibiting Amerindian and Caucasian genetic admixture remain understudied. Using univariable and multivariable analyses we evaluated HLA associations with five HIV clinical parameters in 3,213 HIV clade B-infected, ART-naïve individuals from Mexico and Central America (MEX/CAM cohort). A Canadian cohort (HOMER, n = 1622) was used for comparison. As expected, HLA allele frequencies in MEX/CAM and HOMER differed markedly. In MEX/CAM, 13 *HLA*-*A*, 24 *HLA*-*B*, and 14 *HLA*-*C* alleles were significantly associated with at least one clinical parameter. These included previously described protective (*e*.*g*. B*27:*05*, B*57:*01*/*02*/*03* and B*58:*01*) and risk (*e*.*g*. B*35:*02*) alleles, as well as novel ones (*e*.*g*. A*03:*01*, B*15:*39* and B*39:*02* identified as protective, and A*68:*03*/*05*, B*15:*30*, B*35:*12*/*14*, B*39:*01*/*06*, B*39:05~C*07:*02*, and B*40:01~C*03:*04* identified as risk). Interestingly, both protective (e.g. B*39:*02*) and risk (e.g. B*39:*01*/*05*/*06*) subtypes were identified within the common and genetically diverse *HLA*-B*39 allele group, characteristic to Amerindian populations. While HLA-HIV associations identified in MEX and CAM separately were similar overall (Spearman’s rho = 0.33, p = 0.03), region-specific associations were also noted. The identification of both canonical and novel HLA/HIV associations provides a first step towards improved understanding of HIV immune control among unique and understudied Mestizo populations.

## Introduction

Polymorphism within the human leukocyte antigen (HLA) class I (*HLA*-*A*, -*B* and -*C*) loci represents the strongest host genetic modifier of HIV disease progression^1–4^. However, while HLA associations with HIV disease outcome have been extensively studied in Caucasian and African populations^1,3,5–26^, Mestizo and other populations exhibiting complex genetic admixture remain understudied in this context. Populations in Mesoamerica (defined here as Mexico and Central America) possess unique immunogenetics as a result of admixture between mainly Amerindian and Caucasian, as well as African HLA haplotypes^27,28^, making these ideal for the identification of novel HLA correlates of HIV control. Here, we investigate HLA associations with five HIV clinical parameters among HIV-1 subtype B-infected, antiretroviral naive individuals in Mexico (MEX cohort, n = 1679) and 6 Central American countries: Guatemala, Belize, Honduras, El Salvador, Nicaragua and Panama (CAM cohort, n = 1534). We begin by characterizing the unique immunogenetics of this Mestizo population by comparing HLA allele frequencies (AF) and haplotype structures (linkage disequilibrium, LD) to those of a mainly Caucasian cohort from British Columbia, Canada (HOMER, n = 1622)^29,30^. We then define protective and risk HLA class I alleles in the individual (MEX, CAM) and combined Mesoamerican (MEX/CAM) cohorts using a novel approach that scores HLA alleles based on their associations with five interlinked clinical parameters relevant to HIV disease progression, while adjusting for HLA linkage disequilibrium, co-expression of known protective HLA alleles and other potential confounding factors. Finally, we explore the extent to which HLA protective/risk alleles observed in the MEX and CAM cohorts are universal versus region-specific.

## Methods

### Ethics statement

Recruitment and study of the Mexican and Central American cohorts was evaluated by the Ethics Committee of the National Institute of Respiratory Diseases (INER) in Mexico City (protocol codes E02–05, E10–10, E06–09). All experiments were performed in accordance to the protocol guidelines and regulations of our Institution, and approved by the Ethics Committee of INER. All participants were adults (over 18 years) and gave written informed consent in accordance with the Declaration of Helsinki before blood sample donation. Analysis of the BC HOMER reference cohort was approved and conducted according to the protocol guidelines and regulations of the Providence Health Care/University of British Columbia Research Ethics Board. All participants gave written informed consent and/or data were anonymized by REB-approved procedures.

### Mexican and Central American cohorts

Three thousand two hundred and thirteen HIV-1 clade B-infected, Antiretroviral Treatment (ART)-naïve individuals from Mexico and from 6 out of 7 Central American countries were enrolled by convenience sampling from 2000 to 2016 as part of an international multicenter cross-sectional study to evaluate HIV molecular epidemiology, drug resistance prevalence and HLA adaptation in Mesoamerica. Individuals were enrolled and donated a single blood sample at the time of HIV diagnosis or at follow-up visits prior to starting ART according to national guidelines. Every HIV-infected person naive to ART attending each participating clinic was offered the opportunity to participate during active recruitment periods. No additional exclusion criteria were applied. In Mexico (MEX cohort), 1679 participants were enrolled in a national collaborative network of clinics from 23 out of 32 Mexican states comprising Baja California, Campeche, Chiapas, Chihuahua, Colima, Guerrero, Hidalgo, Jalisco, Mexico City, Michoacan, Morelos, Nuevo Leon, Oaxaca, Puebla, Queretaro, Quintana Roo, Sinaloa, Sonora, State of Mexico, Tabasco, Tlaxcala, Veracruz and Yucatan. From Central America (CAM cohort), 1534 subjects were recruited, including 418 from Guatemala, 102 from Belize, 42 from El Salvador, 402 from Honduras, 254 from Nicaragua and 316 from Panama, using convenience sampling. Participating institutions in Central America included: Guatemala: Roosevelt Hospital, Guatemala City (a national referral center)^31^; Belize: Ministry of Health, Belmopan; El Salvador: Rosales National Hospital, San Salvador; Honduras: University School Hospital, Tegucigalpa; National Cardio-Pulmonary Institute, Tegucigalpa; Mario Catarino Rivas Hospital, San Pedro Sula; Atlántida Hospital, La Ceiba; South Hospital, Choluteca (five of the largest HIV clinics across the country)^32^; Nicaragua: Roberto Calderón Hospital, Managua (the largest reference center in the country)^33^; Panama: Gorgas Memorial Institute for Health Studies, Panama City (a national reference center)^34^. Demographic data were obtained via questionnaire at the time of sample donation. Blood samples, completed consent forms and demographic questionnaires were shipped to the Centre for Research in Infectious Diseases (CIENI) of the National Institute of Respiratory Diseases (INER) in Mexico City, a WHO-accredited laboratory for HIV genotyping, within 72 hours of collection. HIV clinical information (baseline drug resistance test, plasma viral load [pVL] and CD4 counts) were sent back to the different Mexican states or countries in Central America for clinical follow-up of the participants.

### HOMER cohort

The HAART Observational Medical Evaluation and Research (HOMER) cohort^35^ from British Columbia, Canada, a historic retrospective observational cohort comprising HIV-infected antiretroviral naive individuals initiating their first combination antiretroviral treatment regimen since 1996, was used as a reference for HLA allele frequency and HLA-pVL associations comparison. Clinical measurements from the earliest available time point before initiating ART were used. pVL in the HOMER cohort were performed with either Roche COBAS AmpliPrep/COBAS AMPLICOR HIV-1 MONITOR UltraSensitive Test, version 1.5 (1996–2008) or Roche COBAS AmpliPrep/COBAS TaqMan v1 HIV-1 Test (2008–2010). Median pVL and CD4 counts in the HOMER cohort were 4.90 (IQR 4.33–5.26) log_10_ copies/mL and 340 (IQR 170–500) cells/µL respectively. The cohort was predominantly male (86.2%), and the median age at recruitment was 37.4 (IQR 32–44) years. HOMER is primarily composed of Caucasian individuals (>60%); a minority of individuals self-identified as Hispanic (~2%) or Indigenous/Amerindian (~13%)^35^. The present analysis was restricted to subtype B-infected persons (n = 1622; 90% of total cohort^35^). As described elsewhere^29,30^, the majority of HLA class I types were defined at subtype level resolution, with the remaining intermediate-resolution data imputed to subtype-level resolution using a machine learning algorithm trained on *HLA*-*A*, *B* and *C* subtypes from >13,000 individuals with known ethnicity^36^.

### HIV subtyping

HIV subtypes were determined using REGA HIV Subtyping Tool (3.0) (http://dbpartners.stanford.edu:8080/RegaSubtyping/stanford-hiv/typingtool/) and confirmed with the Recombination Identification Program^37^ (RIP, www.hiv.lanl.gov/content/sequence/RIP/RIP.html) using available plasma HIV *pol* (protease and reverse transcriptase) sequences, obtained as described elsewhere^31^. All non-subtype B-infected individuals were removed prior to analysis.

### HIV clinical parameters

HIV pVL was determined by automated real-time polymerase chain reaction (PCR) using the m2000 system (Abbott, Abbott Park, IL, USA) with a detection limit of 40 HIV RNA copies/mL. CD45^+^, CD3^+^, CD4^+^ and CD8^+^ cell counts were obtained by flow cytometry using the Trucount Kit in FACSCanto II instrument (BD Biosciences, San Jose, CA).

### HLA class I typing

Peripheral blood mononuclear cells (PBMCs) were isolated by density gradient centrifugation (Ficoll-Paque Pharmacia, Uppsala, SE) from blood samples from MEX cohort, while buffy coats were isolated from CAM cohort blood samples and cryopreserved until DNA extraction. Total genomic DNA was extracted from PBMCs (∼6 million cells) or buffy coats (200 µL) using the QIAmp Blood Mini Kit (QIAGEN, Valencia, CA), according to the manufacturer’s specifications. *HLA*-*A*, -*B* and -*C* types were resolved at subtype-level (e.g. second field/4-digit) resolution using a previously described protocol with some modifications^38^. Briefly, a nested polymerase chain reaction (PCR) using universal, locus-specific primers was used to amplify a ∼1000 base pair region spanning exons 2 and 3 (which encode the α1 and α2 domains of the HLA peptide binding groove) of *HLA*-*A*, -*B* and -*C* loci, using the Expand High Fidelity PCR system (Roche Applied Science, Laval, PQ) (3.5 U/µL). For *HLA*-*A* PCR reactions, 5% dimethyl sulfoxide (DMSO, Sigma-Aldrich, St Louis, MO) was added to decrease unspecific amplification and primer dimerization. *HLA*-*B* PCR products were cleaned up with ExoSAP-IT (Affimetrix, Cleveland, OH) and *HLA*-*A* and -*C* PCR products were diluted 10-fold and directly sequenced using a set of six sequencing primers per locus as previously described^38^ on a 3730xl Genetic Analyzer (Applied Biosystems, Foster City, CA), using the BigDye Terminator v3.1 chemistry (Life Technologies, Carlsbad, CA). Sequences were trimmed with Sequencing Analysis software v5.4 (Applied Biosystems) and HLA subtypes were assigned using UType v7.1 RUO (Applied Biosystems) using the up-to-date IPD-IMGT/HLA Database (http://www.ebi.ac.uk/ipd/imgt/hla/). Using this procedure, ambiguous HLA assignments can arise due to the presence of polymorphisms outside of the analyzed region (exons 2 and 3). For these cases, ambiguous HLA pairs were managed as G groups, including: A*74:*01*:*01G* (A*74:*01* in the text, encompassing A*74:*01* and A*74:*02*; difference in exon 1), C*18:*01*:*01G* (C*18:*01* in the text, encompassing C*18:*01* and C*18:*02*; difference in exon 5), C*17:*01*:*01G* (C*17:*01* in the text, encompassing C*17:*01*, C*17:*02* and C*17:*03*; difference in exons 1 and 5). Ambiguous HLA assignments may also arise due to the lack of phase resolution as a result of bulk (direct) sequencing of PCR amplicons. Considering only HLA ambiguities that affect the first (allele group-level) and second (subtype-level) HLA fields and that included either two common alleles or one common and one well-documented subtype present in the Common and Well-documented catalogue^39^, a total of 142 *HLA*-*A*, 161 *HLA*-*B*, and 104 *HLA*-*C* pairs were ambiguous due to lack of phase resolution (Supplementary Table S1). These ambiguities were resolved by assigning the most probable combination using HLA allele frequency (AF) and linkage disequilibrium (LD) data from the same cohorts. All HLA haplotypes were confirmed using the same published probabilistic method used for the HOMER cohort^36^ (HLA Completion web tool, http://boson.research.microsoft.com/hla/). Only 72 (of 6392, 1.12%) missing *HLA-A* or *HLA-C* types were imputed due to PCR/sequencing failure at these loci or lack of sample availability, using the same tool. HLA pairs with an unresolved *HLA*-*B* allele (23/3213 pairs, 0.71%) were considered missing data. As previously described^40^, our HLA typing methodology was validated to be 99.9% accurate in Mesoamerican Mestizo populations by comparing assigned HLA types to those obtained via amplification of exons 1 to 8 (for *HLA*-*A* and *HLA*-*C*) and exons 1 to 7 (for *HLA*-*B*) followed by next generation sequencing (TruSight HLA v2 Sequencing Panel, Illumina, San Diego, CA) in a large independent Mexican cohort (n = 323). Raw HLA typing data are available upon direct request to the authors.

### HLA linkage disequilibrium analysis

LD between pairs of HLA alleles was assessed using the Los Alamos HIV Molecular Immunology Database HLA Analysis Tools (https://www.hiv.lanl.gov), with multiple comparisons addressed via Bonferroni correction. For the MEX cohort LD analysis, 12,970 two-way comparisons were performed (p-values < 3.9E-06 were considered significant), and for the CAM cohort 14,234 two-way comparisons were performed (p-values < 3.5E-06 were considered significant). The high-dimensional visualization tool Disentangler^41^ was used to graph HLA haplotype structures.

### HLA allele frequency comparison

HLA AF were calculated by direct gene count (denominator 2n). All HLA subtypes with AF > 0.001 in at least one cohort were compared using two-tailed Fisher’s exact test; here, multiple comparisons were addressed using q-values, the p-value analogue of the false discovery rate^42^. Results with p < 0.05 and q < 0.2 were considered statistically significant.

### Univariable and multivariable analyses of HLA-HIV clinical parameter associations

HLA allele associations with five clinical parameters previously described to be predictive of HIV disease progression were investigated. These included pVL^43^ and absolute CD4 count^44^, used in routine clinical monitoring of HIV infection, as well as CD4 percentage^45^, CD4/CD8 ratio^45^, and a proxy variable called Z-score that combines information from both CD4 count and pVL^46^, calculated as

$${\rm{Z}}{\rm{s}}{\rm{c}}{\rm{o}}{\rm{r}}{\rm{e}}=\frac{[\frac{{\rm{C}}{\rm{D}}4-{\rm{m}}{\rm{e}}{\rm{a}}{\rm{n}}\,{\rm{C}}{\rm{D}}4}{{\rm{s}}{\rm{d}}{\rm{C}}{\rm{D}}4}]-[\frac{{\rm{p}}{\rm{V}}{\rm{L}}-{\rm{m}}{\rm{e}}{\rm{a}}{\rm{n}}\,{\rm{p}}{\rm{V}}{\rm{L}}}{{\rm{s}}{\rm{d}}{\rm{p}}{\rm{V}}{\rm{L}}}]}{2}$$

(sd = standard deviation), where lower pVL and higher CD4 counts yield higher and more positive Z-scores. MEX and CAM cohorts were analyzed separately and in a combined fashion, where all HLA alleles observed in a minimum of 5 individuals (representing AF ≥ 0.0015 and ≥0.0008 for the individual and combined cohorts, respectively) were evaluated. This encompassed 147 HLA alleles (42 *HLA*-*A*, 75 *HLA*-*B* and 30 *HLA*-*C*) in the pooled MEX/CAM analysis; 119 HLA alleles (33 *HLA*-*A*, 59 *HLA*-*B* and 27 *HLA*-*C*) in the MEX analysis alone and 123 (35 *HLA*-*A*, 60 *HLA*-*B* and 28 *HLA*-*C*) in the CAM cohort alone. The Mann-Whitney U test was used to evaluate associations between each HLA allele (treated as a binary variable; *e*.*g*. comparing B*57:*01*^+^ vs. B*57:*01*^−^ individuals) and the parameter of interest. Univariable analysis was also performed using Generalized Linear Models (GLM) to estimate HLA-HIV association effect sizes. Multiple comparisons were addressed using q-values^47^, where associations with p-values < 0.05 and q-values < 0.2 were considered significant. We additionally instituted a scoring system where, for each HLA allele investigated, we summed its total number of significant protective and risk associations (each assigned + 1 and −1, respectively), such that final scores could range from +5 to −5. Alleles with no significant associations were assigned a score of 0. Two multivariable linear regression models were also constructed to account for potential confounders of HLA-HIV associations, after which the overall 5-parameter scores were adjusted accordingly. First, independent models were constructed relating each HLA allele to each HIV clinical parameter, while adjusting for gender, age, geographical origin (country/region coded as n-1 binary variables) and the effect of the most significant HLA associations for that parameter (defined as the HLA alleles with p < 0.001 in the corresponding Mann-Whitney univariable analysis). Specific HLA alleles included in each model are listed in Supplementary Table S9. Second, we constructed models adjusting for all HLA subtypes in significant (p < 0.05 and q < 0.2) linkage disequilibrium with every HLA allele associated with an HIV clinical parameter in that cohort. Statistical analyses were undertaken using Stata/MP v14.1 (StataCorp, College Station, TX) and R v3.3.3^48^.

## Results

### MEX and CAM cohort characteristics

Overall, the clinical and demographic characteristics of the Mexico (MEX; n = 1679), Central America (CAM; n = 1534) and combined (n = 3213) cohorts (Table 1) are consistent with the frequent diagnosis of HIV in advanced infection in Latin America and an concentrated epidemic mainly in men who have sex with men^49^. Median pVL in MEX was significantly higher (p < 0.00001) than that in CAM (4.72 versus 4.57 Log_10_ HIV RNA copies/mL), though CD4 T cell counts and CD4/CD8 ratios did not differ significantly between the cohorts (median 315 cells/µL and ~0.28, respectively). CD4+ T cell percentages and Z-scores^46^ also differed marginally between cohorts (p < 0.01).

**Table 1:** Clinical and demographic characteristics of the Mesoamerican cohorts.

	Pooled MEX/CAM cohort	MEX cohort	CAM cohort	p-value*
N	3213	1679	1534	—
Age (years, median[IQR])	31 [25–40]	30.5 [24–38]	33 [26–42]	<0.00001
Female (N[%])	912 [28.4%]	364 [21.7%]	548 [35.7%]	0.0001
Log_10_ HIV Plasma Viral Load (RNA copies/mL, median[IQR])	4.65 [4.01–5.22]	4.72 [4.14–5.26]	4.57 [3.83–5.14]	<0.00001
CD4+ T cell count (cells/μL, median[IQR])	315 [124–516]	315 [124–528]	315 [125–504]	NS
HIV Z-score (median[IQR]) β	−0.08 [−0.66–0.52]	−0.12 [−0.69–0.46]	−0.05 [−0.61–0.56]	0.0022
Percentage of CD4+ T cell counts (%[IQR])	16.0 [9.0–24.0]	16.5 [9.0–24.8]	16.0 [9.0–23.0]	0.0056
CD4/CD8 ratio (median[IQR])	0.28 [0.14–0.49]	0.28 [0.14–0.49]	0.29 [0.14–0.49]	NS
Marital status (N[%])				0.0001
Single	1434 [63.5]	538 [72.2]	896 [59.2]	
Married	325 [14.3]	91 [12.2]	234 [15.4]	
Domestic partnership	499 [22.1]	116 [15.5]	383 [25.3]	
Unknown	955	934	21	
Education (N[%])				0.0001
Illiterate	136 [6.0]	16 [2.1]	120 [7.9]	
Elementary	704 [31.2]	127 [17.3]	577 [38.0]	
High school	863 [38.3]	345 [47.0]	518 [34.1]	
Degree or technical qualification	523 [23.2]	233 [31.7]	290 [19.1]	
Postgraduate	26 [1.1]	13 [1.7]	13 [0.8]	
Unknown	1097	945	16	
Employment (N[%])				0.0023
Unemployed	969 [44.0]	282 [40.0]	687 [45.9]	
Employed	1077 [48.9]	358 [50.85]	719 [48.0]	
Student	154 [7.0]	64 [9.0]	90 [6.0]	
Unknown	1013	975	38	
HIV risk factor (N[%])				0.0001
Heterosexual	1394 [64.2]	289 [42.8]	1105 [73.9]	
Men who have sex with men	657 [30.2]	322 [47.7]	335 [22.4]	
Bisexual	75 [3.4]	37 [5.4]	38 [2.5]	
People who inject drugs	35 [1.6]	19 [2.8]	16 [1.0]	
Blood transfusion	5 [0.2]	5 [0.7]	0 [0]	
Mother-to-child transmission	3 [0.1]	2 [0.3]	1 [0.07]	
Unknown	1044	1005	39	

β Metric relating pVL and CD4 count. Higher Z-score values mean lower pVL and higher CD4 count and vice versa. *Mann-Whitney U test or Chi-squared test were used to compare values between the Mexico and Central America cohorts.

### Unique immunogenetic profiles of HIV-infected individuals in Mesoamerica

**Figure 1:**
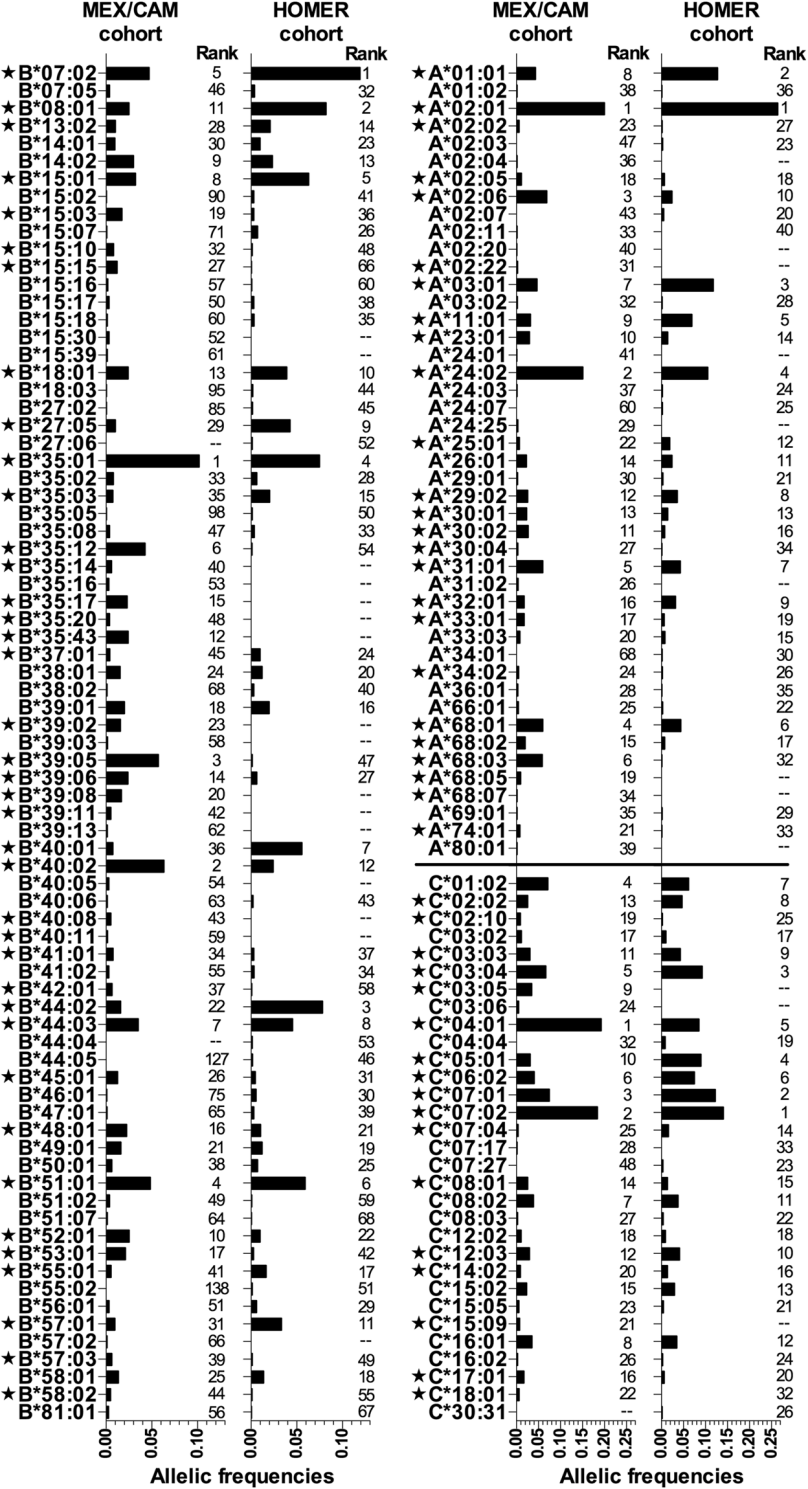
Comparison of HLA class I allele frequencies between the Mestizo MEX/CAM cohort (n = 3213) and the mainly Caucasian HOMER cohort (n = 1622). Allele frequencies (2n) were calculated using the HLA Analysis tool from Los Alamos HIV Database (https://www.hiv.lanl.gov); all HLA AF > 0.001 in at least one cohort are shown here. AF were compared using Fisher’s exact test, with multiple tests addressed using q-values^42^. Significant differences (p < 0.05, q < 0.2) are denoted by a star.

HLA class I allele frequencies differed markedly in the combined MEX/CAM versus HOMER cohorts (Fig. 1). The most frequent *HLA*-*A*, -*B* and -*C* alleles observed in MEX/CAM were A*02:*01* (AF = 0.20), A*24:*02* (0.15), A*02:*06* (0.06), B*35:*01* (0.10), B*40:*02* (0.06), B*39:*05* (0.05), C*04:*01* (0.19), C*07:*02* (0.18) and C*07:*01* (0.07). Of the 153 alleles investigated, 52.9% (81/153) exhibited significantly (p < 0.05, q < 0.2) different frequencies between MEX/CAM and HOMER; these included 25/45 *HLA*-*A*, 39/77 *HLA*-*B* and 17/31 *HLA*-*C* alleles. As expected, Amerindian HLA alleles^27,28^ were enriched and Caucasian HLA alleles^50^ were underrepresented in MEX/CAM compared to HOMER. Notably, MEX/CAM featured a diversity of “typical” Amerindian HLA subtypes belonging to the A*02, A*68, B*15, B*35, B*39, and B*40 allele groups.

**Figure 2:**
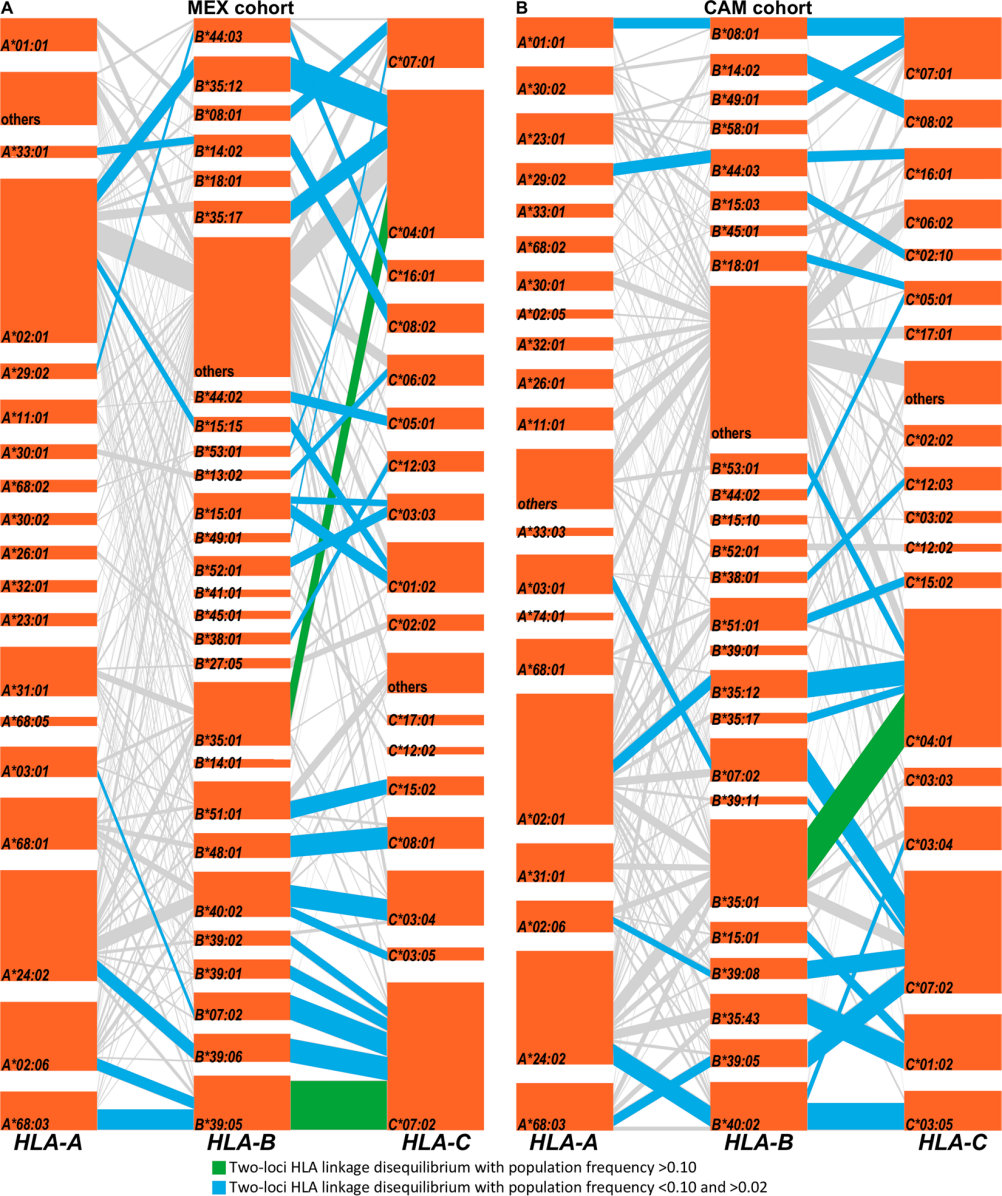
HLA class I haplotype structures and linkage disequilibrium in the MEX (panel A) and CAM (panel B) cohorts. HLA loci are stacked vertically, with each orange tile representing a specific HLA subtype, and with segments connecting linked alleles on adjacent loci. The height of each tile and the thickness of each segment correspond to HLA allele and haplotype frequencies, respectively. The most frequent HLA allele pairs (two-loci) found to be in linkage disequilibrium are highlighted in green (PF > 0.10) and blue (PF < 0.10 and > 0.02); less frequent pairs (PF < 0.02) are shown in grey. Frequently linked (PF < 0.10 and > 0.02) *HLA*-*A* and *HLA*-*C* allele pairs were also found in our cohorts including A*33:*01*/C*08:*02*, A*29:*02*/C*16:*01*, A*68:*01*/C*03:*04*, and A*68:*03*/C*07:*02* in the MEX cohort, and A*02:*06*/C*07:*02*, A*24:*02*/C*01:*02*, A*24:*02*/C*03:*05*, and A*68:*03*/C*07:*02* in the CAM cohort (not shown in the figure; see Supplementary Tables S4 and S6).

Strong HLA LD allowed us to identify common 2 and 3-loci haplotypes in our populations (Fig. 2 and Supplementary Tables S2-S7). A total of 104 distinct 2-loci HLA haplotypes were identified in MEX (Bonferroni, p < 3.9E-06; Supplementary Table S2), of which two had population frequency (PF) ≥ 0.10 (B*39:05~C*07:*02* [PF = 0.13^51^] and B*35:01~C*04:*01* [PF = 0.12]); a further 35 had PF between 0.02–0.10 (Fig. 2A). Also, 36 distinct 3-loci HLA haplotypes were identified in MEX (Bonferroni, p < 8.0E-06; Supplementary Table S3). In CAM, 95 distinct 2-loci HLA haplotypes were identified (Bonferroni p < 3.5E-06; Supplementary Table S4), of which one had PF ≥ 0.10 (B*35:01~C*04:*01* [PF = 0.13] and 33 had PF between 0.02–0.10 (Fig. 2B). A further 30 distinct 3-loci HLA haplotypes were identified in CAM (Bonferroni, p < 8.2E-06; Supplementary Table S5). Analyzing MEX/CAM as a whole, 154 distinct 2-loci and 87 distinct 3-loci were identified (Bonferroni p < 2.3E-06 and p < 4.2E-06, respectively; Supplementary Tables S6–S7).

Overall, these findings highlight the immunogenetic uniqueness of Mesoamerican Mestizo populations and support them as ideal for identifying novel HLA correlates of HIV control.

### HLA associations with HIV pVL in MEX/CAM include both canonical and novel associations

**Figure 3:**
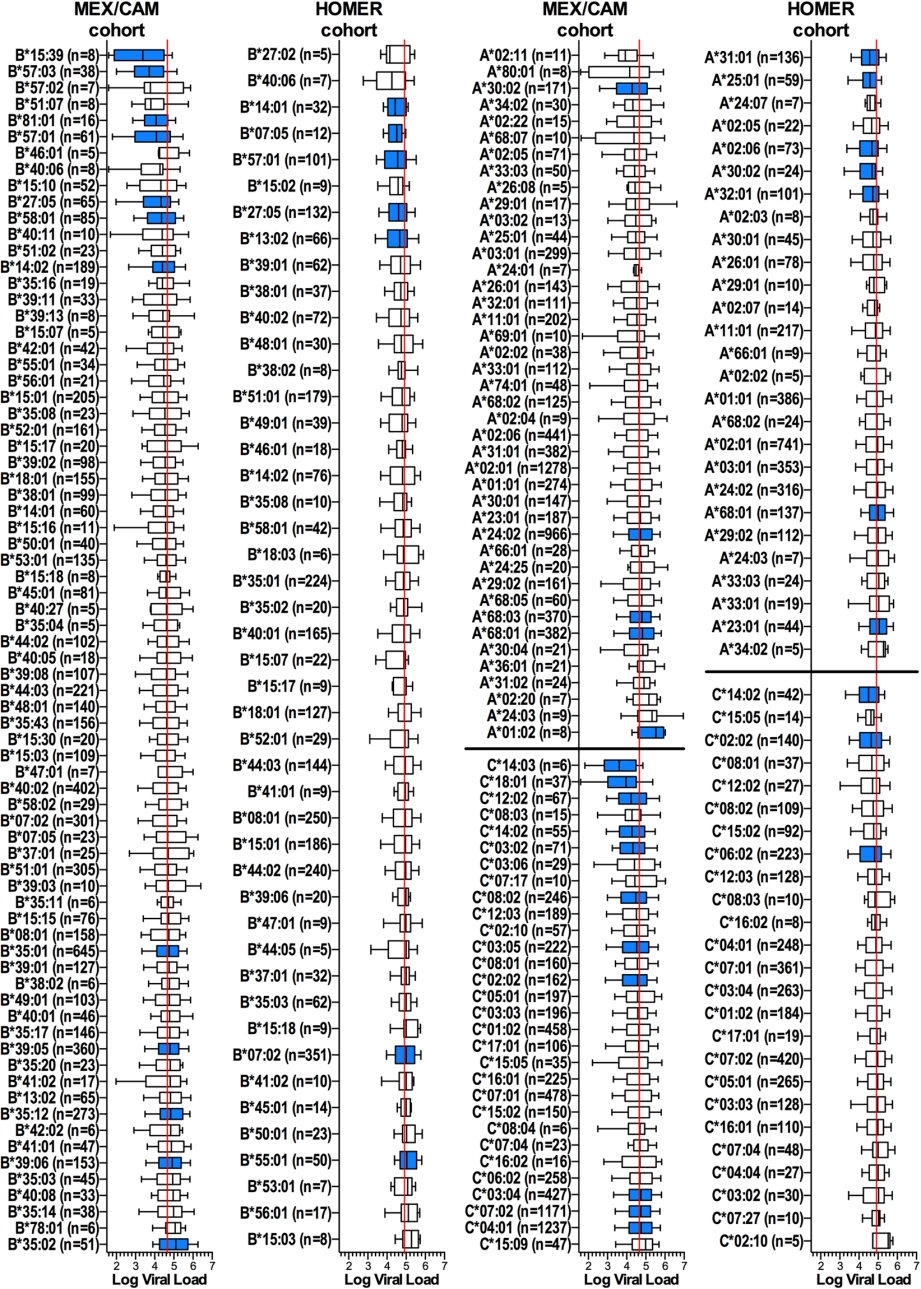
HLA associations with HIV pVL in the MEX/CAM and HOMER cohorts. Associations between HLA subtypes and pVL were investigated for HLA alleles with frequency equal or greater than 5 in HIV-1 clade B-infected ART-naïve individuals from the MEX/CAM (n = 3213) and predominantly Caucasian HOMER (n = 1622) cohorts. Associations between HLA alleles and pVL were evaluated using the Mann-Whitney U test, with multiple tests addressed using q-values. Significant (p < 0.05, q < 0.2) associations are highlighted in blue. Boxes denote median, 25^th^ and 75^th^ percentile, and whiskers represent the 10–90^th^ percentile of pVL distributions of individuals expressing each *HLA*-*B* allele (left panels) and *HLA*-*A*/*C* allele (right panels). HLA alleles are ordered by their pVL median and the number (n) of individuals expressing each HLA allele is shown. Red vertical lines denote plasma viral load medians for each cohort.

Given the significantly different HLA frequency distributions between MEX/CAM and HOMER, we hypothesized that HLA associations with HIV parameters would also differ markedly. We explored this by identifying HLA allele associations with the well-characterized marker of HIV disease progression pVL^43^ in both cohorts, in a univariable analysis. At the predefined statistical threshold of q < 0.2, we identified thirteen HLA alleles (5 *HLA*-*A*, 5 *HLA*-*B*, and 3 *HLA*-*C*) significantly associated with lower pVL, and four alleles (2 *HLA*-*A*, and 2 *HLA*-*B*) associated with higher pVL in HOMER, compared to sixteen HLA alleles (1 *HLA*-*A*, 7 *HLA*-*B*, and 8 *HLA*-*C*) associated with lower pVL, and twelve alleles (4 in *HLA*-*A*, 5 in *HLA*-*B*, and 3 in *HLA*-*C*) associated with higher pVL in MEX/CAM (Fig. 3). A number of these associations were consistent across cohorts: in all cases these were HLA alleles previously reported to be associated with HIV progression, including the canonical protective alleles B*57:*01* and B*27:*05*^1,9,11,12,16,20,21,23–25^ which were associated with significantly lower pVL in both cohorts. Additionally, as previously described, A*30:*02*^14,24,25^ (LD with B*18:*01* and C*05:*01* in both cohorts), C*02:*02*^1,23^ (LD with B*27:*05* in both cohorts) and C*14:*02*^5,23^ (LD with B*51:*01* in both cohorts) were associated with lower pVL in both cohorts; while A*68:*01*^17,20^ (LD with C*03:*04* in MEX/CAM and with C*07:*04* in HOMER) was associated with higher pVL in both cohorts. Numerous previously-reported HLA associations were also confirmed in one of the two cohorts. Specifically, A*25:*01*^3,11^, A*32:*01*^1,11^, B*14:*01*^3,16^, and B*13:*02*^1,17,24,25^ were associated with lower pVL exclusively in HOMER; while B*07:*02*^1,3,17,24,25^, B*55:*01*^52,53^, and A*23:*01*^11,18^ were associated with higher pVL in HOMER. Moreover, B*14:*02*^3,16^, B*57:*03*^5,8,15–17,19,23^, B*58:*01*^5,12,14,17,20,22^, B*81:*01*^5,12,17,20^, C*03:*02*^17^ (LD with B*58:*01*), C*03:*05*^24^ (LD B*40:*02*, A*24:*02*), C*08:*02*^20^ (LD B*14:*01*/*02*), C*12:*02*^20,22^ (LD B*52:*01*^3^), C*14:*03*^22,23^ (no significant LD found), and C*18:*01*^14,17,20,25^ (LD B*57:*01*/*02* and B*81:*01*) were associated with lower pVL only in MEX/CAM; while B*35:*01*^5,6,11,13,22,23^, B*35:*02*^1,6,9,11,13,23^ (which exhibited the highest median pVL of all *HLA*-*B* alleles), A*24:*02*^1,11,18^ (LD B*39:*06*, B*40:*02*, C*03:*05*), C*04:*01*^6,16,22,23^ (LD B*35:*01*/*02*/*08*/*12*/*14*/*16*/*17*/*20*, B*07:*02*, B*53:*01*, among others), and C*07:*02*^1,14^ (LD B*39:*01*/*05*/*06*/*08*/*11*, B*07:*02*, among others) were associated with higher pVL in MEX/CAM.

Numerous novel associations were also identified in MEX/CAM. These included the relatively rare Amerindian allele B*15:*39* (n = 8, p = 0.0052, q = 0.04), which was associated with lower pVL, as well as the common Amerindian alleles B*35:*12* (n = 273, p = 0.0002, q = 0.003), B*39:*05* (n = 360, p = 0.0026, q = 0.0034), B*39:*06* (n = 153, p = 0.011, q = 0.07), A*68:*03* (n = 370, p = 0.0079, q = 0.061; LD B*39:*05*, B*35:*43*, C*07:*02*), and C*03:*04* (n = 427, p = 0.0339, q = 0.15; LD B*40:*02*/*01*/*05*/*08*/*11*, A*68:*01*), which were associated with significantly higher pVL (Fig. 3). Notably, these alleles, though present at relatively high frequency in MEX/CAM, are rare or absent in Caucasian and African populations (e.g. Fig. 1 and ref.^50^).

### Additional novel associations between Amerindian HLA alleles and HIV risk/protection

We next extended our analyses of our Mesoamerican cohorts to identify HLA alleles associated with an expanded panel of five HIV clinical parameters (pVL, CD4 count, Z-score, %CD4, and CD4/CD8 ratio). HLA alleles associated with each of the 5 clinical parameters, stratified by cohort, are listed in Table 2. Consistent with previous reports^12^, *HLA-B* alleles featured prominently among these associations, with 43 *HLA*-*B* alleles (24 in MEX/CAM, 11 in MEX, and 8 in CAM) associated with at least one clinical parameter (p < 0.05, q < 0.2) compared to 29 *HLA*-*A* alleles (13 in MEX/CAM, 5 in MEX, and 11 in CAM) and 29 *HLA*-*C* alleles (14 in MEX/CAM, 9 in MEX, and 6 in CAM).

**Table 2:** Summary of univariable analysis stratified by cohort.

Number of alleles that were associated with at least one HIV clinical parameter			
HLA loci	MEX/CAM cohort	MEX cohort	CAM cohort
*HLA*-*A*	13	5	11
*HLA*-*B*	24	11	8
*HLA*-*C*	14	9	6
**Number of associations by HIV clinical parameter**			
HIV clinical parameter	**MEX/CAM cohort**	**MEX cohort**	**CAM cohort**
Plasma Viral Load	28	14	11
CD4 count	30	14	18
HIV Z-score	30	15	16
%CD4	27	13	10
CD4/CD8 ratio	26	14	12

The number of HLA-HIV associations are shown by HLA loci (top) and by HIV clinical parameter (bottom).

**Figure 4:**
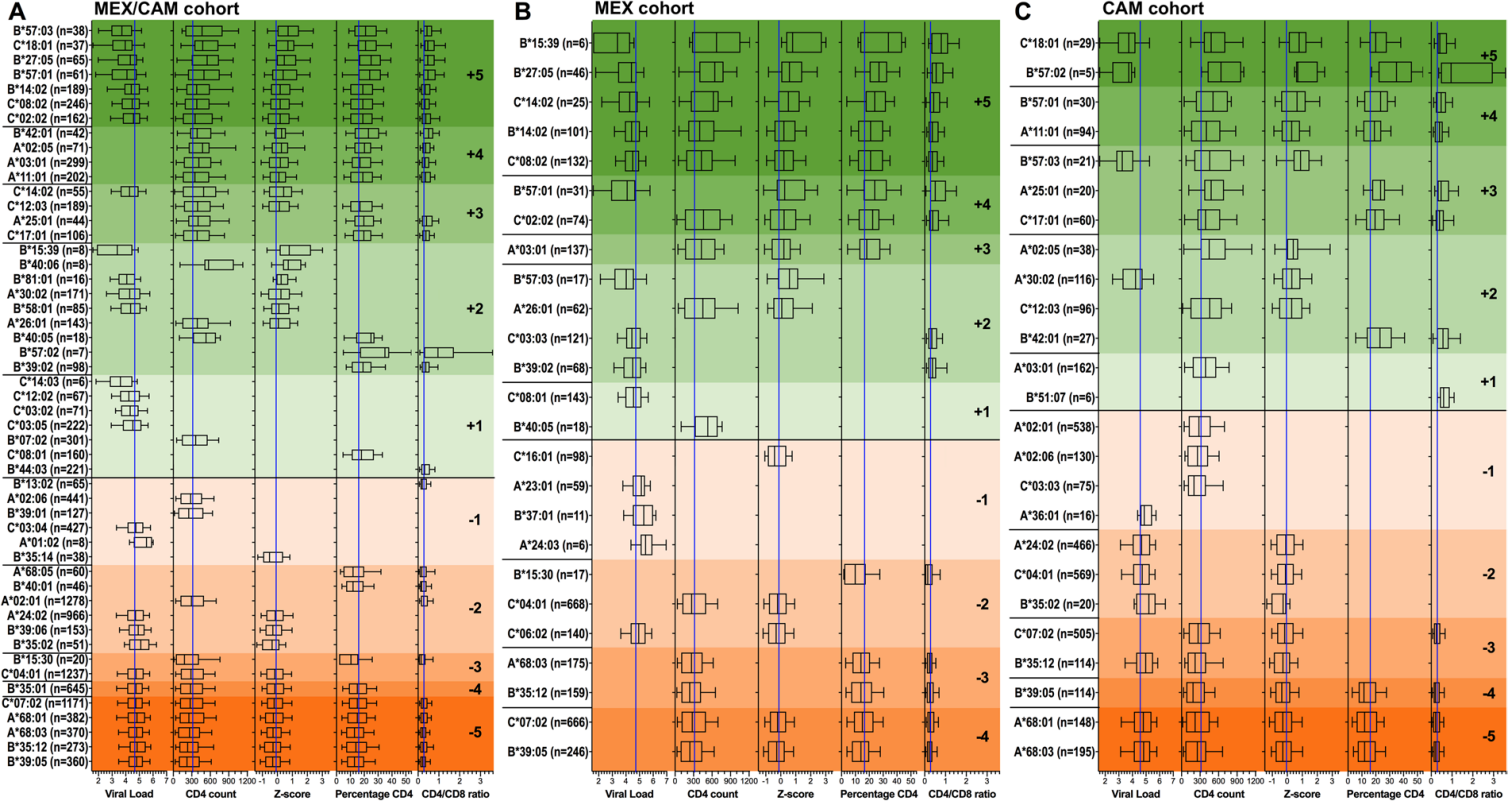
HLA-HIV associations in Mesoamerican cohorts using 5 HIV clinical parameters (univariable analysis). Associations between the expression of HLA class I alleles and 5 HIV clinical parameters (pVL, CD4 count, Z-score, CD4% and CD4/CD8 ratio) were investigated for alleles with frequency equal or greater than 5 in HIV-1 clade B-infected ART-naïve individuals from the pooled MEX/CAM cohort (**A**), only in MEX cohort (**B**) or only in CAM cohort (**C**). Associations were evaluated using the Mann-Whitney U test and multiple tests were addressed using q-values. Boxplots of only significant (p < 0.05, q < 0.2) HLA-HIV associations are shown. Alleles are grouped by HLA-HIV score ( + 5 to −5), then ordered by the median of Z-score, pVL, CD4 count, %CD4 and CD4/CD8 ratio. Protective and risk alleles are shaded with progressively deeper green and orange colors, respectively. Boxes denote the median, 25^th^ and 75^th^ percentile of the HIV clinical parameter of interest; whiskers represent the 10–90^th^ percentile. The number (n = ) of individuals expressing each HLA allele is shown. Blue vertical lines denote cohort median values for each parameter.

As a novel way to quantify HLA associations with HIV clinical parameters, we instituted a scoring system that summed each HLA allele’s total number of significant protective and risk associations (each assigned +1 and −1, respectively), such that final scores ranged from +5 to −5 (Fig. 4). Alleles with no significant associations were assigned a score of 0. As expected, a given HLA allele’s overall association score correlated significantly in a dose-dependent manner with its associated median value for all 5 clinical parameters in both MEX/CAM and individual cohorts (Supplementary Figure S1); also, HLA subtypes associated with pVL (Fig. 3) generally tended to be associated with other clinical parameters (e.g. 22 of 28, 78.5% in MEX/CAM), though some exceptions were noted (Fig. 4).

In the MEX/CAM cohort, 7 HLA alleles (B*57:*03*, C*18:*01* [LD B*57:*02*/*03*, B*81:*01*], B*27:*05*, B*57:*01*, B*14:*02*, C*08:*02* [LD B*14:*01*/*02*], and C*02:*02* [LD B*27:*05]*) achieved the highest protective score of +5 (Fig. 4A). The next highest-scoring group (+4) included B*42:*01*, A*02:*05* (LD B*50:*01*, B*58:*01*), A*03:*01* (new association, LD B*07:*02*, B*39:*05*), and A*11:*01* (LD B*39:05); in all cases these alleles were significantly associated with all clinical variables except pVL. A total of 20 additional HLA alleles scored between +1 and +3; these included Amerindian alleles B*15:*39* (LD C*03:*03*) and B*39:*02* (LD C*07:*02*), both scoring +2, identifying these as novel protective alleles.

Similarly, 3 of the 5 HLA alleles that achieved the most negative score (−5) in MEX/CAM were Amerindian alleles; namely B*39:*05* (LD C*07:*02*, A*68:*03*, A*02:*06*, A*23:*01*), B*35:*12* (LD C*04:*01*, A*02:*01*) and A*68:*03* (LD B*39:*05*, C*07:*02*) (Fig. 4A), identifying these as novel risk alleles. A total of 15 additional HLA alleles scored between −4 and −1; among these Amerindian alleles B*15:*30* (LD C*01:*02*, *score* -*3*), B*39:*06* (LD C*07:*02*, A*24:*02; score* -*2*), A*68:*05* (*score* -*2*), and B*35:*14*, C*03:*04* (LD B*40:*02*/*01*/*05*/*08*/*11*, A*68:*01*), B*39:*01* (LD C*07:*02*, C*12:*03*) and A*02:*06* (LD B*39:*05*, C*07:*02*), (score -1), were also identified as novel risk alleles. Additional risk alleles included B*35:*01* (score -4, LD C*04:*01*, C*07:*01*, C*08:*02*, C*01:*02*), previously described as detrimental in some settings^5,6,11,13,22,23^ and B*13:*02* (score -1, LD C*06:*02*, A*30:*01*) previously described as protective^1,17,24,25^. Analysis of MEX and CAM cohorts separately yielded results that corroborated the pooled analysis, but also revealed a small number of additional associations (Fig. 4B,C). In MEX, the latter included previously reported risk associations between HLA alleles and HIV disease progression including C*16:*01*^20^, A*23:*01*^11,18^ and B*37:*01*^11,22^ (all scoring -1) as well as a novel risk association with A*24:*03* (score -1). In CAM, these included a protective association with B*51:*07*^23^ (score + 1), and risk associations with C*03:*03*^22^ and A*36:*01*^5,24^ (both scoring −1).

To further quantify effects of HLA alleles on HIV parameters, we repeated univariable analyses using generalized linear models (Supplementary Table S8). HLA-HIV scores derived from the GLM analysis were highly concordant with those obtained using the Mann-Whitney U test (Spearman rho >0.9 in the combined and individual cohorts, in all cases p < 0.0001, Supplementary Figure S2).

**Figure 5:**
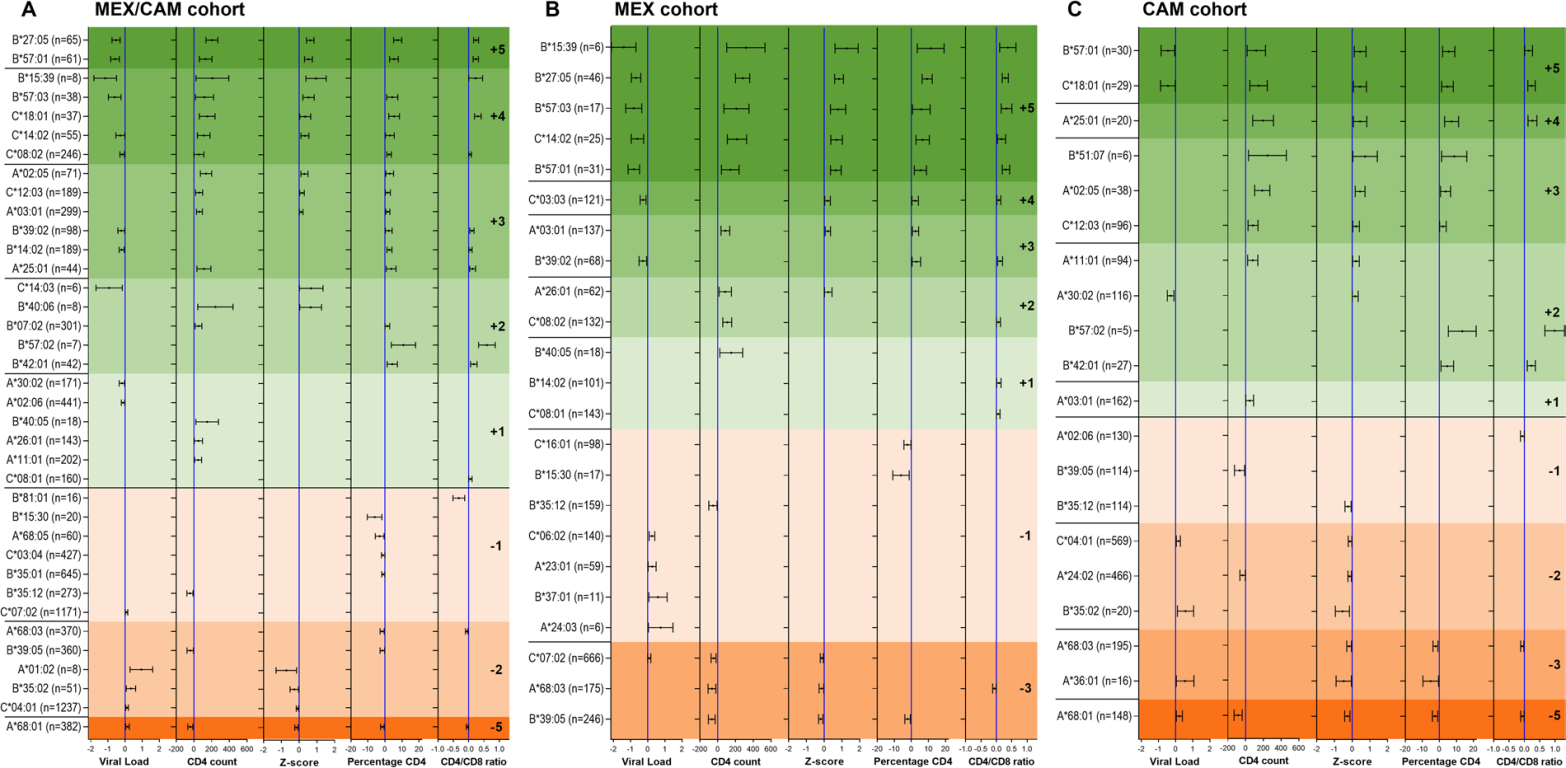
Multivariable analysis of HLA-HIV associations in Mesoamerican cohorts using 5 HIV clinical parameters. An independent linear regression model (GLM) was constructed for each HLA allele and clinical parameter, while adjusting for gender, age, geographical origin (country or region) and the presence of HLA alleles with p < 0.001 in the Mann-Whitney univariable analyses for each clinical parameter; see Supplementary Table S9 for specific HLA alleles adjusted for in each model). Coefficients and 95% confidence intervals (CI) of significant (p < 0.05, q < 0.2) associations are shown. Alleles are grouped by HLA-HIV score (+5 to −5), then ordered by the coefficient of Z-score, pVL, CD4 count, %CD4 and CD4/CD8 ratio. Protective and risk alleles are shaded with progressively deeper green and orange colors, respectively. The number (n = ) of individuals expressing each HLA allele is shown. Blue vertical lines denote a coefficient equal to zero.

**Figure 6:**
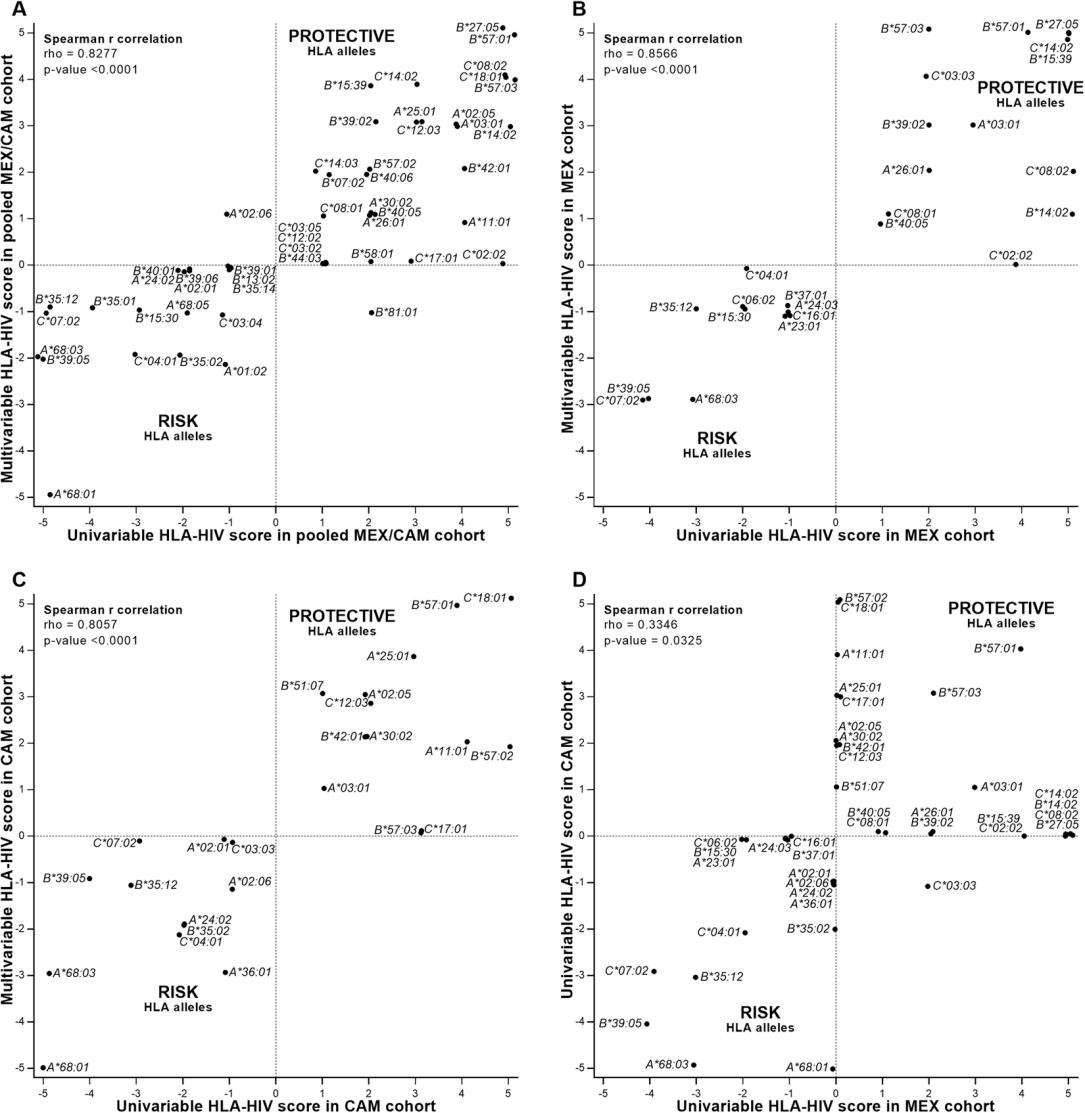
Correlation of 5-parameter HLA-HIV association scores within and between cohorts Scatter plot of univariable (Mann-Whitney) and multivariable HLA-HIV scores in the combined MEX/CAM (**A**), MEX (**B**) and CAM (**C**) cohorts. Panel (D) shows a scatter plot of univariable HLA-HIV scores between MEX and CAM cohorts. Correlation between HLA-HIV scores were determined using Spearman’s rank test. Random jittering was used to prevent dots being superimposed. For A, B & C panels only HLA alleles with at least one association with a HIV clinical parameter were considered.

We next performed a multivariable analysis to correct for gender, age, geographic location of recruitment, as well as HLA alleles with the most significant associations for each HIV clinical parameter in the univariable analysis in both pooled and individual cohorts. Resulting regression coefficients and 95% confidence intervals are shown in Fig. 5 and Supplementary Table S9. Overall, HLA-HIV scores of univariable and multivariable analyses were highly concordant in all cohorts (Spearman’s rho 0.8277, 0.8566, and 0.8057 in MEX/CAM, MEX and CAM respectively, all p < 0.0001; Fig. 6A–C). Importantly, the majority (10/15, 66.6%) of novel Amerindian HLA-HIV associations identified in the MEX/CAM univariable analyses remained significant following adjustment for these parameters; these included A*03:*01*, B*15:*39*, and B*39:*02* as protective alleles and A*01:*02*, A*68:*03*, A*68:*05*, B*15:*30*, B*35:*12*, B*39:*05* and C*03:*04* as risk alleles. When cohorts were analyzed separately, A*03:*01* (protective), A*68:*03*, B*35:*12*, and B*39:*05* (risk) remained significant after multivariable adjustment in both cohorts.

A second multivariable analysis was performed to account for HLA LD effects (Supplementary Table 10). The majority of HLA-HIV associations identified in univariable analyses of the pooled MEX/CAM cohort remained significant after multivariable correction: these included the novel protective alleles A*03:*01* (LD B*07:*02*, B*39:*05*), B*15:*39* (LD C*03:*03*) and B*39:*02* (LD C*07:*02*), and the detrimental A*01:*02* (LD B*49:*01*), A*02:*06* (LD C*07:*02*, B*39:*05*, B*39:*08*, C*08:*01*), A*68:*03* (LD B*39:*05*, C*07:*02*, B*35:*43*), B*15:*30* (LD C*01:*02*), B*35:*12* (LD C*04:*01*, A*02:*01*), and C*03:*04* (LD B*40:*01*/*02*, B*15:*10*, B*40:*05*/*08*/*11*, A*68:*01*) alleles. However, in some cases, the very strong LD between certain allele pairs (notably the protective B*14:02~C*08:02 haplotype, which exhibited the strongest LD in the cohort [p = 8.0E-195], the risk B*39:05~C*07:*02* haplotype [LD p = 3.9E-144], and the risk B*40:01~C*03:*04* haplotype [LD p = 2.6E-23]) precluded identification of the allele driving the association. Moreover, several *HLA*-*C* associations (e.g. C*02:*02* as protective and C*04:*01* as risk) could be partially or completely explained by their LD with *HLA*-*B* alleles: e.g. B*27:*05* for C*02:*02*, and B*35:*01*/*02*/*12* for C*04:*01* (the apparent effect of C*04:*01* in strong LD with B*35 alleles has been resolved previously^9^). Furthermore, some *HLA*-*B* alleles did not remain significant after LD correction. Among these were the new Amerindian risk alleles B*39:*01* and B*39:*06*, which are both in strong LD with C*07:02. As the latter allele is also in extremely strong LD with the risk and highly frequent allele B*39:*05*, it is possible that inclusion of C*07:*02* in the model confounds our ability to validate B*39:*01* and B*39:*06* as independent risk factors.

### Secondary analyses using only pVL and CD4 count

Although the use of the 5-parameter scoring system enhanced sensitivity to identify associations (see discussion), HLA/HIV association studies have traditionally used pVL and CD4 count only^5,11,17,20^. To facilitate direct comparison of our results to previous studies, results based on pVL and CD4 count only are provided in Supplementary Tables S8–S9. Correlation between the results of the 5- and 2-parameter scoring systems were robust for both univariable (all rho >0.93, in all cases p < 0.0001; Supplementary Figure S3, panels A-C) and multivariable analyses (all rho > 0.88, in all cases p < 0.0001; Supplementary Figure S3, panels D-F).

### Regional variation in HLA-HIV associations between MEX and CAM cohorts

Finally, we wished to investigate the extent to which HLA-HIV associations in MEX and CAM were universal versus region-specific. Taking the union of all HLA alleles that achieved a non-zero score in univariable analyses in either the MEX and/or CAM cohorts, we assessed the degree to which their scores correlated between cohorts using Spearman’s correlation. Overall, the relationship was statistically significant but the strength of the correlation was rather modest (Spearman rho = 0.334, p = 0.032, Fig. 6D). A total of 8 associations were shared between the MEX and CAM cohorts (protective: B*57:*01*, B*57:*03*, and A*03:*01*; detrimental: B*39:*05*, A*68:*03*, C*07:*02*, B*35:*12*, and C*04:*01*), while 16 were exclusive to MEX and 16 to CAM. It is important to note that these differences could be attributable, at least in part, to the significant HLA AF distribution differences between cohorts (Supplementary Figure S4), which in turn influences power to identify individual HLA associations below a predefined significance threshold. Nevertheless, this observation suggests that, while many HLA-HIV associations are common to both regions, others may be region-specific.

## Discussion

In the present study, the largest and most comprehensive of its kind undertaken to date in the unique and highly genetically admixed Latin American Mestizo population, we characterized HLA allele frequency distributions, haplotype structures and identified both canonical and novel associations between HLA alleles and HIV control. To accomplish the latter, we implemented a novel scoring system based on five partially interrelated clinical parameters, an approach that allowed more nuanced classification of the consistency of associations observed. Indeed, despite the cross-sectional nature of our study and the fact that we investigated these associations during chronic HIV infection, we readily detected numerous canonical associations with HIV control (*e*.*g*. in MEX/CAM analysis: B*57:*01*/*03*, B*27:*05* and B*14:*02*; in MEX: B*14:*02* and B*27:*05*; in CAM: B*57:*02*; all with +5 score), even though the frequencies of some of these alleles are significantly lower in MEX/CAM than in other global populations. The 5-parameter scoring system also allowed us to detect known protective alleles that were not identified using pVL and CD4 only, including B*42:*01*^5,17,20^, B*57:*02*^17,26^ and B*81:*01*^5,12,17,20^; furthermore it allowed us to distinguish strong association effects from weaker ones (e.g. all canonical protective associations, including B*57:*01*, B*57:*03* and B*27:*05* were associated with all 5 parameters). Confirmation of numerous other HLA associations previously reported in other HIV clade B-infected populations (e.g. B*57:*01*^1,3,9,11,16,21,23^, B*57:*03*^16^, B*27:*05*^1,3,9,11,23^, B*44:*03*^22^, B*58:*01*^22^, B*14:*02*^3,16^, A*25:*01*^1,11^, B*35:*01*^3,22^, B*35:*02*^1,6,9,11,23^) supports the accuracy of our analysis, and increases confidence in the novel associations found. Among the latter, HLA alleles enriched in Amerindian populations featured prominently, particularly among the risk alleles. These included A*03:*01*, B*15:*39* and B*39:*02* (identified as protective) and A*01:*02*, A*24:*03*, A*68:*03*/*05*, B*15:*30*, B*35:*12*/*14*, B*39:*01*/*06*, B*39:05~C*07:*02*, and B*40:01~C*03:*04* (identified as risk). Importantly, most protective and risk HLA alleles remained significant after accounting for potential confounding factors including age, gender, geographical location, HLA alleles with significant effects for each HIV clinical parameter, and HLA linkage disequilibrium, adding further confidence to our findings.

Of note, many of the novel risk HLA alleles identified in the present study are relatively common in Mesoamerica. Indeed, 35.2% (1133/3213) individuals in the MEX/CAM cohort, 40.2% (676/1679) in the MEX cohort and 29.7% (457/1534) in the CAM cohort expressed at least one risk HLA allele. Prevalence of risk alleles was particularly high in Guatemala (48%), El Salvador (42%) and the Southeastern Mexican states of Campeche, Chiapas, Quintana Roo, Tabasco, Veracruz and Yucatan (overall 48%). These observations underscore the importance of implementing and sustaining culturally and locally-appropriate HIV prevention, diagnostic and early antiretroviral treatment programmes, particularly in regions characterized by populations with high Amerindian admixture.

Interestingly, both protective and risk HLA subtypes were identified within the common and genetically diverse Amerindian *HLA*-B*39 allele group, including B*39:*02* (protective) and B*39:*05*, B*39:*01* and B*39:*06* (risk). HIV control by B*39:*02* might be explained by the presence of Glutamine in position 63 and Serine in position 67 (*HLA*-*B* amino acid positions previously associated with HIV control in genome-wide association studies^3,54^) within the HLA peptide binding groove, which distinguish this allele from other *HLA*-B*39 molecules. It is well-established that a difference of only one or two amino acids can influence the risk/protective status of closely-related HLA subtypes (e.g. between B*42:*01* and B*42:*02*; B*57:*03* and B*57:*02*; B*35:*01* and B*35:*02*/*03*; B*44:*02* and B*44:*03*^9,55–63^), suggesting that a similar mechanism could underlie the differences between *HLA*-B*39 subtypes. Of note, B*39:*02* attained a protective score of +2 in univariable (%CD4, CD4/CD8) and +3 in multivariable (pVL, %CD4, CD4/CD8) analyses in the pooled (MEX/CAM) and +2 in univariable (pVL, CD4/CD8) and +3 in multivariable (pVL, %CD4, CD4/CD8) analyses in MEX-only cohort (though it was not identified as significantly protective in CAM only, possibly as a result of significantly lower power on account of its lower frequency in CAM [AF = 0.009] compared to MEX [AF = 0.02]; p = 0.0004, q < 0.001 [Supplementary Figure S4]). Moreover, B*39:*02*-associated protection was not attributable to co-expression of other advantageous *HLA*-*B* alleles; in fact, a number of B*39:*02*-expressing individuals co-expressed risk alleles including B*35:*01*/*02*/*03*/*12* and B*39:*06*, suggesting that B*39:*02*-associated protective effects may overcome risks associated with deleterious HLA alleles. Consistent with anti-Gag (particularly p24) CD8+ T-cell responses as key determinants of HIV control^61,64–67^, we previously identified two highly significant B*39:*02*-associated polymorphisms in Gag (at codons 315 and 319, both in p24) and three in Pol (at codons 70 and 79 in protease, and 322 in reverse transcriptase)^40^, suggesting strong and reproducible targeting of these regions by B*39:*02*-restricted T-cell responses as possible determinants of B*39:*02*-mediated HIV control, where such effects remain detectable into chronic HIV-1 infection. Interestingly, B*39:*02* has been previously associated with Takayasu’s arteritis, a rare autoimmune disease, in Japanese and Mexican populations^68–73^, and also with spondyloarthropathies in Japan^74^, observations which mirror established links between HLA alleles canonically associated with HIV protection (e.g. B*27:*05*) and autoimmune conditions^75–77^.

In contrast, the common Amerindian B*39:*05* subtype (the 3rd most frequent *HLA*-*B* allele in MEX/CAM [AF = 0.057]) consistently ranked among the strongest risk alleles (score -5 MEX/CAM, -4 MEX and -4 CAM) even after multivariable correction (though it is important to note that this allele is in strong LD with C*07:*02*). Notably, B*39:*05* was not identified as mounting strong immune pressures in Gag p24, but rather in Pol^40^ (at codons 37, 134 and 296). Similarly, no significant Gag codons have been identified as being under strong immune pressure by the Amerindian B*39:*01*, B*39:*06* and B*35:*14* alleles^40^, which were also identified as risk alleles in univariable analyses in the combined MEX/CAM cohort. The common Amerindian B*35:*12* subtype (the 6th most frequent *HLA*-*B* allele in MEX/CAM [AF = 0.042]) was also consistently identified as a risk allele (univariable score -5 in MEX/CAM, -3 in MEX and -3 in CAM; multivariable score -1 in MEX/CAM, -1 in MEX and -2 in CAM). Indeed, by virtue of its similarity with other B*35-Px members, notably B*35:*02*/*03*, at positions 114 and 116 in the HLA peptide binding groove^78^, B*35:*12* represents a putative new member of the established B*35-Px HIV risk allele group^9^.

A number of caveats and considerations merit mention. First, it is important to note the interrelated nature of the five clinical parameters evaluated (e.g. in the MEX/CAM cohort, Spearman rho between pVL and CD4, %CD4 and CD4/CD8 was -0.54 in all cases; Spearman rho between pVL and Z-score was −0.87; Spearman rho for CD4 and Z-score, %CD4 and CD4/CD8 was > 0.79 in all cases). Despite these relatively strong relationships however, pVL and CD4 are nevertheless well-established as independent predictors of HIV progression; as such it is customary to include both in HLA association studies (e.g.^17,19^). Similarly, inclusion of all 5 variables in our scoring system afforded us increased sensitivity to detect both previously-described HLA-HIV associations (e.g. in MEX/CAM univariable: B*57:*02*^17,26^ [score + 2, %CD4 and CD4/CD8], B*44:*03*^5,17,22^ [score + 1, CD4/CD8] and C*08:*01*^20^ [score + 1, %CD4]; in MEX/CAM multivariable: B*42:*01*^5,17,20^ [score + 2, %CD4 and CD4/CD8], B*57:*02*^17,26^ [score + 2, %CD4 and CD4/CD8] and C*08:*01*^20^ [score + 1, CD4/CD8]) as well as novel ones (e.g. in MEX/CAM univariable: B*39:*02* [score + 2, %CD4, CD4/CD8], A*68:*05* [score -2, %CD4 and CD4/CD8], B*35:*14* [score -1, Z-score] and B*40:*01* [score −2, %CD4 and CD4/CD8]). It also allowed us to identify associations with rare HLA alleles (e.g. in MEX/CAM univariable: B*40:*06*^22^ [n = 8, score + 2], B*81:*01*^5,12,17,20^ [n = 16, score + 2], C*14:*03*^22^ [n = 6, score + 1]; in MEX/CAM multivariable: B*57:02^17,26^ [n = 7, score +2] and B*81:01^5,12,17,20^ [n = 16, score -1]). Similarly, the observation that canonical protective associations including B*57:*01*, B*57:*03* and B*27:*05* all achieved the highest protective score (+5) also increases confidence in our identification of B*39:*05* and B*35:*12*, which both achieved the lowest scores (−5), as novel risk alleles. Moreover, the observation that some HLA alleles showed associations with different HIV clinical parameters raises the intriguing possibility that certain HLA subtypes influence HIV progression through different mechanisms (though insufficient power to detect relatively weak associations, combined with the use of a predefined significance threshold, cannot be ruled out). As with all HLA association studies, strong linkage disequilibrium, particularly between *HLA*-*B* and *HLA*-*C* loci located in close proximity within the MHC’s beta block gene group^79–81^ makes it difficult to tease apart individual allele effects in some cases. Associations between certain *HLA*-*C* alleles and HIV clinical parameters may thus be partially or completely attributable to LD with *HLA*-*B* alleles (e.g. B*27:*05* for C*02:*02* and B*35:*12* for C*04:*01*). Moreover, for three *HLA*-*B*~*C* combinations (B*14:*02*~C*08:*02*, B*39:05~C*07:*02* and B*40:01~C*03:*04*), the allele responsible for the observed association could not be resolved due to strong LD. Similarly, we cannot rule out the possibility of additive and/or synergistic effects between HLA alleles. Finally, the risk of spurious associations is an ever-present concern in HLA association studies, particularly when reporting novel associations with relatively rare alleles (e.g. the protective B*15:*39* or the detrimental A*01:*02* alleles, both observed in 8 individuals only). Validation of these novel associations in other cohorts, along with elucidation of possible mechanisms, are therefore warranted. In particular, the evaluation of HIV-specific CTL responses in Latin American individuals expressing protective or risk HLA alleles should provide important mechanistic insights. Despite these caveats, our study further confirms that some HLA allele associations (e.g. B*27:*05* and B*57:*01*) transcend the boundaries of race and HIV subtype, whereas others are likely to be particular to the unique immunogenetic background of the population under study.

Results of our study may also be relevant to the ultimate pursuit of effective HIV vaccines, whether these be prophylactic or therapeutic, global or geographically-tailored. In particular, detailed characterization (and continuous monitoring^82^) of HLA associations with HIV clinical parameters in ethnically diverse human populations hardest hit by the HIV epidemic may help inform the design of CTL epitope-based vaccine constructs^83^, predict the relative population coverage of such constructs, and ultimately aid in the interpretation of results from future HIV vaccine trials in a population-specific context.

## Electronic supplementary material

Supplementary Figures (S1-S4) and Tables (S1-S10)
