# Supplementary material for: Novel HLA class I associations with HIV-1 control in a unique genetically admixed population
Source: Sci Rep. 2018 Apr 17;8:6111. doi: 10.1038/s41598-018-23849-7 (PMC5904102; doi:10.1038/s41598-018-23849-7)
Supplement: Supplementary file 1 — Supplementary Figures (S1-S4) and Tables (S1-S10) [file 41598_2018_23849_MOESM1_ESM.pdf]

Supplementary Figures (S1-S4) and Tables (S1-S10)

Novel HLA class I associations with HIV-1 control in a unique genetically admixed population

Humberto Valenzuela-Ponce, Selma Alva-Hernández, Daniela Garrido-Rodríguez, Maribel Soto-Nava, Thalía García-Téllez, Tania Escamilla-Gómez, Claudia García-Morales, Verónica Sonia Quiroz-Morales, Daniela Tapia-Trejo, Silvia del Arenal, Francisco-Javier Prado-Galbarro, Ramón Hernández-Juan, Edna Rodríguez-Aguirre, Akio Murakami-Ogasawara, Carlos Mejía-Villatoro, Ingrid Y. Escobar-Urias, Rodolfo Pinzón-Meza, Juan Miguel Pascale, Yamitzel Zaldivar, Guillermo Porras-Cortés, Carlos Quant-Durán, Ivette Lorenzana, Rita I. Meza, Elsa Y. Palou, Marvin Manzanero, Rolando A. Cedillos, Carmen Aláez, Mark A. Brockman, P. Richard Harrigan, Chanson J. Brumme, Zabrina L. Brumme, Santiago Ávila-Ríos*, Gustavo Reyes-Terán*, on behalf of the Mesoamerican HIV Project Group.

Supplementary Figures

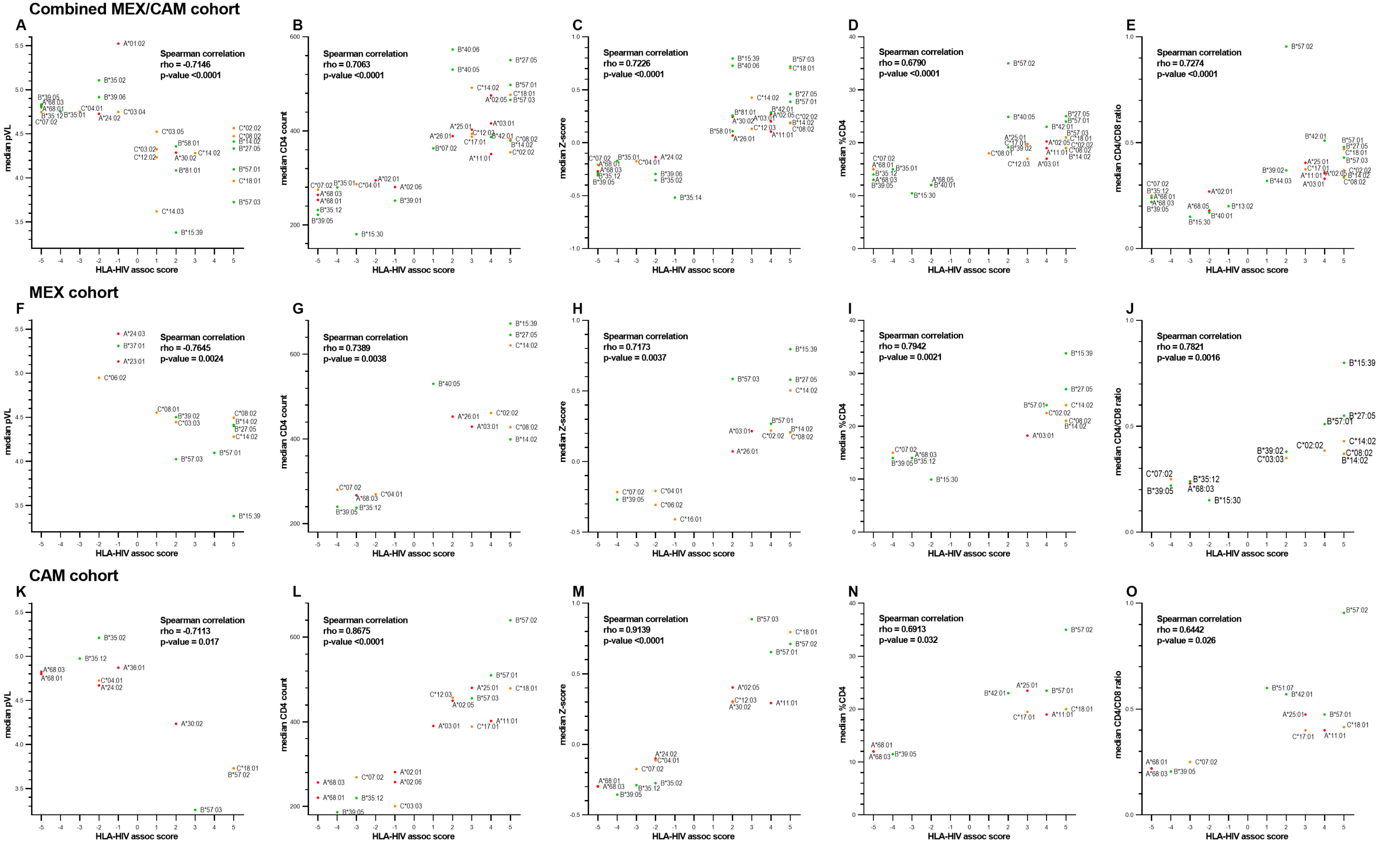

Supplementary Figure S1. Correlation between the 5-parameter HLA-HIV association scores and HIV clinical parameter medians associated to protective/disadvantageous HLA subtypes in the combined MEX/CAM (A-E), MEX (F-J) and CAM (K-O) cohorts. Scatter plot relating 5-parameter HLA-HIV scores to allele-specific pVL (A, F and K), CD4 count (B, G and L), Z-score (C, H and M), %CD4 (D, I and N), and CD4/CD8 ratio (E, J and O) median values. Only HLA alleles identified as significantly protective or risk in at least one Mesoamerican cohort are shown: *HLA-A* subtypes are shown in red, *HLA-B* in green and *HLA-C* in orange dots. Correlations were determined using Spearman's rank test.

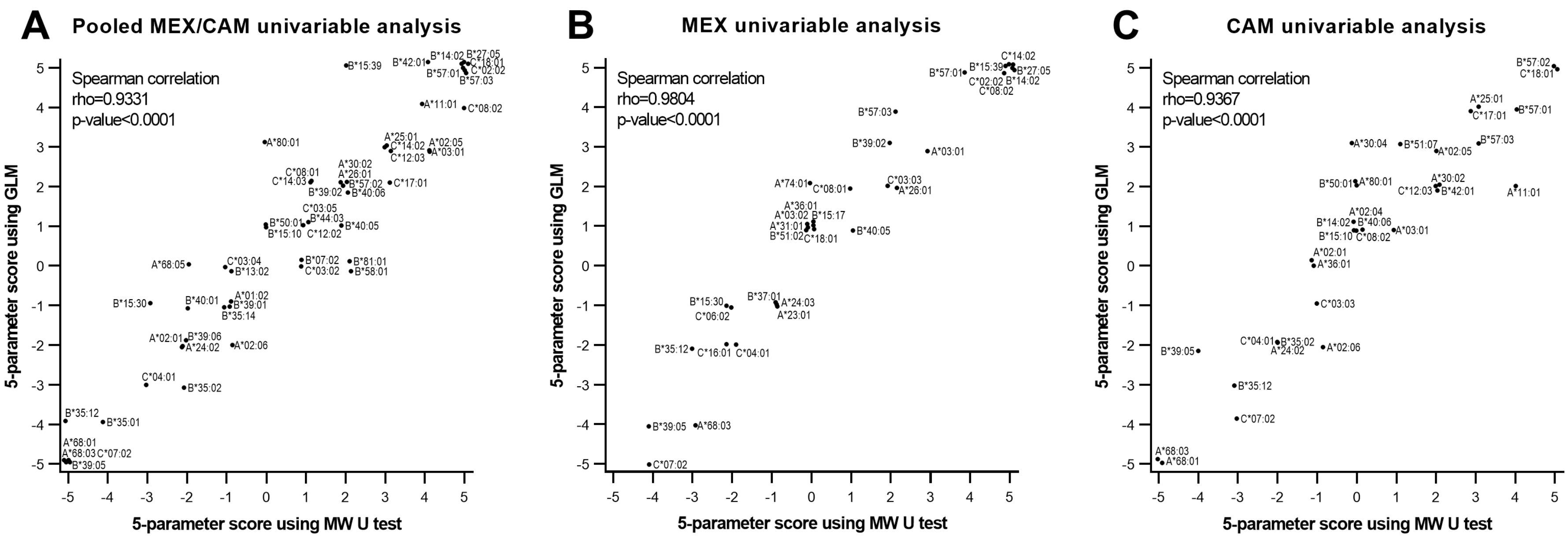

Supplementary Figure S2. Correlation between the 5-parameter HLA-HIV scores attained with Mann-Whitney U tests and linear regression (GLM). Scatter plot relating 5-parameter HLA-HIV scores obtained in univariable analysis using Mann-Whitney U tests and linear regression (Generalized Linear Model). Correlations were determined using Spearman's rank test. Only HLA alleles with at least one significant association were used.

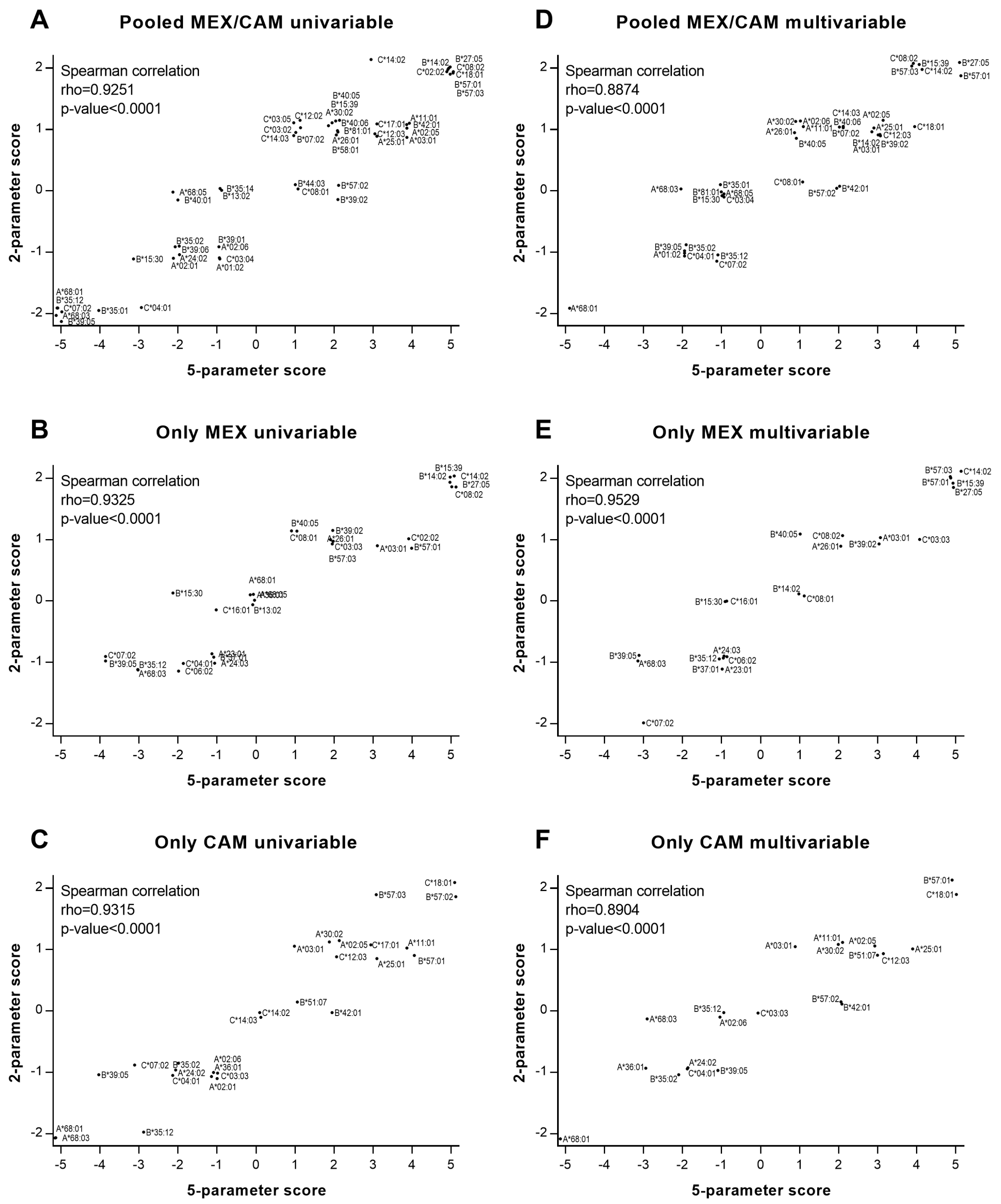

Supplementary Figure S3. Correlation between the 5-parameter (pVL, CD4, Z-score, %CD4, CD4/CD8) and 2-parameter (based only in pVL and CD4) scoring system in univariable and multivariable analyses. Scatter plot relating the 5-parameter and the 2-parameter scoring system based only in pVL and CD4 in univariable (A-C) and multivariable (D-F) in the combined MEX/CAM (A and D), MEX (B and E) and CAM (C and F) cohorts. Only HLA alleles identified to be associated with at least one clinical parameter were taken into account. Correlations were determined using Spearman's rank test.

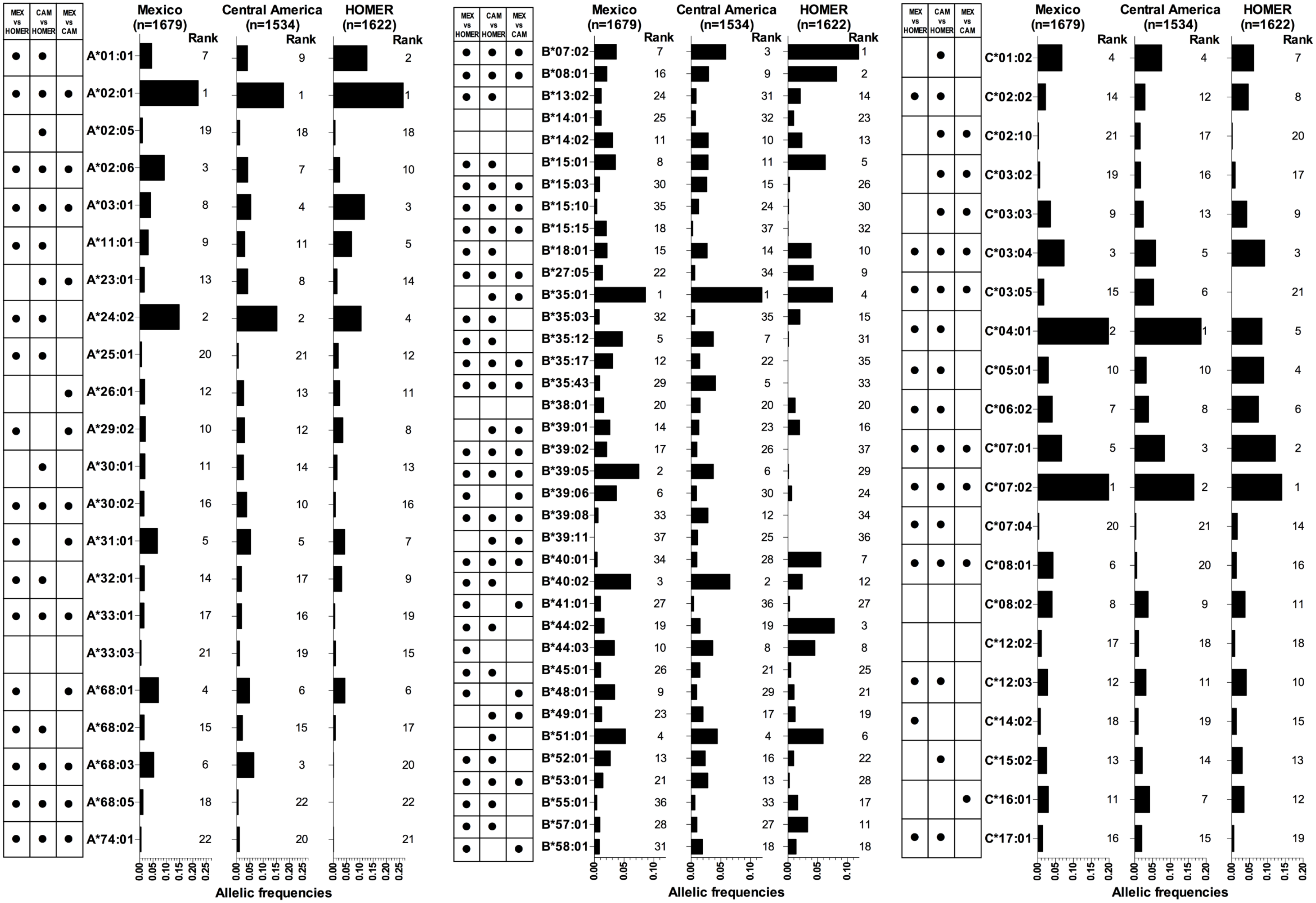

Supplementary Figure S4. Comparison of HLA class I allele frequencies between MEX (n=1679), CAM (n=1534), and HOMER (n=1622). HLA allele frequencies (AF>0.001) were compared using Fisher's exact test, with multiple comparisons addressed using q-values. Significant differences (p<0.05, q<0.2) are denoted by (●).

Supplementary Table S1. List of HLA gametic phase ambiguities.

HLA-A **ambiguity**	**Frequency**	**Percent**
*A*02:01+A*68:01/A*02:22+A*68:08*	80	56.34
*A*02:01+A*03:01/A*02:24+A*03:17*	56	39.44
*A*02:01+A*66:01/A*02:35+A*26:03*	4	2.82
*A*02:01+A*31:01/A*02:20+A*31:02*	1	0.7
*A*26:03+A*68:01/A*66:01+A*68:05*	1	0.7
**Total**	**142**	**100**
HLA-B **ambiguity**	**Frequency**	**Percent**
*B*15:01+B*35:01/B*15:05+B*35:14*	21	13.04
*B*35:01+B*51:01/B*35:11+B*51:09/B*35:24+B*51:02/B*53:01+B*78:02*	20	12.42
*B*35:01+B*38:01/B*39:05+B*53:01*	15	9.32
*B*15:01+B*40:02/B*15:07+B*40:11*	14	8.7
*B*38:01+B*78:02/B*39:05+B*51:01*	13	8.07
*B*35:01+B*35:43/B*35:14+B*35:32*	12	7.45
*B*39:01+B*39:13/B*39:02+B*39:05*	10	6.21
*B*15:01+B*51:01/B*15:38+B*51:02*	9	5.59
*B*35:01+B*39:01/B*35:29+B*39:05*	9	5.59
*B*35:01+B*49:01/B*50:01+B*53:01*	6	3.73
*B*38:01+B*50:01/B*39:05+B*49:01*	4	2.48
*B*15:01+B*48:01/B*15:07+B*48:03*	3	1.86
*B*35:01+B*55:01/B*35:11+B*55:02*	3	1.86
*B*15:01+B*39:03/B*15:07+B*39:01*	2	1.24
*B*15:01+B*39:39/B*15:15+B*39:02*	2	1.24
*B*35:29+B*38:01/B*39:01+B*53:01*	2	1.24
*B*38:01+B*78:01/B*39:01+B*51:01/B*39:06+B*51:06*	2	1.24
*B*07:02+B*15:09/B*07:05+B*15:10*	1	0.62
*B*15:01+B*35:04/B*15:30+B*35:01*	1	0.62
*B*15:01+B*35:11/B*15:39+B*35:14*	1	0.62
*B*15:01+B*41:02/B*15:07+B*41:03*	1	0.62
*B*15:02+B*52:01/B*15:25+B*51:07*	1	0.62
*B*15:02+B*53:01/B*15:13+B*35:01*	1	0.62
*B*15:03+B*39:01/B*15:18+B*39:02*	1	0.62
*B*18:01+B*35:29/B*18:03+B*35:01*	1	0.62
*B*35:01+B*39:11/B*35:08+B*39:05*	1	0.62
*B*35:01+B*44:18/B*45:01+B*53:01*	1	0.62
*B*35:01+B*57:03/B*35:04+B*57:01*	1	0.62
*B*39:01+B*78:02/B*39:05+B*78:01*	1	0.62
*B*40:01+B*42:01/B*41:03+B*81:01*	1	0.62
*B*51:01+B*53:17/B*52:01+B*53:01*	1	0.62
**Total**	**161**	**100**
HLA-C **ambiguity**	**Frequency**	**Percent**
*C*03:03+C*07:02/C*03:04+C*07:56*	33	31.73
*C*02:02+C*04:01/C*02:10+C*04:01*	14	13.46
*C*05:01+C*16:01/C*08:02+C*16:02*	10	9.62
*C*02:02+C*07:01/C*02:02+C*07:01/C*02:10+C*07:01*	8	7.69
*C*05:01+C*12:03/C*08:02+C*12:04*	7	6.73
*C*02:02+C*06:02/C*02:10+C*06:02*	5	4.81
*C*02:02+C*07:02/C*02:10+C*07:02*	5	4.81
*C*01:02+C*02:10/C*01:02+C*02:02*	3	2.88
*C*02:02+C*03:04/C*02:10+C*03:04*	3	2.88
*C*03:04+C*15:09/C*03:40+C*15:06*	3	2.88
*C*02:02+C*03:02/C*02:10+C*03:02*	2	1.92
*C*02:02+C*03:03/C*02:10+C*03:03*	2	1.92
*C*02:02+C*05:01/C*02:10+C*05:01*	2	1.92
*C*02:02+C*02:02/C*02:02+C*02:10*	1	0.96
*C*02:02+C*12:02/C*02:10+C*12:02*	1	0.96
*C*02:02+C*14:02/C*02:10+C*14:02*	1	0.96
*C*02:02+C*15:05/C*02:10+C*15:05*	1	0.96
*C*02:02+C*17:01/C*02:10+C*17:01*	1	0.96
*C*04:01+C*08:04/C*04:04+C*08:02*	1	0.96
*C*12:03+C*16:02/C*12:04+C*16:01*	1	0.96
**Total**	**104**	**100**

Supplementary Table S2. HLA 2-loci linkage disequilibria in the MEX cohort. Table headers: Both, number of individuals expressing both HLA1 and HLA2. Only HLA1, number of individuals expressing HLA1, but not HLA2. Only HLA2, number of individuals expressing HLA2, but not HLA1. Neither, number of individuals expressing neither HLA1 nor HLA2. P(HLA1), observed frequency of individuals with HLA1 in the population. P(HLA2), observed frequency of individuals with HLA2 in the population. P(HLA1 & HLA2), observed frequency of individuals with both HLA1 and HLA2 in the population. E(P(HLA1 & HLA2)), expected frequency of P(HLA1 & HLA2) if P(HLA1) and P(HLA2) are independent [E(P(HLA1 & HLA2)) = P(HLA1) * P(HLA2)]. P-value, two-sided Fisher's exact p-value. This is an estimate of the probability that P(HLA1) and P(HLA2) are independent, given the observed data. A small p-value indicates that the occurrence of HLA1 and HLA2 is linked in the population. The contingency matrix is [[both, only hla1],[only hla2],[neither]]. Multiple comparisons were addressed by Bonferroni correction. Based on populations statistics: number of distinct two-way tests performed=N2=12970. P-value for two-way comparisons = 0.05/N2 = 3.855e-06 for a 95% confidence level. Displaying results with p-values less than 3.855e-06.

HLA1	HLA2	Population Frequency	p-value	Both	Only HLA1	Only HLA2	Neither	P(HLA1)	P(HLA2)	P(HLA1 & HLA2)	E(P(HLA1 & HLA2))
*B*39:05*	*C*07:02*	0.1303	4.6E-97	223	12	383	1094	0.1373	0.354	0.1303	0.0486
*B*35:01*	*C*04:01*	0.1221	4.8E-57	209	57	390	1056	0.1554	0.3499	0.1221	0.0544
*B*35:12*	*C*04:01*	0.0835	9.2E-60	143	8	456	1105	0.0882	0.3499	0.0835	0.0309
*A*68:03*	*C*07:02*	0.0783	8.7E-32	134	41	472	1065	0.1022	0.354	0.0783	0.0362
*B*07:02*	*C*07:02*	0.0643	6.7E-41	110	10	496	1096	0.0701	0.354	0.0643	0.0248
*B*39:06*	*C*07:02*	0.0625	2.1E-43	107	6	499	1100	0.066	0.354	0.0625	0.0234
*A*68:03*	*B*39:05*	0.0596	5.2E-51	102	73	133	1404	0.1022	0.1373	0.0596	0.014
*B*48:01*	*C*08:01*	0.0572	1.4E-107	98	13	47	1554	0.0648	0.0847	0.0572	0.0055
*A*02:01*	*B*35:12*	0.0561	1.4E-10	96	568	55	993	0.3879	0.0882	0.0561	0.0342
*B*35:17*	*C*04:01*	0.0555	2.2E-40	95	4	504	1109	0.0578	0.3499	0.0555	0.0202
*B*40:02*	*C*03:04*	0.0555	1.1E-36	95	102	143	1372	0.1151	0.139	0.0555	0.016
*B*14:02*	*C*08:02*	0.0520	2.9E-101	89	11	45	1567	0.0584	0.0783	0.052	0.0046
*A*24:02*	*B*39:06*	0.0467	5.4E-23	80	394	33	1205	0.2769	0.066	0.0467	0.0183
*A*02:06*	*B*39:05*	0.0415	1.5E-07	71	227	164	1250	0.1741	0.1373	0.0415	0.0239
*A*68:01*	*C*03:04*	0.0403	5.3E-12	69	161	169	1313	0.1343	0.139	0.0403	0.0187
*B*15:01*	*C*01:02*	0.0386	4.3E-34	66	47	150	1449	0.066	0.1262	0.0386	0.0083
*B*39:01*	*C*07:02*	0.0386	1.6E-16	66	18	540	1088	0.0491	0.354	0.0386	0.0174
*B*44:03*	*C*16:01*	0.0362	2.7E-58	62	46	35	1569	0.0631	0.0567	0.0362	0.0036
*B*15:15*	*C*01:02*	0.0356	2.0E-51	61	6	155	1490	0.0391	0.1262	0.0356	0.0049
*B*08:01*	*C*07:01*	0.0350	4.5E-50	60	7	152	1493	0.0391	0.1238	0.035	0.0048
*B*51:01*	*C*15:02*	0.0333	4.9E-43	57	107	25	1523	0.0958	0.0479	0.0333	0.0046
*A*01:01*	*C*07:01*	0.0327	5.7E-17	56	93	156	1407	0.087	0.1238	0.0327	0.0108
*B*39:02*	*C*07:02*	0.0327	7.5E-15	56	14	550	1092	0.0409	0.354	0.0327	0.0145
*A*02:01*	*B*15:15*	0.0310	1.0E-11	53	611	14	1034	0.3879	0.0391	0.031	0.0152
*B*52:01*	*C*03:03*	0.0280	2.6E-37	48	37	71	1556	0.0496	0.0695	0.028	0.0035
*B*44:02*	*C*05:01*	0.0263	3.8E-51	45	11	51	1605	0.0327	0.0561	0.0263	0.0018
*A*29:02*	*C*16:01*	0.0251	1.2E-39	43	28	54	1587	0.0415	0.0567	0.0251	0.0023
*B*40:02*	*C*03:05*	0.0251	1.2E-29	43	154	16	1499	0.1151	0.0345	0.0251	0.004
*A*33:01*	*C*08:02*	0.0239	1.5E-36	41	14	93	1564	0.0321	0.0783	0.0239	0.0025
*A*29:02*	*B*44:03*	0.0239	4.5E-34	41	30	67	1574	0.0415	0.0631	0.0239	0.0026
*B*13:02*	*C*06:02*	0.0222	7.9E-45	38	0	98	1576	0.0222	0.0794	0.0222	0.0018
*B*38:01*	*C*12:03*	0.0222	7.3E-41	38	14	53	1607	0.0304	0.0532	0.0222	0.0016
*A*33:01*	*B*14:02*	0.0216	1.2E-35	37	18	63	1594	0.0321	0.0584	0.0216	0.0019
*A*03:01*	*B*07:02*	0.0216	1.1E-14	37	99	83	1493	0.0794	0.0701	0.0216	0.0056
*B*49:01*	*C*07:01*	0.0210	8.1E-30	36	4	176	1496	0.0234	0.1238	0.021	0.0029
*B*53:01*	*C*04:01*	0.0210	5.4E-09	36	11	563	1102	0.0275	0.3499	0.021	0.0096
*B*15:01*	*C*03:03*	0.0204	5.7E-16	35	78	84	1515	0.066	0.0695	0.0204	0.0046
*A*24:02*	*C*03:05*	0.0199	9.4E-07	34	440	25	1213	0.2769	0.0345	0.0199	0.0095
*A*30:01*	*C*06:02*	0.0187	2.4E-19	32	35	104	1541	0.0391	0.0794	0.0187	0.0031
*A*01:01*	*C*06:02*	0.0187	3.3E-08	32	117	104	1459	0.087	0.0794	0.0187	0.0069
*B*14:01*	*C*08:02*	0.0181	5.8E-29	31	8	103	1570	0.0228	0.0783	0.0181	0.0018
*B*35:02*	*C*04:01*	0.0181	4.3E-15	31	0	568	1113	0.0181	0.3499	0.0181	0.0063
*B*35:14*	*C*04:01*	0.0175	2.9E-12	30	2	569	1111	0.0187	0.3499	0.0175	0.0065
*B*27:05*	*C*02:02*	0.0169	3.4E-32	29	15	43	1625	0.0257	0.0421	0.0169	0.0011
*B*35:43*	*C*01:02*	0.0164	3.5E-25	28	1	188	1495	0.0169	0.1262	0.0164	0.0021
*B*18:01*	*C*05:01*	0.0164	2.9E-19	28	40	68	1576	0.0397	0.0561	0.0164	0.0022
*A*01:01*	*B*08:01*	0.0164	5.9E-14	28	121	39	1524	0.087	0.0391	0.0164	0.0034

*B*52:01*	*C*12:02*	0.0158	1.3E-30	27	58	8	1619	0.0496	0.0204	0.0158	0.001
*B*18:01*	*C*12:03*	0.0152	1.5E-17	26	42	65	1579	0.0397	0.0532	0.0152	0.0021
*A*30:01*	*B*13:02*	0.0146	1.8E-28	25	42	13	1632	0.0391	0.0222	0.0146	0.0009
*B*40:02*	*C*03:06*	0.0140	1.7E-20	24	173	3	1512	0.1151	0.0158	0.014	0.0018
*A*02:05*	*C*07:01*	0.0140	6.2E-15	24	11	188	1489	0.0204	0.1238	0.014	0.0025
*B*51:01*	*C*15:09*	0.0134	1.1E-20	23	141	4	1544	0.0958	0.0158	0.0134	0.0015
*B*51:01*	*C*14:02*	0.0123	5.2E-18	21	143	5	1543	0.0958	0.0152	0.0123	0.0015
*A*01:01*	*B*57:01*	0.0123	2.9E-16	21	128	10	1553	0.087	0.0181	0.0123	0.0016
*A*26:01*	*C*12:03*	0.0123	1.1E-12	21	43	70	1578	0.0374	0.0532	0.0123	0.002
*A*30:02*	*C*05:01*	0.0117	9.0E-13	20	34	76	1582	0.0315	0.0561	0.0117	0.0018
*B*39:08*	*C*07:02*	0.0117	1.1E-08	20	1	586	1105	0.0123	0.354	0.0117	0.0043
*B*41:01*	*C*07:01*	0.0111	1.8E-09	19	16	193	1484	0.0204	0.1238	0.0111	0.0025
*A*26:01*	*B*38:01*	0.0105	2.8E-14	18	46	34	1614	0.0374	0.0304	0.0105	0.0011
*B*45:01*	*C*06:02*	0.0105	1.4E-11	18	18	118	1558	0.021	0.0794	0.0105	0.0017
*B*40:08*	*C*03:04*	0.0099	1.3E-12	17	3	221	1471	0.0117	0.139	0.0099	0.0016
*B*57:01*	*C*06:02*	0.0099	7.7E-12	17	14	119	1562	0.0181	0.0794	0.0099	0.0014
*B*41:01*	*C*17:01*	0.0093	2.7E-17	16	19	32	1645	0.0204	0.028	0.0093	0.0006
*B*15:30*	*C*01:02*	0.0093	3.8E-14	16	1	200	1495	0.0099	0.1262	0.0093	0.0013
*B*40:05*	*C*03:04*	0.0093	1.5E-12	16	2	222	1472	0.0105	0.139	0.0093	0.0015
*A*30:01*	*C*17:01*	0.0093	4.1E-12	16	51	32	1613	0.0391	0.028	0.0093	0.0011
*B*58:01*	*C*07:01*	0.0093	1.4E-08	16	12	196	1488	0.0164	0.1238	0.0093	0.002
*B*44:03*	*C*07:02*	0.0093	1.2E-06	16	92	590	1014	0.0631	0.354	0.0093	0.0223
*A*68:02*	*C*08:02*	0.0093	3.0E-06	16	41	118	1537	0.0333	0.0783	0.0093	0.0026
*B*42:01*	*C*17:01*	0.0088	4.8E-25	15	0	33	1664	0.0088	0.028	0.0088	0.0002
*B*51:02*	*C*08:01*	0.0088	4.9E-15	15	2	130	1565	0.0099	0.0847	0.0088	0.0008
*A*30:02*	*B*18:01*	0.0088	5.2E-10	15	39	53	1605	0.0315	0.0397	0.0088	0.0013
*B*15:01*	*C*07:02*	0.0088	7.1E-08	15	98	591	1008	0.066	0.354	0.0088	0.0234
*B*35:16*	*C*04:01*	0.0088	1.3E-07	15	0	584	1113	0.0088	0.3499	0.0088	0.0031
*B*57:01*	*C*07:01*	0.0088	7.5E-07	15	16	197	1484	0.0181	0.1238	0.0088	0.0022
*A*02:05*	*B*41:01*	0.0082	2.1E-16	14	21	21	1656	0.0204	0.0204	0.0082	0.0004
*B*50:01*	*C*06:02*	0.0082	3.9E-11	14	8	122	1568	0.0129	0.0794	0.0082	0.001
*B*45:01*	*C*16:01*	0.0082	1.9E-09	14	22	83	1593	0.021	0.0567	0.0082	0.0012
*A*68:02*	*B*14:02*	0.0082	2.1E-06	14	43	86	1569	0.0333	0.0584	0.0082	0.0019
*B*40:01*	*C*03:04*	0.0076	2.1E-09	13	3	225	1471	0.0093	0.139	0.0076	0.0013
*A*25:01*	*C*12:03*	0.0070	4.0E-10	12	12	79	1609	0.014	0.0532	0.007	0.0007
*A*11:01*	*C*12:02*	0.0070	5.3E-07	12	96	23	1581	0.0631	0.0204	0.007	0.0013
*B*37:01*	*C*06:02*	0.0064	5.4E-13	11	0	125	1576	0.0064	0.0794	0.0064	0.0005
*B*55:01*	*C*03:03*	0.0064	3.6E-11	11	3	108	1590	0.0082	0.0695	0.0064	0.0006
*B*15:03*	*C*02:02*	0.0064	2.6E-09	11	16	61	1624	0.0158	0.0421	0.0064	0.0007
*B*15:10*	*C*03:04*	0.0064	7.5E-08	11	3	227	1471	0.0082	0.139	0.0064	0.0011
*B*58:01*	*C*03:02*	0.0058	7.2E-14	10	18	11	1673	0.0164	0.0123	0.0058	0.0002
*A*30:01*	*B*42:01*	0.0058	1.1E-11	10	57	5	1640	0.0391	0.0088	0.0058	0.0003
*B*56:01*	*C*01:02*	0.0058	4.4E-08	10	2	206	1494	0.007	0.1262	0.0058	0.0009
*B*58:02*	*C*06:02*	0.0053	9.8E-11	9	0	127	1576	0.0053	0.0794	0.0053	0.0004
*B*15:17*	*C*07:01*	0.0053	5.9E-09	9	0	203	1500	0.0053	0.1238	0.0053	0.0007
*B*35:01*	*C*07:01*	0.0053	8.0E-08	9	257	203	1243	0.1554	0.1238	0.0053	0.0192
*A*25:01*	*B*18:01*	0.0053	1.2E-07	9	15	59	1629	0.014	0.0397	0.0053	0.0006
*B*07:05*	*C*15:05*	0.0047	8.4E-14	8	9	7	1688	0.0099	0.0088	0.0047	0.0001
*B*48:01*	*C*08:03*	0.0047	2.5E-10	8	103	0	1601	0.0648	0.0047	0.0047	0.0003
*A*23:01*	*B*15:03*	0.0047	1.6E-06	8	51	19	1634	0.0345	0.0158	0.0047	0.0005
*B*15:03*	*C*02:10*	0.0041	1.2E-11	7	20	3	1682	0.0158	0.0058	0.0041	0.0001
*B*57:03*	*C*18:01*	0.0035	1.9E-11	6	10	3	1693	0.0093	0.0053	0.0035	0
*B*41:02*	*C*17:01*	0.0035	9.5E-09	6	2	42	1662	0.0047	0.028	0.0035	0.0001
*A*74:01*	*B*15:03*	0.0029	2.6E-06	5	11	22	1674	0.0093	0.0158	0.0029	0.0001
*B*15:18*	*C*07:04*	0.0018	2.6E-07	3	0	9	1700	0.0018	0.007	0.0018	0
*A*02:11*	*B*48:02*	0.0012	2.0E-06	2	1	0	1709	0.0018	0.0012	0.0012	0
*B*14:01*	*C*04:01*	0.0006	1.4E-06	1	38	598	1075	0.0228	0.3499	0.0006	0.008

Supplementary Table S3. HLA 3-loci linkage disequilibria in the MEX cohort. For each statistically significant HLA pair identified in Supplementary Table S2, Fisher's exact test is used to assess linkage with a third gene. Multiple comparisons were addressed by Bonferroni correction. For each of the statistically-significant HLA pair discovered by the two-way comparison above, a Fisher's test is performed against the third gene. Number of distinct three-way Fisher's tests performed = N3 = 6251. P-value for three-way comparisons = 0.05/N3 = 7.999e-06 for a 95% confidence level.

HLA1	HLA2	HLA3	Population Frequency	p-value	All	Only HLA1&HLA2	Only HLA3	None	P(HLA1 & HLA2)	P(HLA3)	P(HLA1 & HLA2 & HLA3)	E(P(HLA1 & HLA2 & HLA3))
*A*68:03*	*B*39:05*	*C*07:02*	0.0783	8.7E-32	134	41	472	1065	0.1022	0.354	0.0783	0.0362
*B*35:12*	*C*04:01*	*A*02:01*	0.0561	1.4E-10	96	55	568	993	0.0882	0.3879	0.0561	0.0342
*A*24:02*	*C*03:05*	*B*39:06*	0.0467	5.4E-23	80	394	33	1205	0.2769	0.066	0.0467	0.0183
*B*39:06*	*C*07:02*	*A*24:02*	0.0467	5.4E-23	80	33	394	1205	0.066	0.2769	0.0467	0.0183
*B*39:05*	*C*07:02*	*A*02:06*	0.0415	1.5E-07	71	164	227	1250	0.1373	0.1741	0.0415	0.0239
*A*01:01*	*B*08:01*	*C*07:01*	0.0327	5.7E-17	56	93	156	1407	0.087	0.1238	0.0327	0.0108
*A*01:01*	*B*57:01*	*C*07:01*	0.0327	5.7E-17	56	93	156	1407	0.087	0.1238	0.0327	0.0108
*B*15:15*	*C*01:02*	*A*02:01*	0.0310	1.0E-11	53	14	611	1034	0.0391	0.3879	0.031	0.0152
*A*29:02*	*B*44:03*	*C*16:01*	0.0251	1.2E-39	43	28	54	1587	0.0415	0.0567	0.0251	0.0023
*B*44:03*	*C*07:02*	*A*29:02*	0.0239	4.5E-34	41	67	30	1574	0.0631	0.0415	0.0239	0.0026
*A*33:01*	*B*14:02*	*C*08:02*	0.0239	1.5E-36	41	14	93	1564	0.0321	0.0783	0.0239	0.0025
*B*07:02*	*C*07:02*	*A*03:01*	0.0216	1.1E-14	37	83	99	1493	0.0701	0.0794	0.0216	0.0056
*A*01:01*	*B*08:01*	*C*06:02*	0.0187	3.3E-08	32	117	104	1459	0.087	0.0794	0.0187	0.0069
*A*01:01*	*B*57:01*	*C*06:02*	0.0187	3.3E-08	32	117	104	1459	0.087	0.0794	0.0187	0.0069
*A*30:01*	*B*13:02*	*C*06:02*	0.0187	2.4E-19	32	35	104	1541	0.0391	0.0794	0.0187	0.0031
*A*30:01*	*B*42:01*	*C*06:02*	0.0187	2.4E-19	32	35	104	1541	0.0391	0.0794	0.0187	0.0031
*A*01:01*	*B*08:01*	*C*07:02*	0.0164	5.9E-06	28	121	578	985	0.087	0.354	0.0164	0.0308
*A*01:01*	*B*57:01*	*C*07:02*	0.0164	5.9E-06	28	121	578	985	0.087	0.354	0.0164	0.0308
*A*30:01*	*C*17:01*	*B*13:02*	0.0146	1.8E-28	25	42	13	1632	0.0391	0.0222	0.0146	0.0009
*A*02:05*	*B*41:01*	*C*07:01*	0.0140	6.2E-15	24	11	188	1489	0.0204	0.1238	0.014	0.0025
*A*26:01*	*B*38:01*	*C*12:03*	0.0123	1.1E-12	21	43	70	1578	0.0374	0.0532	0.0123	0.002
*A*30:02*	*B*18:01*	*C*05:01*	0.0117	9.0E-13	20	34	76	1582	0.0315	0.0561	0.0117	0.0018
*A*23:01*	*B*15:03*	*C*06:02*	0.0093	6.0E-06	16	43	120	1533	0.0345	0.0794	0.0093	0.0027
*A*30:01*	*B*42:01*	*C*17:01*	0.0093	4.1E-12	16	51	32	1613	0.0391	0.028	0.0093	0.0011
*A*68:02*	*B*14:02*	*C*08:02*	0.0093	3.0E-06	16	41	118	1537	0.0333	0.0783	0.0093	0.0026
*B*18:01*	*C*12:03*	*A*30:02*	0.0088	5.2E-10	15	53	39	1605	0.0397	0.0315	0.0088	0.0013
*B*41:01*	*C*17:01*	*A*02:05*	0.0082	2.1E-16	14	21	21	1656	0.0204	0.0204	0.0082	0.0004
*A*25:01*	*B*18:01*	*C*12:03*	0.0070	4.0E-10	12	12	79	1609	0.014	0.0532	0.007	0.0007
*B*18:01*	*C*05:01*	*A*25:01*	0.0053	1.2E-07	9	59	15	1629	0.0397	0.014	0.0053	0.0006
*B*14:01*	*C*04:01*	*A*26:01*	0.0053	7.1E-06	9	30	55	1618	0.0228	0.0374	0.0053	0.0009
*B*14:01*	*C*08:02*	*A*26:01*	0.0053	7.1E-06	9	30	55	1618	0.0228	0.0374	0.0053	0.0009
*A*26:01*	*C*12:03*	*B*14:01*	0.0053	7.1E-06	9	55	30	1618	0.0374	0.0228	0.0053	0.0009
*B*15:03*	*C*02:02*	*A*23:01*	0.0047	1.6E-06	8	19	51	1634	0.0158	0.0345	0.0047	0.0005
*B*15:03*	*C*02:10*	*A*23:01*	0.0047	1.6E-06	8	19	51	1634	0.0158	0.0345	0.0047	0.0005
*B*15:03*	*C*02:02*	*A*74:01*	0.0029	2.6E-06	5	22	11	1674	0.0158	0.0093	0.0029	0.0001
*B*15:03*	*C*02:10*	*A*74:01*	0.0029	2.6E-06	5	22	11	1674	0.0158	0.0093	0.0029	0.0001

Supplementary Table S4. HLA 2-loci linkage disequilibria in the CAM cohort. Table headers same as Supplementary Table S2. Multiple comparisons were addressed by Bonferroni correction. Based on population statistics. Number of distinct two-way tests performed = N2 = 14234. P-value for two-way comparisons = 0.05/N2 = 3.513e-06 for a 95% confidence level.

HLA1	HLA2	Population Frequency	p-value	Both	Only HLA1	Only HLA2	Neither	P(HLA1)	P(HLA2)	P(HLA1 & HLA2)	E(P(HLA1 & HLA2))
*B*35:01*	*C*04:01*	0.1374	6.7E-44	211	105	297	923	0.2057	0.3307	0.1374	0.068
*B*07:02*	*C*07:02*	0.0853	5.7E-54	131	24	305	1076	0.1009	0.2839	0.0853	0.0286
*B*40:02*	*C*03:05*	0.0710	3.2E-80	109	75	43	1309	0.1198	0.099	0.071	0.0119
*B*35:43*	*C*01:02*	0.0671	7.0E-92	103	5	113	1315	0.0703	0.1406	0.0671	0.0099
*A*24:02*	*B*40:02*	0.0671	5.1E-18	103	322	81	1030	0.2767	0.1198	0.0671	0.0331
*B*35:12*	*C*04:01*	0.0658	3.8E-45	101	5	407	1023	0.069	0.3307	0.0658	0.0228
*A*68:03*	*C*07:02*	0.0638	3.1E-14	98	86	338	1014	0.1198	0.2839	0.0638	0.034
*A*24:02*	*C*03:05*	0.0625	4.2E-22	96	329	56	1055	0.2767	0.099	0.0625	0.0274
*B*39:05*	*C*07:02*	0.0612	3.6E-45	94	8	342	1092	0.0664	0.2839	0.0612	0.0188
*A*24:02*	*C*01:02*	0.0592	7.2E-07	91	334	125	986	0.2767	0.1406	0.0592	0.0389
*B*14:02*	*C*08:02*	0.0501	6.6E-95	77	7	32	1420	0.0547	0.071	0.0501	0.0039
*B*39:08*	*C*07:02*	0.0482	7.4E-33	74	9	362	1091	0.054	0.2839	0.0482	0.0153
*A*02:01*	*B*35:12*	0.0469	5.4E-15	72	422	34	1008	0.3216	0.069	0.0469	0.0222
*B*08:01*	*C*07:01*	0.0449	8.6E-43	69	18	173	1276	0.0566	0.1576	0.0449	0.0089
*A*02:06*	*C*07:02*	0.0449	7.0E-11	69	56	367	1044	0.0814	0.2839	0.0449	0.0231
*A*01:01*	*C*07:01*	0.0417	4.8E-24	64	55	178	1239	0.0775	0.1576	0.0417	0.0122
*B*53:01*	*C*04:01*	0.0397	5.6E-17	61	17	447	1011	0.0508	0.3307	0.0397	0.0168
*B*40:02*	*C*03:04*	0.0365	3.4E-15	56	128	112	1240	0.1198	0.1094	0.0365	0.0131
*B*49:01*	*C*07:01*	0.0339	1.0E-35	52	8	190	1286	0.0391	0.1576	0.0339	0.0062
*A*68:03*	*B*39:05*	0.0326	3.5E-22	50	134	52	1300	0.1198	0.0664	0.0326	0.008
*B*15:01*	*C*01:02*	0.0319	7.4E-25	49	29	167	1291	0.0508	0.1406	0.0319	0.0071
*A*03:01*	*B*07:02*	0.0319	2.9E-15	49	108	106	1273	0.1022	0.1009	0.0319	0.0103
*A*29:02*	*C*16:01*	0.0299	2.3E-30	46	43	77	1370	0.0579	0.0801	0.0299	0.0046
*B*44:03*	*C*16:01*	0.0299	4.6E-26	46	60	77	1353	0.069	0.0801	0.0299	0.0055
*A*01:01*	*B*08:01*	0.0286	3.8E-29	44	75	43	1374	0.0775	0.0566	0.0286	0.0044
*B*15:03*	*C*02:10*	0.0273	9.1E-58	42	30	4	1460	0.0469	0.0299	0.0273	0.0014
*B*51:01*	*C*15:02*	0.0267	1.4E-33	41	81	20	1394	0.0794	0.0397	0.0267	0.0032
*A*29:02*	*B*44:03*	0.0260	4.6E-26	40	49	66	1381	0.0579	0.069	0.026	0.004
*B*44:02*	*C*05:01*	0.0247	1.9E-42	38	7	58	1433	0.0293	0.0625	0.0247	0.0018
*B*38:01*	*C*12:03*	0.0247	6.4E-42	38	8	57	1433	0.0299	0.0618	0.0247	0.0019
*B*35:17*	*C*04:01*	0.0234	4.5E-14	36	4	472	1024	0.026	0.3307	0.0234	0.0086
*B*18:01*	*C*05:01*	0.0208	2.1E-20	32	47	64	1393	0.0514	0.0625	0.0208	0.0032
*B*39:11*	*C*07:02*	0.0208	1.4E-18	32	0	404	1100	0.0208	0.2839	0.0208	0.0059
*A*02:06*	*B*39:08*	0.0202	8.6E-15	31	94	52	1359	0.0814	0.054	0.0202	0.0044
*A*68:03*	*B*35:43*	0.0195	3.3E-06	30	154	78	1274	0.1198	0.0703	0.0195	0.0084
*A*30:01*	*C*17:01*	0.0189	3.3E-24	29	50	30	1427	0.0514	0.0384	0.0189	0.002
*B*58:01*	*C*07:01*	0.0182	1.0E-09	28	28	214	1266	0.0365	0.1576	0.0182	0.0057
*B*52:01*	*C*12:02*	0.0176	1.9E-35	27	37	5	1467	0.0417	0.0208	0.0176	0.0009
*A*33:01*	*C*08:02*	0.0176	9.8E-18	27	30	82	1397	0.0371	0.071	0.0176	0.0026
*A*33:01*	*B*14:02*	0.0163	1.8E-18	25	32	59	1420	0.0371	0.0547	0.0163	0.002
*B*15:03*	*C*02:02*	0.0163	2.9E-15	25	47	61	1403	0.0469	0.056	0.0163	0.0026
*B*13:02*	*C*06:02*	0.0156	6.2E-26	24	2	95	1415	0.0169	0.0775	0.0156	0.0013
*B*18:01*	*C*12:03*	0.0156	4.2E-12	24	55	71	1386	0.0514	0.0618	0.0156	0.0032
*B*58:01*	*C*03:02*	0.0150	1.7E-22	23	33	26	1454	0.0365	0.0319	0.015	0.0012

*B*45:01*	*C*16:01*	0.0150	7.7E-15	23	22	100	1391	0.0293	0.0801	0.015	0.0023
*B*15:10*	*C*03:04*	0.0150	3.0E-14	23	14	145	1354	0.0241	0.1094	0.015	0.0026
*B*39:06*	*C*07:02*	0.0150	2.8E-11	23	2	413	1098	0.0163	0.2839	0.015	0.0046
*A*23:01*	*B*15:03*	0.0150	1.0E-09	23	99	49	1365	0.0794	0.0469	0.015	0.0037
*B*40:01*	*C*03:04*	0.0143	5.3E-16	22	8	146	1360	0.0195	0.1094	0.0143	0.0021
*A*26:01*	*C*12:03*	0.0143	3.6E-10	22	58	73	1383	0.0521	0.0618	0.0143	0.0032
*B*42:01*	*C*17:01*	0.0137	2.2E-27	21	5	38	1472	0.0169	0.0384	0.0137	0.0007
*B*15:01*	*C*03:03*	0.0137	6.2E-12	21	57	52	1406	0.0508	0.0475	0.0137	0.0024
*A*30:02*	*C*05:01*	0.0137	3.2E-06	21	95	75	1345	0.0755	0.0625	0.0137	0.0047
*B*57:01*	*C*07:01*	0.0130	4.2E-12	20	6	222	1288	0.0169	0.1576	0.013	0.0027
*B*58:02*	*C*06:02*	0.0124	1.9E-22	19	0	100	1417	0.0124	0.0775	0.0124	0.001
*B*45:01*	*C*06:02*	0.0124	8.6E-11	19	26	100	1391	0.0293	0.0775	0.0124	0.0023
*A*30:02*	*B*18:01*	0.0124	2.2E-06	19	97	60	1360	0.0755	0.0514	0.0124	0.0039
*A*68:02*	*B*53:01*	0.0117	1.2E-09	18	50	60	1408	0.0443	0.0508	0.0117	0.0022
*B*14:01*	*C*08:02*	0.0111	4.1E-17	17	4	92	1423	0.0137	0.071	0.0111	0.001
*B*51:01*	*C*14:02*	0.0111	3.4E-12	17	105	13	1401	0.0794	0.0195	0.0111	0.0016
*A*26:01*	*B*38:01*	0.0111	1.5E-11	17	63	29	1427	0.0521	0.0299	0.0111	0.0016
*B*52:01*	*C*03:03*	0.0111	1.2E-09	17	47	56	1416	0.0417	0.0475	0.0111	0.002
*A*23:01*	*C*02:10*	0.0111	1.7E-08	17	105	29	1385	0.0794	0.0299	0.0111	0.0024
*B*35:02*	*C*04:01*	0.0111	7.1E-08	17	1	491	1027	0.0117	0.3307	0.0111	0.0039
*B*50:01*	*C*06:02*	0.0104	8.5E-17	16	2	103	1415	0.0117	0.0775	0.0104	0.0009
*A*30:01*	*B*42:01*	0.0104	1.8E-15	16	63	10	1447	0.0514	0.0169	0.0104	0.0009
*B*35:20*	*C*04:01*	0.0104	1.7E-08	16	0	492	1028	0.0104	0.3307	0.0104	0.0034
*B*39:01*	*C*12:03*	0.0091	5.7E-09	14	22	81	1419	0.0234	0.0618	0.0091	0.0014
*B*55:01*	*C*03:03*	0.0085	1.3E-13	13	7	60	1456	0.013	0.0475	0.0085	0.0006
*B*51:01*	*C*15:09*	0.0085	1.3E-10	13	109	7	1407	0.0794	0.013	0.0085	0.001
*B*81:01*	*C*18:01*	0.0078	1.3E-20	12	2	17	1505	0.0091	0.0189	0.0078	0.0002
*B*57:03*	*C*18:01*	0.0078	7.1E-18	12	7	17	1500	0.0124	0.0189	0.0078	0.0002
*A*68:02*	*B*15:10*	0.0078	1.6E-08	12	56	25	1443	0.0443	0.0241	0.0078	0.0011
*B*37:01*	*C*06:02*	0.0072	1.2E-10	11	3	108	1414	0.0091	0.0775	0.0072	0.0007
*A*25:01*	*B*18:01*	0.0072	3.8E-10	11	9	68	1448	0.013	0.0514	0.0072	0.0007
*A*33:03*	*C*03:02*	0.0072	1.8E-09	11	23	38	1464	0.0221	0.0319	0.0072	0.0007
*B*07:02*	*C*15:05*	0.0072	5.9E-07	11	144	9	1372	0.1009	0.013	0.0072	0.0013
*B*57:01*	*C*06:02*	0.0072	1.1E-06	11	15	108	1402	0.0169	0.0775	0.0072	0.0013
*B*48:01*	*C*08:01*	0.0065	1.8E-15	10	14	7	1505	0.0156	0.0111	0.0065	0.0002
*B*27:05*	*C*02:02*	0.0065	1.1E-08	10	9	76	1441	0.0124	0.056	0.0065	0.0007
*A*25:01*	*C*12:03*	0.0065	5.7E-08	10	10	85	1431	0.013	0.0618	0.0065	0.0008
*B*41:02*	*C*17:01*	0.0059	9.5E-13	9	1	50	1476	0.0065	0.0384	0.0059	0.0003
*B*39:08*	*C*07:17*	0.0059	2.5E-11	9	74	1	1452	0.054	0.0065	0.0059	0.0004
*B*56:01*	*C*01:02*	0.0059	1.9E-08	9	0	207	1320	0.0059	0.1406	0.0059	0.0008
*B*40:02*	*C*07:01*	0.0059	2.5E-06	9	175	233	1119	0.1198	0.1576	0.0059	0.0189
*A*36:01*	*B*53:01*	0.0052	2.9E-07	8	8	70	1450	0.0104	0.0508	0.0052	0.0005
*B*48:01*	*C*08:03*	0.0046	6.9E-13	7	17	1	1511	0.0156	0.0052	0.0046	0.0001
*B*51:07*	*C*14:02*	0.0039	3.3E-11	6	0	24	1506	0.0039	0.0195	0.0039	0.0001
*B*15:16*	*C*14:02*	0.0039	2.7E-09	6	3	24	1503	0.0059	0.0195	0.0039	0.0001
*A*02:05*	*B*50:01*	0.0039	2.3E-06	6	32	12	1486	0.0247	0.0117	0.0039	0.0003
*B*15:18*	*C*07:04*	0.0026	7.1E-09	4	1	7	1524	0.0033	0.0072	0.0026	0
*A*69:01*	*B*55:01*	0.0026	3.1E-07	4	2	16	1514	0.0039	0.013	0.0026	0.0001
*B*42:02*	*C*17:01*	0.0026	2.0E-06	4	0	55	1477	0.0026	0.0384	0.0026	0.0001
*B*15:31*	*C*04:07*	0.0020	1.7E-09	3	0	0	1533	0.002	0.002	0.002	0
*A*02:07*	*B*46:01*	0.0020	1.7E-08	3	0	2	1531	0.002	0.0033	0.002	0

Supplementary Table S5. HLA 3-loci linkage disequilibria in the CAM cohort. For each statistically significant HLA pair identified in Supplementary Table S4, Fisher's exact test is used to assess linkage with a third gene. Multiple comparisons were addressed by Bonferroni correction. Number of distinct three-way Fisher's tests performed = N3 = 6092. P-value for three-way comparisons = 0.05/N3 = 8.207e-06 for a 95% confidence level.

HLA1	HLA2	HLA3	Population Frequency	p-value	All	Only HLA1&HLA2	Only HLA3	None	P(HLA1 & HLA2)	P(HLA3)	P(HLA1 & HLA2 & HLA3)	E(P(HLA1 & HLA2 & HLA3))
*A*24:02*	*C*01:02*	*B*40:02*	0.0671	5.1E-18	103	322	81	1030	0.2767	0.1198	0.0671	0.0331
*B*40:02*	*C*03:04*	*A*24:02*	0.0671	5.1E-18	103	81	322	1030	0.1198	0.2767	0.0671	0.0331
*A*24:02*	*C*03:05*	*B*40:02*	0.0671	5.1E-18	103	322	81	1030	0.2767	0.1198	0.0671	0.0331
*B*40:02*	*C*07:01*	*A*24:02*	0.0671	5.1E-18	103	81	322	1030	0.1198	0.2767	0.0671	0.0331
*A*68:03*	*B*35:43*	*C*07:02*	0.0638	3.1E-14	98	86	338	1014	0.1198	0.2839	0.0638	0.034
*A*68:03*	*B*39:05*	*C*07:02*	0.0638	3.1E-14	98	86	338	1014	0.1198	0.2839	0.0638	0.034
*B*35:12*	*C*04:01*	*A*02:01*	0.0469	5.4E-15	72	34	422	1008	0.069	0.3216	0.0469	0.0222
*A*02:06*	*B*39:08*	*C*07:02*	0.0449	7.0E-11	69	56	367	1044	0.0814	0.2839	0.0449	0.0231
*A*01:01*	*B*08:01*	*C*07:01*	0.0417	4.8E-24	64	55	178	1239	0.0775	0.1576	0.0417	0.0122
*B*07:02*	*C*07:02*	*A*03:01*	0.0319	2.9E-15	49	106	108	1273	0.1009	0.1022	0.0319	0.0103
*B*07:02*	*C*15:05*	*A*03:01*	0.0319	2.9E-15	49	106	108	1273	0.1009	0.1022	0.0319	0.0103
*A*29:02*	*B*44:03*	*C*16:01*	0.0299	2.3E-30	46	43	77	1370	0.0579	0.0801	0.0299	0.0046
*B*39:08*	*C*07:17*	*A*02:06*	0.0202	8.6E-15	31	52	94	1359	0.054	0.0814	0.0202	0.0044
*B*35:43*	*C*01:02*	*A*68:03*	0.0195	3.3E-06	30	78	154	1274	0.0703	0.1198	0.0195	0.0084
*A*30:01*	*B*42:01*	*C*17:01*	0.0189	3.3E-24	29	50	30	1427	0.0514	0.0384	0.0189	0.002
*A*33:01*	*B*14:02*	*C*08:02*	0.0176	9.8E-18	27	30	82	1397	0.0371	0.071	0.0176	0.0026
*B*15:03*	*C*02:02*	*A*23:01*	0.0150	1.0E-09	23	49	99	1365	0.0469	0.0794	0.015	0.0037
*A*23:01*	*C*02:10*	*B*15:03*	0.0150	1.0E-09	23	99	49	1365	0.0794	0.0469	0.015	0.0037
*A*26:01*	*B*38:01*	*C*12:03*	0.0143	3.6E-10	22	58	73	1383	0.0521	0.0618	0.0143	0.0032
*A*30:02*	*B*18:01*	*C*05:01*	0.0137	3.2E-06	21	95	75	1345	0.0755	0.0625	0.0137	0.0047
*B*18:01*	*C*12:03*	*A*30:02*	0.0124	2.2E-06	19	60	97	1360	0.0514	0.0755	0.0124	0.0039
*B*53:01*	*C*04:01*	*A*68:02*	0.0117	1.2E-09	18	60	50	1408	0.0508	0.0443	0.0117	0.0022
*B*15:10*	*C*03:04*	*A*68:02*	0.0078	1.6E-08	12	25	56	1443	0.0241	0.0443	0.0078	0.0011
*B*18:01*	*C*05:01*	*A*25:01*	0.0072	3.8E-10	11	68	9	1448	0.0514	0.013	0.0072	0.0007
*A*25:01*	*C*12:03*	*B*18:01*	0.0072	3.8E-10	11	9	68	1448	0.013	0.0514	0.0072	0.0007
*B*58:01*	*C*03:02*	*A*02:05*	0.0059	4.3E-06	9	47	29	1451	0.0365	0.0247	0.0059	0.0009
*B*58:01*	*C*07:01*	*A*02:05*	0.0059	4.3E-06	9	47	29	1451	0.0365	0.0247	0.0059	0.0009
*B*53:01*	*C*04:01*	*A*36:01*	0.0052	2.9E-07	8	70	8	1450	0.0508	0.0104	0.0052	0.0005
*B*50:01*	*C*06:02*	*A*02:05*	0.0039	2.3E-06	6	12	32	1486	0.0117	0.0247	0.0039	0.0003
*B*55:01*	*C*03:03*	*A*69:01*	0.0026	3.1E-07	4	16	2	1514	0.013	0.0039	0.0026	0.0001

Supplementary Table S6. HLA 2-loci linkage disequilibria in the pooled MEX/CAM cohort. Table headers same as Supplementary Table S2. Multiple comparisons were addressed by Bonferroni correction. Number of distinct two-way tests performed = N2 = 21421. P-value for two-way comparisons = 0.05/N2 = 2.334e-06 for a 95% confidence level.

HLA1	HLA2	Population frequency	p-value	Both	Only HLA1	Only HLA2	Neither	P(HLA1)	P(HLA2)	P(HLA1 & HLA2)	E(P(HLA1 & HLA2))
*B*35:01*	*C*04:01*	0.1293	1.9E-96	420	163	687	1979	0.1794	0.3407	0.1293	0.0611
*B*39:05*	*C*07:02*	0.0976	3.8E-144	317	20	726	2186	0.1037	0.321	0.0976	0.0333
*B*35:12*	*C*04:01*	0.0751	7.5E-104	244	13	863	2129	0.0791	0.3407	0.0751	0.027
*B*07:02*	*C*07:02*	0.0742	8.0E-90	241	34	802	2172	0.0846	0.321	0.0742	0.0272
*A*68:03*	*C*07:02*	0.0714	3.1E-41	232	127	811	2079	0.1105	0.321	0.0714	0.0355
*A*02:06*	*C*07:02*	0.0634	4.3E-14	206	218	837	1988	0.1305	0.321	0.0634	0.0419
*A*24:02*	*B*40:02*	0.0563	2.8E-19	183	717	199	2150	0.277	0.1176	0.0563	0.0326
*A*02:01*	*B*35:12*	0.0517	6.3E-24	168	990	89	2002	0.3564	0.0791	0.0517	0.0282
*B*14:02*	*C*08:02*	0.0511	8.0E-195	166	18	77	2988	0.0566	0.0748	0.0511	0.0042
*B*40:02*	*C*03:05*	0.0468	1.3E-104	152	230	59	2808	0.1176	0.0649	0.0468	0.0076
*A*68:03*	*B*39:05*	0.0468	2.8E-67	152	207	185	2705	0.1105	0.1037	0.0468	0.0115
*B*40:02*	*C*03:04*	0.0468	3.7E-49	152	230	255	2612	0.1176	0.1253	0.0468	0.0147
*B*35:43*	*C*01:02*	0.0403	2.7E-114	131	6	301	2811	0.0422	0.133	0.0403	0.0056
*B*35:17*	*C*04:01*	0.0403	3.7E-53	131	8	976	2134	0.0428	0.3407	0.0403	0.0146
*B*39:06*	*C*07:02*	0.0400	3.7E-56	130	8	913	2198	0.0425	0.321	0.04	0.0136
*A*24:02*	*C*03:05*	0.0400	1.5E-26	130	770	81	2268	0.277	0.0649	0.04	0.018
*B*08:01*	*C*07:01*	0.0397	3.2E-91	129	25	325	2770	0.0474	0.1397	0.0397	0.0066
*A*01:01*	*C*07:01*	0.0369	1.7E-38	120	148	334	2647	0.0825	0.1397	0.0369	0.0115
*B*15:01*	*C*01:02*	0.0354	7.0E-57	115	76	317	2741	0.0588	0.133	0.0354	0.0078
*B*48:01*	*C*08:01*	0.0332	2.0E-133	108	27	54	3060	0.0416	0.0499	0.0332	0.0021
*B*44:03*	*C*16:01*	0.0332	1.1E-78	108	106	112	2923	0.0659	0.0677	0.0332	0.0045
*A*02:01*	*C*03:03*	0.0314	3.5E-07	102	1056	90	2001	0.3564	0.0591	0.0314	0.0211
*B*51:01*	*C*15:02*	0.0302	2.2E-75	98	188	45	2918	0.088	0.044	0.0302	0.0039
*A*24:02*	*B*39:06*	0.0299	2.2E-26	97	803	41	2308	0.277	0.0425	0.0299	0.0118
*B*53:01*	*C*04:01*	0.0299	3.4E-24	97	28	1010	2114	0.0385	0.3407	0.0299	0.0131
*B*39:08*	*C*07:02*	0.0289	1.6E-36	94	10	949	2196	0.032	0.321	0.0289	0.0103
*A*02:06*	*B*39:05*	0.0280	3.1E-13	91	333	246	2579	0.1305	0.1037	0.028	0.0135
*A*29:02*	*C*16:01*	0.0274	4.3E-68	89	71	131	2958	0.0492	0.0677	0.0274	0.0033
*B*49:01*	*C*07:01*	0.0271	5.8E-65	88	12	366	2783	0.0308	0.1397	0.0271	0.0043
*A*68:01*	*C*03:04*	0.0268	7.2E-10	87	287	320	2555	0.1151	0.1253	0.0268	0.0144
*A*03:01*	*B*07:02*	0.0265	7.1E-29	86	207	189	2767	0.0902	0.0846	0.0265	0.0076
*B*39:01*	*C*07:02*	0.0265	1.6E-19	86	34	957	2172	0.0369	0.321	0.0265	0.0119
*B*44:02*	*C*05:01*	0.0255	4.7E-92	83	18	109	3039	0.0311	0.0591	0.0255	0.0018
*A*29:02*	*B*44:03*	0.0249	4.3E-58	81	79	133	2956	0.0492	0.0659	0.0249	0.0032
*B*40:02*	*C*04:01*	0.0240	6.1E-10	78	304	1029	1838	0.1176	0.3407	0.024	0.0401
*A*02:01*	*B*35:17*	0.0237	1.3E-06	77	1081	62	2029	0.3564	0.0428	0.0237	0.0152
*B*38:01*	*C*12:03*	0.0234	3.1E-81	76	22	110	3041	0.0302	0.0572	0.0234	0.0017
*B*39:02*	*C*07:02*	0.0234	2.3E-20	76	23	967	2183	0.0305	0.321	0.0234	0.0098
*B*40:02*	*C*07:02*	0.0228	4.4E-09	74	308	969	1898	0.1176	0.321	0.0228	0.0377
*A*01:01*	*B*08:01*	0.0222	3.3E-40	72	196	82	2899	0.0825	0.0474	0.0222	0.0039
*B*15:15*	*C*01:02*	0.0209	1.7E-53	68	7	364	2810	0.0231	0.133	0.0209	0.0031
*A*33:01*	*C*08:02*	0.0209	4.2E-51	68	44	175	2962	0.0345	0.0748	0.0209	0.0026
*B*52:01*	*C*03:03*	0.0200	5.6E-44	65	84	127	2973	0.0459	0.0591	0.02	0.0027
*B*13:02*	*C*06:02*	0.0191	2.9E-69	62	2	193	2992	0.0197	0.0785	0.0191	0.0015
*A*33:01*	*B*14:02*	0.0191	2.3E-51	62	50	122	3015	0.0345	0.0566	0.0191	0.002
*A*02:01*	*B*44:02*	0.0188	4.0E-07	61	1097	40	2051	0.3564	0.0311	0.0188	0.0111
*B*18:01*	*C*05:01*	0.0185	2.7E-38	60	87	132	2970	0.0452	0.0591	0.0185	0.0027
*A*02:01*	*B*15:15*	0.0179	1.1E-13	58	1100	17	2074	0.3564	0.0231	0.0179	0.0082

*B*07:02*	*C*04:01*	0.0179	1.1E-06	58	217	1049	1925	0.0846	0.3407	0.0179	0.0288
*B*15:01*	*C*03:03*	0.0172	9.2E-27	56	135	136	2922	0.0588	0.0591	0.0172	0.0035
*B*52:01*	*C*12:02*	0.0166	2.3E-64	54	95	13	3087	0.0459	0.0206	0.0166	0.0009
*B*18:01*	*C*12:03*	0.0154	7.1E-28	50	97	136	2966	0.0452	0.0572	0.0154	0.0026
*B*15:03*	*C*02:10*	0.0151	7.7E-73	49	50	7	3143	0.0305	0.0172	0.0151	0.0005
*B*14:01*	*C*08:02*	0.0148	5.7E-45	48	12	195	2994	0.0185	0.0748	0.0148	0.0014
*B*35:02*	*C*04:01*	0.0148	6.0E-22	48	1	1059	2141	0.0151	0.3407	0.0148	0.0051
*A*01:01*	*C*06:02*	0.0148	1.6E-08	48	220	207	2774	0.0825	0.0785	0.0148	0.0065
*A*01:01*	*C*07:02*	0.0145	3.5E-08	47	221	996	1985	0.0825	0.321	0.0145	0.0265
*A*30:01*	*C*17:01*	0.0139	7.3E-35	45	101	62	3041	0.0449	0.0329	0.0139	0.0015
*A*30:01*	*C*06:02*	0.0139	4.7E-17	45	101	210	2893	0.0449	0.0785	0.0139	0.0035
*B*58:01*	*C*07:01*	0.0135	2.7E-17	44	40	410	2755	0.0259	0.1397	0.0135	0.0036
*A*02:06*	*C*08:01*	0.0135	8.8E-07	44	380	118	2707	0.1305	0.0499	0.0135	0.0065
*B*35:01*	*C*01:02*	0.0135	2.2E-06	44	539	388	2278	0.1794	0.133	0.0135	0.0239
*A*26:01*	*C*12:03*	0.0132	2.6E-21	43	101	143	2962	0.0443	0.0572	0.0132	0.0025
*A*30:02*	*C*05:01*	0.0126	5.0E-16	41	129	151	2928	0.0523	0.0591	0.0126	0.0031
*A*02:06*	*B*39:08*	0.0123	2.7E-11	40	384	64	2761	0.1305	0.032	0.0123	0.0042
*B*27:05*	*C*02:02*	0.0120	3.0E-37	39	24	119	3067	0.0194	0.0486	0.012	0.0009
*A*68:03*	*B*35:43*	0.0120	7.5E-09	39	320	98	2792	0.1105	0.0422	0.012	0.0047
*B*51:01*	*C*14:02*	0.0117	3.9E-28	38	248	18	2945	0.088	0.0172	0.0117	0.0015
*A*02:05*	*C*07:01*	0.0117	6.1E-15	38	35	416	2760	0.0225	0.1397	0.0117	0.0031
*B*45:01*	*C*16:01*	0.0114	3.4E-23	37	44	183	2985	0.0249	0.0677	0.0114	0.0017
*B*45:01*	*C*06:02*	0.0114	7.8E-21	37	44	218	2950	0.0249	0.0785	0.0114	0.002
*B*42:01*	*C*17:01*	0.0111	4.4E-51	36	5	71	3137	0.0126	0.0329	0.0111	0.0004
*B*51:01*	*C*15:09*	0.0111	9.0E-30	36	250	11	2952	0.088	0.0145	0.0111	0.0013
*B*15:03*	*C*02:02*	0.0111	1.0E-23	36	63	122	3028	0.0305	0.0486	0.0111	0.0015
*B*35:01*	*C*07:01*	0.0111	1.1E-10	36	547	418	2248	0.1794	0.1397	0.0111	0.0251
*A*26:01*	*B*38:01*	0.0108	3.1E-24	35	109	63	3042	0.0443	0.0302	0.0108	0.0013
*B*40:01*	*C*03:04*	0.0108	2.5E-23	35	11	372	2831	0.0142	0.1253	0.0108	0.0018
*B*57:01*	*C*07:01*	0.0108	6.7E-17	35	22	419	2773	0.0175	0.1397	0.0108	0.0025
*A*23:01*	*C*06:02*	0.0108	2.3E-07	35	146	220	2848	0.0557	0.0785	0.0108	0.0044
*B*15:10*	*C*03:04*	0.0105	1.2E-19	34	17	373	2825	0.0157	0.1253	0.0105	0.002
*A*30:02*	*B*18:01*	0.0105	2.0E-14	34	136	113	2966	0.0523	0.0452	0.0105	0.0024
*B*58:01*	*C*03:02*	0.0102	1.8E-36	33	51	37	3128	0.0259	0.0215	0.0102	0.0006
*A*30:01*	*B*13:02*	0.0102	4.8E-29	33	113	31	3072	0.0449	0.0197	0.0102	0.0009
*B*35:14*	*C*04:01*	0.0102	3.7E-12	33	4	1074	2138	0.0114	0.3407	0.0102	0.0039
*B*39:11*	*C*07:02*	0.0098	1.2E-16	32	0	1011	2206	0.0098	0.321	0.0098	0.0032
*A*01:01*	*B*57:01*	0.0095	8.6E-20	31	237	26	2955	0.0825	0.0175	0.0095	0.0014
*A*23:01*	*B*15:03*	0.0095	1.9E-16	31	150	68	3000	0.0557	0.0305	0.0095	0.0017
*B*50:01*	*C*06:02*	0.0092	5.7E-26	30	10	225	2984	0.0123	0.0785	0.0092	0.001
*B*58:02*	*C*06:02*	0.0086	2.7E-32	28	0	227	2994	0.0086	0.0785	0.0086	0.0007
*B*57:01*	*C*06:02*	0.0086	5.3E-17	28	29	227	2965	0.0175	0.0785	0.0086	0.0014
*B*14:02*	*C*07:02*	0.0086	1.4E-07	28	156	1015	2050	0.0566	0.321	0.0086	0.0182
*A*68:02*	*B*53:01*	0.0083	3.2E-14	27	98	98	3026	0.0385	0.0385	0.0083	0.0015
*B*44:03*	*C*07:02*	0.0083	1.9E-11	27	187	1016	2019	0.0659	0.321	0.0083	0.0211
*A*30:01*	*B*42:01*	0.0080	3.5E-26	26	120	15	3088	0.0449	0.0126	0.008	0.0006
*A*30:02*	*C*07:02*	0.0080	4.7E-07	26	144	1017	2062	0.0523	0.321	0.008	0.0168
*B*40:02*	*C*03:06*	0.0077	4.3E-20	25	357	4	2863	0.1176	0.0089	0.0077	0.001
*B*15:01*	*C*07:02*	0.0077	6.9E-10	25	166	1018	2040	0.0588	0.321	0.0077	0.0189
*B*55:01*	*C*03:03*	0.0074	6.3E-23	24	10	168	3047	0.0105	0.0591	0.0074	0.0006
*B*40:08*	*C*03:04*	0.0074	1.4E-16	24	7	383	2835	0.0095	0.1253	0.0074	0.0012
*B*41:01*	*C*07:01*	0.0074	1.2E-09	24	23	430	2772	0.0145	0.1397	0.0074	0.002
*B*40:02*	*C*07:01*	0.0071	2.6E-07	23	359	431	2436	0.1176	0.1397	0.0071	0.0164

B*27:05	C*01:02	0.0071	2.2E-06	23	40	409	2777	0.0194	0.133	0.0071	0.0026
B*37:01	C*06:02	0.0068	3.8E-22	22	3	233	2991	0.0077	0.0785	0.0068	0.0006
A*25:01	C*12:03	0.0068	9.7E-17	22	22	164	3041	0.0135	0.0572	0.0068	0.0008
B*35:20	C*04:01	0.0068	4.5E-11	22	0	1085	2142	0.0068	0.3407	0.0068	0.0023
B*39:01	C*12:03	0.0068	6.3E-07	22	98	164	2965	0.0369	0.0572	0.0068	0.0021
A*68:02	B*14:02	0.0068	1.1E-06	22	103	162	2962	0.0385	0.0566	0.0068	0.0022
B*41:01	C*17:01	0.0065	6.2E-20	21	26	86	3116	0.0145	0.0329	0.0065	0.0005
A*25:01	B*18:01	0.0062	2.5E-16	20	24	127	3078	0.0135	0.0452	0.0062	0.0006
B*35:08	C*04:01	0.0062	2.2E-07	20	3	1087	2139	0.0071	0.3407	0.0062	0.0024
A*23:01	B*49:01	0.0062	3.1E-07	20	161	80	2988	0.0557	0.0308	0.0062	0.0017
B*56:01	C*01:02	0.0058	2.6E-15	19	2	413	2815	0.0065	0.133	0.0058	0.0009
A*23:01	C*02:10	0.0058	4.1E-11	19	162	37	3031	0.0557	0.0172	0.0058	0.001
B*57:03	C*18:01	0.0055	5.7E-28	18	17	20	3194	0.0108	0.0117	0.0055	0.0001
B*51:02	C*08:01	0.0055	8.8E-21	18	4	144	3083	0.0068	0.0499	0.0055	0.0003
B*15:30	C*01:02	0.0055	1.8E-14	18	2	414	2815	0.0062	0.133	0.0055	0.0008
B*15:17	C*07:01	0.0055	2.7E-13	18	3	436	2792	0.0065	0.1397	0.0055	0.0009
B*35:16	C*04:01	0.0055	3.5E-09	18	0	1089	2142	0.0055	0.3407	0.0055	0.0019
B*35:01	C*08:02	0.0055	2.0E-06	18	565	225	2441	0.1794	0.0748	0.0055	0.0134
A*02:05	B*41:01	0.0049	6.6E-16	16	57	31	3145	0.0225	0.0145	0.0049	0.0003
B*40:05	C*03:04	0.0049	3.4E-13	16	2	391	2840	0.0055	0.1253	0.0049	0.0007
A*68:02	B*15:10	0.0049	2.0E-11	16	109	35	3089	0.0385	0.0157	0.0049	0.0006
B*48:01	C*08:03	0.0046	1.4E-20	15	120	1	3113	0.0416	0.0049	0.0046	0.0002
B*41:02	C*17:01	0.0046	1.6E-20	15	3	92	3139	0.0055	0.0329	0.0046	0.0002
B*07:02	C*15:05	0.0046	3.9E-08	15	260	20	2954	0.0846	0.0108	0.0046	0.0009
A*74:01	B*15:03	0.0043	6.6E-11	14	35	85	3115	0.0151	0.0305	0.0043	0.0005
B*81:01	C*18:01	0.0040	4.2E-24	13	3	25	3208	0.0049	0.0117	0.004	0.0001
A*33:03	C*03:02	0.0040	1.3E-11	13	37	57	3142	0.0154	0.0215	0.004	0.0003
A*31:01	B*51:02	0.0037	1.0E-06	12	363	10	2864	0.1154	0.0068	0.0037	0.0008
A*36:01	B*53:01	0.0034	4.5E-11	11	10	114	3114	0.0065	0.0385	0.0034	0.0002
B*49:01	C*04:01	0.0034	1.4E-07	11	89	1096	2053	0.0308	0.3407	0.0034	0.0105
A*02:05	B*58:01	0.0034	1.7E-06	11	62	73	3103	0.0225	0.0259	0.0034	0.0006
B*07:05	C*15:05	0.0031	5.4E-15	10	13	25	3201	0.0071	0.0108	0.0031	0.0001
A*74:01	C*02:10	0.0031	4.9E-09	10	39	46	3154	0.0151	0.0172	0.0031	0.0003
A*33:03	B*58:01	0.0031	3.4E-07	10	40	74	3125	0.0154	0.0259	0.0031	0.0004
B*39:08	C*07:17	0.0028	2.4E-13	9	95	1	3144	0.032	0.0031	0.0028	0.0001
A*02:05	B*50:01	0.0028	1.4E-07	9	64	31	3145	0.0225	0.0123	0.0028	0.0003
A*03:01	B*39:05	0.0025	5.4E-07	8	285	329	2627	0.0902	0.1037	0.0025	0.0094
A*24:25	B*48:01	0.0025	6.0E-07	8	12	127	3102	0.0062	0.0416	0.0025	0.0003
B*40:11	C*03:04	0.0025	2.0E-06	8	2	399	2840	0.0031	0.1253	0.0025	0.0004
B*15:18	C*07:04	0.0022	2.6E-15	7	1	16	3225	0.0025	0.0071	0.0022	0
B*51:07	C*14:02	0.0022	2.4E-12	7	1	49	3192	0.0025	0.0172	0.0022	0
B*15:16	C*14:02	0.0022	9.6E-11	7	4	49	3189	0.0034	0.0172	0.0022	0.0001
B*15:39	C*03:03	0.0022	1.7E-08	7	1	185	3056	0.0025	0.0591	0.0022	0.0001
A*69:01	B*55:01	0.0018	1.7E-10	6	4	28	3211	0.0031	0.0105	0.0018	0
B*42:02	C*17:01	0.0018	1.1E-09	6	0	101	3142	0.0018	0.0329	0.0018	0.0001
A*01:02	B*49:01	0.0018	2.0E-08	6	2	94	3147	0.0025	0.0308	0.0018	0.0001
B*57:02	C*18:01	0.0015	3.4E-09	5	2	33	3209	0.0022	0.0117	0.0015	0
B*15:31	C*04:07	0.0009	1.8E-10	3	0	0	3246	0.0009	0.0009	0.0009	0
A*02:07	B*46:01	0.0009	1.8E-09	3	0	2	3244	0.0009	0.0015	0.0009	0
B*27:05	C*07:02	0.0009	1.6E-07	3	60	1040	2146	0.0194	0.321	0.0009	0.0062
B*15:09	C*07:04	0.0009	3.1E-07	3	0	20	3226	0.0009	0.0071	0.0009	0
A*11:01	B*39:05	0.0009	8.6E-07	3	194	334	2718	0.0606	0.1037	0.0009	0.0063
A*23:01	B*39:05	0.0003	5.2E-08	1	180	336	2732	0.0557	0.1037	0.0003	0.0058

Supplementary Table S7. HLA 3-loci linkage disequilibria in the pooled MEX/CAM cohort. For each statistically significant HLA pair identified in Supplementary Table S6, Fisher's exact test is used to assess linkage with a third gene. Multiple comparisons were addressed by Bonferroni correction. Number of distinct three-way Fisher's tests performed = N3 = 11919. P-value for three-way comparisons = 0.05/N3 = 4.195e-06 for a 95% confidence level.

HLA1	HLA2	HLA3	Population Frequency	p-value	All	Only HLA1&HLA2	Only HLA3	None	P(HLA1 & HLA2)	P(HLA3)	P(HLA1 & HLA2 & HLA3)	E(P(HLA1 & HLA2 & HLA3))
*A*68:03*	*B*35:43*	*C*07:02*	0.0714	3.1E-41	232	127	811	2079	0.1105	0.321	0.0714	0.0355
*A*02:06*	*B*39:05*	*C*07:02*	0.0634	4.3E-14	206	218	837	1988	0.1305	0.321	0.0634	0.0419
*A*02:06*	*B*39:08*	*C*07:02*	0.0634	4.3E-14	206	218	837	1988	0.1305	0.321	0.0634	0.0419
*B*40:02*	*C*03:04*	*A*24:02*	0.0563	2.8E-19	183	199	717	2150	0.1176	0.277	0.0563	0.0326
*B*40:02*	*C*03:06*	*A*24:02*	0.0563	2.8E-19	183	199	717	2150	0.1176	0.277	0.0563	0.0326
*B*40:02*	*C*04:01*	*A*24:02*	0.0563	2.8E-19	183	199	717	2150	0.1176	0.277	0.0563	0.0326
*B*40:02*	*C*07:01*	*A*24:02*	0.0563	2.8E-19	183	199	717	2150	0.1176	0.277	0.0563	0.0326
*B*40:02*	*C*07:02*	*A*24:02*	0.0563	2.8E-19	183	199	717	2150	0.1176	0.277	0.0563	0.0326
*A*02:01*	*C*03:03*	*B*35:12*	0.0517	6.3E-24	168	990	89	2002	0.3564	0.0791	0.0517	0.0282
*B*35:12*	*C*04:01*	*A*02:01*	0.0517	6.3E-24	168	89	990	2002	0.0791	0.3564	0.0517	0.0282
*A*68:03*	*C*07:02*	*B*39:05*	0.0468	2.8E-67	152	207	185	2705	0.1105	0.1037	0.0468	0.0115
*A*24:02*	*B*39:06*	*C*03:05*	0.0400	1.5E-26	130	770	81	2268	0.277	0.0649	0.04	0.018
*A*24:02*	*B*40:02*	*C*03:05*	0.0400	1.5E-26	130	770	81	2268	0.277	0.0649	0.04	0.018
*A*01:01*	*B*57:01*	*C*07:01*	0.0369	1.7E-38	120	148	334	2647	0.0825	0.1397	0.0369	0.0115
*A*02:01*	*B*35:17*	*C*03:03*	0.0314	3.5E-07	102	1056	90	2001	0.3564	0.0591	0.0314	0.0211
*A*02:01*	*B*44:02*	*C*03:03*	0.0314	3.5E-07	102	1056	90	2001	0.3564	0.0591	0.0314	0.0211
*B*39:06*	*C*07:02*	*A*24:02*	0.0299	2.2E-26	97	41	803	2308	0.0425	0.277	0.0299	0.0118
*A*02:06*	*C*08:01*	*B*39:05*	0.0280	3.1E-13	91	333	246	2579	0.1305	0.1037	0.028	0.0135
*A*29:02*	*B*44:03*	*C*16:01*	0.0274	4.3E-68	89	71	131	2958	0.0492	0.0677	0.0274	0.0033
*B*07:02*	*C*04:01*	*A*03:01*	0.0265	7.1E-29	86	189	207	2767	0.0846	0.0902	0.0265	0.0076
*B*07:02*	*C*07:02*	*A*03:01*	0.0265	7.1E-29	86	189	207	2767	0.0846	0.0902	0.0265	0.0076
*B*07:02*	*C*15:05*	*A*03:01*	0.0265	7.1E-29	86	189	207	2767	0.0846	0.0902	0.0265	0.0076
*B*44:03*	*C*07:02*	*A*29:02*	0.0249	4.3E-58	81	133	79	2956	0.0659	0.0492	0.0249	0.0032
*B*35:17*	*C*04:01*	*A*02:01*	0.0237	1.3E-06	77	62	1081	2029	0.0428	0.3564	0.0237	0.0152
*A*01:01*	*C*06:02*	*B*08:01*	0.0222	3.3E-40	72	196	82	2899	0.0825	0.0474	0.0222	0.0039
*A*01:01*	*C*07:01*	*B*08:01*	0.0222	3.3E-40	72	196	82	2899	0.0825	0.0474	0.0222	0.0039
*A*01:01*	*C*07:02*	*B*08:01*	0.0222	3.3E-40	72	196	82	2899	0.0825	0.0474	0.0222	0.0039
*B*14:02*	*C*07:02*	*A*33:01*	0.0191	2.3E-51	62	122	50	3015	0.0566	0.0345	0.0191	0.002
*A*33:01*	*C*08:02*	*B*14:02*	0.0191	2.3E-51	62	50	122	3015	0.0345	0.0566	0.0191	0.002
*B*44:02*	*C*05:01*	*A*02:01*	0.0188	4.0E-07	61	40	1097	2051	0.0311	0.3564	0.0188	0.0111
*B*15:15*	*C*01:02*	*A*02:01*	0.0179	1.1E-13	58	17	1100	2074	0.0231	0.3564	0.0179	0.0082

*A*02:01*	*C*03:03*	*B*15:15*	0.0179	1.1E-13	58	1100	17	2074	0.3564	0.0231	0.0179	0.0082
*A*30:01*	*B*13:02*	*C*17:01*	0.0139	7.3E-35	45	101	62	3041	0.0449	0.0329	0.0139	0.0015
*A*30:01*	*B*42:01*	*C*17:01*	0.0139	7.3E-35	45	101	62	3041	0.0449	0.0329	0.0139	0.0015
*A*30:02*	*B*18:01*	*C*05:01*	0.0126	5.0E-16	41	129	151	2928	0.0523	0.0591	0.0126	0.0031
*B*39:08*	*C*07:17*	*A*02:06*	0.0123	2.7E-11	40	64	384	2761	0.032	0.1305	0.0123	0.0042
*A*02:06*	*C*08:01*	*B*39:08*	0.0123	2.7E-11	40	384	64	2761	0.1305	0.032	0.0123	0.0042
*A*24:02*	*B*39:06*	*C*06:02*	0.0123	3.8E-06	40	860	215	2134	0.277	0.0785	0.0123	0.0217
*A*24:02*	*B*40:02*	*C*06:02*	0.0123	3.8E-06	40	860	215	2134	0.277	0.0785	0.0123	0.0217
*B*35:43*	*C*01:02*	*A*68:03*	0.0120	7.5E-09	39	98	320	2792	0.0422	0.1105	0.012	0.0047
*A*02:05*	*B*50:01*	*C*07:01*	0.0117	6.1E-15	38	35	416	2760	0.0225	0.1397	0.0117	0.0031
*A*02:05*	*B*58:01*	*C*07:01*	0.0117	6.1E-15	38	35	416	2760	0.0225	0.1397	0.0117	0.0031
*A*23:01*	*B*49:01*	*C*06:02*	0.0108	2.3E-07	35	146	220	2848	0.0557	0.0785	0.0108	0.0044
*A*26:01*	*C*12:03*	*B*38:01*	0.0108	3.1E-24	35	109	63	3042	0.0443	0.0302	0.0108	0.0013
*A*30:02*	*C*07:02*	*B*18:01*	0.0105	2.0E-14	34	136	113	2966	0.0523	0.0452	0.0105	0.0024
*B*18:01*	*C*12:03*	*A*30:02*	0.0105	2.0E-14	34	113	136	2966	0.0452	0.0523	0.0105	0.0024
*A*30:01*	*C*06:02*	*B*13:02*	0.0102	4.8E-29	33	113	31	3072	0.0449	0.0197	0.0102	0.0009
*B*57:01*	*C*06:02*	*A*01:01*	0.0095	8.6E-20	31	26	237	2955	0.0175	0.0825	0.0095	0.0014
*A*01:01*	*C*07:02*	*B*57:01*	0.0095	8.6E-20	31	237	26	2955	0.0825	0.0175	0.0095	0.0014
*B*15:03*	*C*02:02*	*A*23:01*	0.0095	1.9E-16	31	68	150	3000	0.0305	0.0557	0.0095	0.0017
*B*15:03*	*C*02:10*	*A*23:01*	0.0095	1.9E-16	31	68	150	3000	0.0305	0.0557	0.0095	0.0017
*A*23:01*	*C*06:02*	*B*15:03*	0.0095	1.9E-16	31	150	68	3000	0.0557	0.0305	0.0095	0.0017
*B*53:01*	*C*04:01*	*A*68:02*	0.0083	3.2E-14	27	98	98	3026	0.0385	0.0385	0.0083	0.0015
*A*30:01*	*C*06:02*	*B*42:01*	0.0080	3.5E-26	26	120	15	3088	0.0449	0.0126	0.008	0.0006
*A*68:02*	*B*15:10*	*C*08:02*	0.0077	3.2E-06	25	100	218	2906	0.0385	0.0748	0.0077	0.0029
*A*68:02*	*B*53:01*	*C*08:02*	0.0077	3.2E-06	25	100	218	2906	0.0385	0.0748	0.0077	0.0029
*A*68:03*	*B*35:43*	*C*07:01*	0.0071	3.4E-06	23	336	431	2459	0.1105	0.1397	0.0071	0.0154
*A*68:03*	*B*39:05*	*C*07:01*	0.0071	3.4E-06	23	336	431	2459	0.1105	0.1397	0.0071	0.0154
*A*25:01*	*B*18:01*	*C*12:03*	0.0068	9.7E-17	22	22	164	3041	0.0135	0.0572	0.0068	0.0008
*B*14:02*	*C*07:02*	*A*68:02*	0.0068	1.1E-06	22	162	103	2962	0.0566	0.0385	0.0068	0.0022
*B*14:02*	*C*08:02*	*A*68:02*	0.0068	1.1E-06	22	162	103	2962	0.0566	0.0385	0.0068	0.0022
*B*49:01*	*C*04:01*	*A*23:01*	0.0062	3.1E-07	20	80	161	2988	0.0308	0.0557	0.0062	0.0017
*B*49:01*	*C*07:01*	*A*23:01*	0.0062	3.1E-07	20	80	161	2988	0.0308	0.0557	0.0062	0.0017
*B*18:01*	*C*05:01*	*A*25:01*	0.0062	2.5E-16	20	127	24	3078	0.0452	0.0135	0.0062	0.0006
*A*23:01*	*B*39:05*	*C*02:10*	0.0058	4.1E-11	19	162	37	3031	0.0557	0.0172	0.0058	0.001
*A*23:01*	*B*49:01*	*C*02:10*	0.0058	4.1E-11	19	162	37	3031	0.0557	0.0172	0.0058	0.001
*B*41:01*	*C*07:01*	*A*02:05*	0.0049	6.6E-16	16	31	57	3145	0.0145	0.0225	0.0049	0.0003

*B*41:01*	*C*17:01*	*A*02:05*	0.0049	6.6E-16	16	31	57	3145	0.0145	0.0225	0.0049	0.0003
*B*15:10*	*C*03:04*	*A*68:02*	0.0049	2.0E-11	16	35	109	3089	0.0157	0.0385	0.0049	0.0006
*B*15:03*	*C*02:02*	*A*74:01*	0.0043	6.6E-11	14	85	35	3115	0.0305	0.0151	0.0043	0.0005
*A*74:01*	*C*02:10*	*B*15:03*	0.0043	6.6E-11	14	35	85	3115	0.0151	0.0305	0.0043	0.0005
*A*33:03*	*B*58:01*	*C*03:02*	0.0040	1.3E-11	13	37	57	3142	0.0154	0.0215	0.004	0.0003
*B*51:02*	*C*08:01*	*A*31:01*	0.0037	1.0E-06	12	10	363	2864	0.0068	0.1154	0.0037	0.0008
*B*58:01*	*C*03:02*	*A*02:05*	0.0034	1.7E-06	11	73	62	3103	0.0259	0.0225	0.0034	0.0006
*B*14:01*	*C*08:02*	*A*32:01*	0.0034	3.8E-06	11	49	100	3089	0.0185	0.0342	0.0034	0.0006
*B*53:01*	*C*04:01*	*A*36:01*	0.0034	4.5E-11	11	114	10	3114	0.0385	0.0065	0.0034	0.0002
*B*58:01*	*C*07:01*	*A*33:03*	0.0031	3.4E-07	10	74	40	3125	0.0259	0.0154	0.0031	0.0004
*B*50:01*	*C*06:02*	*A*02:05*	0.0028	1.4E-07	9	31	64	3145	0.0123	0.0225	0.0028	0.0003
*B*39:05*	*C*07:02*	*A*03:01*	0.0025	5.4E-07	8	329	285	2627	0.1037	0.0902	0.0025	0.0094
*B*48:01*	*C*08:01*	*A*24:25*	0.0025	6.0E-07	8	127	12	3102	0.0416	0.0062	0.0025	0.0003
*B*48:01*	*C*08:03*	*A*24:25*	0.0025	6.0E-07	8	127	12	3102	0.0416	0.0062	0.0025	0.0003
*B*49:01*	*C*04:01*	*A*01:02*	0.0018	2.0E-08	6	94	2	3147	0.0308	0.0025	0.0018	0.0001
*B*49:01*	*C*07:01*	*A*01:02*	0.0018	2.0E-08	6	94	2	3147	0.0308	0.0025	0.0018	0.0001
*B*55:01*	*C*03:03*	*A*69:01*	0.0018	1.7E-10	6	28	4	3211	0.0105	0.0031	0.0018	0
*B*39:05*	*C*07:02*	*A*11:01*	0.0009	8.6E-07	3	334	194	2718	0.1037	0.0606	0.0009	0.0063
*A*23:01*	*C*06:02*	*B*39:05*	0.0003	5.2E-08	1	180	336	2732	0.0557	0.1037	0.0003	0.0058
*B*39:05*	*C*07:02*	*A*23:01*	0.0003	5.2E-08	1	336	180	2732	0.1037	0.0557	0.0003	0.0058

Supplementary Table S8. Comparison of univariable analyses performed with Mann-Whitney U test and linear regression (Generalized Linear Model) in both MEX/CAM and individual cohorts. Associations between the expression of HLA class I alleles and 5 HIV clinical parameters (pVL, CD4 count, Z-score, CD4% and CD4/CD8 ratio) were investigated for alleles with frequency equal or greater than 5 in HIV-1 clade B-infected ART-naïve individuals from the pooled MEX/CAM (top table) and individuals cohorts (bottom tables). Associations were evaluated using the Mann-Whitney (MW) U test and linear regression (Generalized Linear Model, GLM) with multiple comparisons addresed with q-values. Significant (p<0.05, q<0.2) p and q-values are shown in bold. For GLM, coefficients and 95% confidence intervals are shown.

Pooled MEX/CAM (n=3213) univariable analysis

HIV outcome	Allele	N	HLA-HIV 5-parameter score (pVL, CD4, Z-score, %CD4, CD4/CD8)		HLA-HIV 2-parameter score (based only in pVL and CD4)		HLA associations using Mann-Whitney U test (●, p<0.05 & q<0.2)					HLA associations using Generalized Linear Model (●, p<0.05 & q<0.2)					Plasma Viral Load							Absolut CD4						
																	MW test		GLM					MW test		GLM				
			MW test	GLM	MW test	GLM	pVL	CD4	Z-score	%CD4	CD4/CD8	pVL	CD4	Z-score	%CD4	CD4/CD8	p-value	q-value	p-value	q-value	Coefficient	95% CI		p-value	q-value	p-value	q-value	Coefficient	95% CI	
HLA-HIV PROTECTIVE associations	A*02:05	71	4	3	1	1		●	●	●	●		●	●	●		9.5E-02	2.7E-01	7.1E-02	2.5E-01	-0.215	-0.449	0.018	**3.0E-04**	**5.8E-03**	**1.0E-05**	**1.6E-04**	150.229	84.391	216.067
	A*03:01	299	4	3	1	1		●	●	●	●		●	●	●		9.2E-02	2.7E-01	8.7E-02	2.8E-01	-0.105	-0.225	0.015	**1.0E-05**	**3.2E-04**	**1.0E-05**	**1.6E-04**	73.498	39.441	107.555
	A*11:01	202	4	4	1	1		●	●	●	●		●	●	●	●	5.3E-02	**2.0E-01**	1.6E-01	3.7E-01	-0.104	-0.248	0.040	**3.0E-02**	**1.4E-01**	**4.0E-03**	**3.6E-02**	59.250	18.709	99.791
	A*25:01	44	3	3	1	1		●		●	●		●		●	●	3.7E-01	5.0E-01	5.3E-01	6.2E-01	-0.095	-0.390	0.201	**2.5E-02**	**1.3E-01**	**1.8E-02**	**1.1E-01**	100.271	17.389	183.153
	A*26:01	143	2	2	1	1		●	●				●	●			6.1E-02	2.1E-01	6.6E-02	2.4E-01	-0.157	-0.324	0.010	**2.2E-02**	**1.2E-01**	**3.0E-03**	**2.9E-02**	70.307	23.448	117.166
	A*30:02	171	2	2	1	1	●		●			●		●			**1.0E-04**	**2.3E-03**	**1.0E-05**	**1.6E-04**	-0.356	-0.509	-0.203	5.1E-02	**2.0E-01**	8.4E-02	2.7E-01	38.079	-5.107	81.264
	A*80:01	8	0	3	0	0								●	●	●	2.1E-01	4.0E-01	**4.7E-02**	2.0E-01	-0.697	-1.385	-0.008	2.1E-01	4.0E-01	1.1E-01	3.1E-01	158.945	-34.409	352.298
	B*07:02	301	1	0	1	0		●									5.1E-01	5.7E-01	4.3E-01	5.7E-01	-0.049	-0.172	0.074	**3.3E-02**	**1.5E-01**	6.1E-02	2.3E-01	33.283	-1.597	68.163
	B*14:02	189	5	5	2	2	●	●	●	●	●	●	●	●	●	●	**3.0E-03**	**3.1E-02**	**3.0E-03**	**2.9E-02**	-0.228	-0.377	-0.079	**3.0E-04**	**5.8E-03**	**1.0E-05**	**1.6E-04**	100.908	59.108	142.708
	B*15:10	52	0	1	0	1						●					5.1E-02	**1.9E-01**	**8.0E-03**	**6.2E-02**	-0.371	-0.646	-0.097	7.0E-01	6.4E-01	7.9E-01	7.0E-01	10.487	-66.662	87.636
	B*15:39	8	2	5	1	2	●		●			●	●	●	●	●	**5.2E-03**	**4.7E-02**	**1.0E-05**	**1.6E-04**	-1.241	-1.929	-0.552	9.1E-02	2.7E-01	**2.7E-02**	**1.4E-01**	217.571	24.266	410.877
	B*27:05	65	5	5	2	2	●	●	●	●	●	●	●	●	●	●	**4.0E-04**	**7.1E-03**	**1.0E-05**	**1.6E-04**	-0.516	-0.763	-0.269	**1.0E-05**	**3.2E-04**	**1.0E-05**	**1.6E-04**	201.568	132.376	270.759
	B*39:02	98	2	2	0	0				●	●				●	●	1.9E-01	3.8E-01	1.8E-01	3.9E-01	-0.139	-0.340	0.062	2.6E-01	4.4E-01	5.7E-01	6.3E-01	16.413	-40.517	73.343
	B*40:05	18	2	1	1	1		●		●			●				6.8E-01	6.3E-01	5.3E-01	6.2E-01	0.147	-0.313	0.608	**9.1E-03**	**6.8E-02**	**3.4E-02**	**1.7E-01**	139.393	10.309	268.477
	B*40:06	8	2	2	1	1		●	●				●	●			1.1E-01	3.0E-01	9.6E-02	2.9E-01	-0.627	-1.363	0.110	**1.4E-02**	**9.1E-02**	**6.0E-03**	**5.0E-02**	288.136	81.602	494.670
	B*42:01	42	4	5	1	2		●	●	●	●	●	●	●	●	●	5.6E-02	2.0E-01	**4.2E-02**	**1.9E-01**	-0.318	-0.624	-0.012	**3.1E-02**	**1.5E-01**	**2.7E-02**	**1.4E-01**	98.028	11.135	184.920
	B*44:03	221	1	1	0	0					●					●	6.5E-01	6.2E-01	6.1E-01	6.5E-01	-0.036	-0.175	0.103	3.7E-01	5.0E-01	3.1E-01	4.9E-01	20.440	-18.938	59.819
	B*50:01	40	0	1	0	1							●				4.7E-01	5.5E-01	3.5E-01	5.1E-01	-0.149	-0.459	0.161	7.5E-02	2.4E-01	**2.7E-02**	**1.4E-01**	98.365	11.473	185.257
	B*57:01	61	5	5	2	2	●	●	●	●	●	●	●	●	●	●	**1.0E-04**	**2.3E-03**	**1.0E-05**	**1.6E-04**	-0.644	-0.906	-0.382	**1.7E-03**	**2.1E-02**	**1.0E-05**	**1.6E-04**	142.792	68.620	216.963
	B*57:02	7	2	2	0	0				●	●				●	●	2.4E-01	4.3E-01	1.6E-01	3.8E-01	-0.525	-1.262	0.212	1.1E-01	2.9E-01	7.3E-02	2.5E-01	188.916	-17.758	395.590
	B*57:03	38	5	5	2	2	●	●	●	●	●	●	●	●	●	●	**1.0E-05**	**3.2E-04**	**1.0E-05**	**1.6E-04**	-0.895	-1.229	-0.560	**3.4E-03**	**3.4E-02**	**1.0E-05**	**1.6E-04**	180.544	85.120	275.968
	B*58:01	85	2	0	1	0	●		●								**3.5E-02**	**1.6E-01**	5.6E-02	2.3E-01	-0.211	-0.428	0.005	6.1E-02	2.1E-01	1.2E-01	3.2E-01	48.329	-12.437	109.096
	B*81:01	16	2	0	1	0	●		●								**1.9E-02**	**1.1E-01**	**4.9E-02**	2.1E-01	-0.489	-0.977	-0.001	1.7E-01	3.7E-01	4.1E-01	5.5E-01	57.736	-79.218	194.689
	C*02:02	162	5	5	2	2	●	●	●	●	●	●	●	●	●	●	**2.0E-02**	**1.1E-01**	**1.2E-02**	**8.2E-02**	-0.205	-0.365	-0.045	**3.4E-02**	**1.5E-01**	**3.3E-02**	**1.6E-01**	48.902	3.883	93.921
	C*03:02	71	1	0	1	0	●										**4.5E-02**	**1.8E-01**	9.4E-02	2.9E-01	-0.201	-0.437	0.034	1.6E-01	3.6E-01	4.2E-01	5.6E-01	27.609	-38.960	94.178
	C*03:05	222	1	1	1	1	●					●					**4.2E-02**	**1.7E-01**	**3.5E-02**	**1.7E-01**	-0.150	-0.289	-0.011	5.1E-01	5.7E-01	4.3E-01	5.7E-01	-15.664	-54.811	23.483
	C*08:01	160	1	2	0	0				●					●	●	2.3E-01	4.2E-01	4.6E-01	5.8E-01	-0.060	-0.219	0.099	3.0E-01	4.6E-01	2.2E-01	4.3E-01	28.129	-16.775	73.032
	C*08:02	246	5	4	2	2	●	●	●	●	●	●	●	●	●		**2.1E-03**	**2.4E-02**	**3.0E-03**	**2.9E-02**	-0.198	-0.329	-0.066	**1.0E-04**	**2.3E-03**	**1.0E-05**	**1.6E-04**	90.895	53.873	127.918
	C*12:02	67	1	1	1	1	●					●					**2.8E-02**	**1.4E-01**	**3.6E-02**	**1.7E-01**	-0.260	-0.502	-0.017	4.8E-01	5.5E-01	3.4E-01	5.1E-01	33.420	-34.609	101.450
	C*12:03	189	3	3	1	1		●	●	●			●	●	●		8.8E-02	2.6E-01	5.5E-02	2.2E-01	-0.145	-0.293	0.003	**2.4E-03**	**2.7E-02**	**1.0E-03**	**1.2E-02**	71.070	29.487	112.652
	C*14:02	55	3	3	2	2	●	●	●			●	●	●			**1.7E-02**	**1.0E-01**	**2.8E-02**	**1.5E-01**	-0.296	-0.561	-0.031	**1.4E-02**	**9.1E-02**	**6.0E-03**	**5.0E-02**	103.269	28.956	177.583
	C*14:03	6	1	2	1	1	●					●		●			**2.3E-02**	**1.3E-01**	**1.7E-02**	**1.0E-01**	-0.970	-1.766	-0.174	2.9E-01	4.6E-01	1.7E-01	3.8E-01	157.495	-65.940	380.930
	C*17:01	106	3	2	1	1		●		●	●		●		●		6.2E-01	6.1E-01	4.0E-01	5.5E-01	-0.082	-0.276	0.111	**3.2E-02**	**1.5E-01**	**3.1E-02**	**1.6E-01**	59.912	5.414	114.410
	C*18:01	37	5	5	2	2	●	●	●	●	●	●	●	●	●	●	**1.0E-05**	**3.2E-04**	**1.0E-05**	**1.6E-04**	-0.775	-1.096	-0.454	**4.0E-04**	**7.1E-03**	**1.0E-05**	**1.6E-04**	170.966	79.479	262.454
HLA-HIV RISK associations	A*01:02	8	-1	-1	-1	-1	●					●					**1.4E-02**	**9.1E-02**	**2.2E-02**	**1.2E-01**	0.801	0.113	1.489	4.2E-01	5.2E-01	4.4E-01	5.8E-01	-76.012	-269.427	117.404
	A*02:01	1278	-2	-2	-1	-1		●			●		●	●			3.0E-01	4.6E-01	1.4E-01	3.5E-01	0.054	-0.017	0.126	**2.1E-02**	**1.2E-01**	**9.0E-03**	**6.6E-02**	-27.193	-47.460	-6.926
	A*02:06	441	-1	-2	-1	-1		●					●			●	4.8E-01	5.6E-01	8.7E-01	7.1E-01	-0.009	-0.110	0.093	**3.9E-02**	**1.7E-01**	**6.0E-03**	**5.0E-02**	-40.022	-68.801	-11.243
	A*24:02	966	-2	-2	-1	-1	●		●			●		●			**2.4E-03**	**2.7E-02**	**1.3E-02**	**8.5E-02**	0.097	0.021	0.174	1.7E-01	3.7E-01	6.7E-02	2.5E-01	-20.214	-41.837	1.409
	A*68:01	382	-5	-5	-2	-2	●	●	●	●	●	●	●	●	●	●	**9.0E-04**	**1.3E-02**	**2.0E-03**	**2.1E-02**	0.168	0.061	0.276	**7.7E-03**	**6.1E-02**	**1.0E-02**	**7.2E-02**	-40.226	-70.682	-9.770
	A*68:03	370	-5	-5	-2	-2	●	●	●	●	●	●	●	●	●	●	**7.9E-03**	**6.2E-02**	**1.1E-02**	**7.7E-02**	0.142	0.032	0.252	**1.0E-05**	**3.2E-04**	**1.0E-05**	**1.6E-04**	-63.002	-93.901	-32.103
	A*68:05	60	-2	0	0	0				●	●						3.7E-01	5.0E-01	4.4E-01	5.8E-01	0.100	-0.156	0.355	2.7E-01	4.5E-01	4.5E-01	5.8E-01	-27.538	-99.344	44.268
	B*13:02	65	-1	0	0	0					●						1.2E-01	3.1E-01	1.7E-01	3.8E-01	0.174	-0.072	0.420	1.0E-01	2.8E-01	2.4E-01	4.4E-01	-41.254	-110.254	27.746
	B*15:30	20	-3	-1	-1	0		●		●	●				●		8.5E-01	6.9E-01	8.2E-01	7.1E-01	0.051	-0.386	0.488	**4.0E-02**	**1.7E-01**	1.1E-01	3.1E-01	-102.051	-227.751	23.649
	B*35:01	645	-4	-4	-2	-1	●	●	●	●			●	●	●	●	**4.1E-02**	**1.7E-01**	6.6E-02	2.4E-01	0.084	-0.006	0.174	**1.8E-02**	**1.1E-01**	**2.0E-02**	**1.2E-01**	-29.975	-55.298	-4.652
	B*35:02	51	-2	-3	-1	-1	●		●			●		●		●	**2.1E-03**	**2.4E-02**	**1.0E-03**	**1.2E-02**	0.462	0.182	0.742	8.8E-02	2.6E-01	**4.8E-02**	2.0E-01	-79.423	-158.057	-0.789
	B*35:12	273	-5	-4	-2	-2	●	●	●	●	●	●	●	●	●		**2.0E-04**	**4.1E-03**	**1.0E-03**	**1.2E-02**	0.216	0.090	0.343	**1.0E-04**	**2.3E-03**	**1.0E-05**	**1.6E-04**	-67.726	-103.669	-31.782
	B*35:14	38	-1	-1	0	0			●					●			7.6E-02	2.4E-01	9.4E-02	2.9E-01	0.275	-0.047	0.597	1.3E-01	3.2E-01	1.3E-01	3.4E-01	-71.364	-162.935	20.207
	B*39:01	127	-1	-1	-1	-1		●					●				4.0E-01	5.1E-01	2.3E-01	4.3E-01	0.113	-0.069	0.295	**4.1E-02**	**1.7E-01**	**3.5E-02**	**1.7E-01**	-55.643	-107.307	-3.979
	B*39:05	360	-5	-5	-2	-2	●	●	●	●	●	●	●	●	●	●	**2.6E-03**	**2.8E-02**	**3.0E-03**	**2.9E-02**	0.172	0.059	0.285	**1.0E-05**	**3.2E-04**	**1.0E-05**	**1.6E-04**	-71.364	-103.364	-39.363
	B*39:06	153	-2	-2	-1	-1	●		●			●		●			**1.1E-02**	**7.8E-02**	**1.1E-02**	**7.7E-02**	0.221	0.051	0.390	1.5E-01	3.4E-01	1.0E-01	3.0E-01	-39.763	-87.317	7.790
	B*40:01	46	-2	-1	0	0				●	●				●		1.8E-01	3.7E-01	9.7E-02	2.9E-01	0.247	-0.045	0.540	8.1E-02	2.5E-01	1.6E-01	3.7E-01	-59.261	-141.288	22.765
	C*03:04	427	-1	0	-1	0	●										**3.4E-02**	**1.5E-01**	6.3E-02	2.4E-01	0.099	-0.005	0.203	2.4E-01	4.3E-01	2.1E-01	4.2E-01	-18.740	-48.186	10.706
	C*04:01	1237	-3	-3	-2	-2	●	●	●			●	●	●			**1.0E-05**	**3.2E-04**	**1.0E-05**	**1.6E-04**	0.155	0.082	0.227	**1.0E-02**	**7.3E-02**	**3.0E-03**	**2.9E-02**	-30.632	-51.094	-10.169
	C*07:02	1171	-5	-5	-2	-2	●	●	●	●	●	●	●	●	●	●	**9.0E-04**	**1.3E-02**	**1.0E-03**	**1.2E-02**	0.131	0.057	0.204	**1.0E-05**	**3.2E-04**	**1.0E-05**	**1.6E-04**	-47.465	-68.266	-26.663

Allele	Z-score							Percentage CD4							CD4/CD8 ratio						
	MW test		GLM					MW test		GLM					MW test		GLM				
	p-value	q-value	p-value	q-value	Coefficient	95% CI		p-value	q-value	p-value	q-value	Coefficient	95% CI		p-value	q-value	p-value	q-value	Coefficient	95% CI	
A*02:05	**2.0E-03**	**2.4E-02**	**1.0E-05**	**1.6E-04**	0.386	0.179	0.592	**9.6E-03**	**7.0E-02**	**1.6E-02**	**1.0E-01**	2.921	0.543	5.299	**2.4E-02**	**1.3E-01**	1.7E-01	3.9E-01	0.057	-0.025	0.139
A*03:01	**2.0E-04**	**4.1E-03**	**1.0E-03**	**1.2E-02**	0.188	0.081	0.294	**9.1E-03**	**6.8E-02**	**9.0E-03**	**6.6E-02**	1.652	0.419	2.886	**8.0E-03**	**6.3E-02**	5.0E-02	2.1E-01	0.042	0.000	0.083
A*11:01	**1.3E-02**	**8.4E-02**	**1.6E-02**	**1.0E-01**	0.155	0.028	0.282	**8.5E-03**	**6.6E-02**	**9.0E-03**	**6.6E-02**	1.953	0.492	3.415	**6.8E-03**	**5.7E-02**	**3.5E-02**	**1.7E-01**	0.053	0.004	0.103
A*25:01	6.9E-02	2.3E-01	8.7E-02	2.8E-01	0.226	-0.033	0.486	**2.2E-02**	**1.2E-01**	**2.7E-02**	**1.4E-01**	3.367	0.380	6.355	**1.1E-02**	**7.8E-02**	**1.3E-02**	**8.5E-02**	0.127	0.027	0.228
A*26:01	**1.6E-02**	**9.8E-02**	**6.0E-03**	**5.0E-02**	0.204	0.057	0.351	7.1E-02	2.3E-01	1.1E-01	3.1E-01	1.386	-0.310	3.083	1.8E-01	3.7E-01	3.9E-01	5.4E-01	0.025	-0.032	0.082
A*30:02	**1.2E-03**	**1.6E-02**	**1.0E-05**	**1.6E-04**	0.247	0.112	0.382	4.2E-01	5.2E-01	2.6E-01	4.4E-01	0.909	-0.657	2.474	2.1E-01	4.0E-01	7.2E-02	2.5E-01	0.049	-0.004	0.102
A*80:01	1.9E-01	3.8E-01	**4.0E-02**	**1.8E-01**	0.635	0.030	1.240	1.0E-01	2.9E-01	**3.5E-02**	**1.7E-01**	7.496	0.530	14.463	8.5E-02	2.5E-01	**2.0E-03**	**2.1E-02**	0.375	0.140	0.610
B*07:02	1.2E-01	3.0E-01	1.4E-01	3.5E-01	0.083	-0.026	0.192	2.7E-01	4.5E-01	4.9E-01	6.0E-01	0.443	-0.819	1.705	5.2E-01	5.7E-01	9.7E-01	7.4E-01	-0.001	-0.044	0.042
B*14:02	**1.0E-04**	**2.3E-03**	**1.0E-05**	**1.6E-04**	0.298	0.167	0.429	**5.3E-03**	**4.7E-02**	**2.0E-03**	**2.1E-02**	2.414	0.896	3.931	**2.6E-02**	**1.3E-01**	**2.7E-02**	**1.4E-01**	0.058	0.007	0.110
B*15:10	2.1E-01	4.1E-01	9.7E-02	2.9E-01	0.205	-0.037	0.446	9.3E-01	7.1E-01	9.4E-01	7.3E-01	-0.110	-2.890	2.671	7.2E-01	6.4E-01	7.3E-01	6.8E-01	0.017	-0.078	0.112
B*15:39	**1.2E-02**	**7.9E-02**	**1.0E-03**	**1.2E-02**	1.014	0.409	1.619	1.5E-01	3.4E-01	**2.1E-02**	**1.2E-01**	8.191	1.225	15.158	1.0E-01	2.9E-01	**2.5E-02**	**1.4E-01**	0.270	0.035	0.505
B*27:05	**1.0E-05**	**3.2E-04**	**1.0E-05**	**1.6E-04**	0.620	0.404	0.837	**1.0E-05**	**3.2E-04**	**1.0E-05**	**1.6E-04**	7.479	4.986	9.972	**1.0E-05**	**3.2E-04**	**1.0E-05**	**1.6E-04**	0.232	0.148	0.316
B*39:02	1.5E-01	3.4E-01	3.2E-01	5.0E-01	0.090	-0.089	0.268	**2.7E-02**	**1.3E-01**	**2.1E-02**	**1.2E-01**	2.423	0.362	4.484	**1.7E-02**	**1.0E-01**	**5.0E-03**	**4.4E-02**	0.101	0.031	0.171
B*40:05	1.6E-01	3.5E-01	4.0E-01	5.5E-01	0.175	-0.230	0.579	**4.0E-02**	**1.7E-01**	7.8E-02	2.6E-01	4.190	-0.463	8.844	1.1E-01	2.9E-01	3.5E-01	5.1E-01	0.075	-0.082	0.232
B*40:06	**9.9E-03**	**7.1E-02**	**1.2E-02**	**8.2E-02**	0.831	0.184	1.478	5.9E-02	2.1E-01	1.1E-01	3.1E-01	6.142	-1.308	13.591	5.5E-02	2.0E-01	2.8E-01	4.7E-01	0.138	-0.114	0.389
B*42:01	**1.0E-02**	**7.4E-02**	**1.3E-02**	**8.5E-02**	0.346	0.074	0.618	**1.1E-03**	**1.5E-02**	**1.0E-03**	**1.2E-02**	5.555	2.427	8.683	**2.0E-04**	**4.1E-03**	**1.0E-05**	**1.6E-04**	0.204	0.097	0.311
B*44:03	3.9E-01	5.1E-01	3.1E-01	4.9E-01	0.064	-0.059	0.187	2.1E-01	4.0E-01	1.7E-01	3.8E-01	0.997	-0.425	2.419	**4.6E-02**	**1.8E-01**	**2.7E-02**	**1.4E-01**	0.054	0.006	0.102
B*50:01	1.7E-01	3.7E-01	7.1E-02	2.5E-01	0.251	-0.022	0.523	3.2E-01	4.7E-01	2.4E-01	4.4E-01	1.864	-1.270	4.997	3.6E-01	4.9E-01	1.7E-01	3.8E-01	0.076	-0.031	0.183
B*57:01	**1.0E-04**	**2.3E-03**	**1.0E-05**	**1.6E-04**	0.582	0.350	0.814	**1.0E-04**	**2.3E-03**	**1.0E-05**	**1.6E-04**	5.886	3.215	8.558	**1.0E-05**	**3.2E-04**	**1.0E-05**	**1.6E-04**	0.244	0.154	0.334
B*57:02	1.3E-01	3.2E-01	6.8E-02	2.5E-01	0.602	-0.045	1.249	**1.4E-02**	**9.1E-02**	**1.0E-05**	**1.6E-04**	13.301	5.863	20.739	**2.4E-02**	**1.3E-01**	**1.0E-05**	**1.6E-04**	0.805	0.535	1.075
B*57:03	**1.0E-05**	**3.2E-04**	**1.0E-05**	**1.6E-04**	0.779	0.481	1.077	**8.5E-03**	**6.6E-02**	**8.0E-03**	**6.2E-02**	4.683	1.240	8.126	**2.6E-03**	**2.8E-02**	**1.0E-05**	**1.6E-04**	0.224	0.108	0.340
B*58:01	**3.2E-02**	**1.5E-01**	**4.8E-02**	2.0E-01	0.192	0.002	0.382	1.3E-01	3.1E-01	2.3E-01	4.3E-01	1.336	-0.854	3.527	2.0E-01	3.9E-01	7.1E-01	6.8E-01	0.014	-0.060	0.089
B*81:01	**4.2E-02**	**1.7E-01**	1.1E-01	3.1E-01	0.349	-0.080	0.778	6.7E-01	6.2E-01	7.3E-01	6.9E-01	0.865	-4.072	5.801	3.3E-01	4.8E-01	6.8E-01	6.7E-01	0.035	-0.132	0.202
C*02:02	**1.6E-02**	**9.8E-02**	**8.0E-03**	**6.2E-02**	0.191	0.050	0.332	**1.2E-02**	**8.0E-02**	**9.0E-03**	**6.6E-02**	2.178	0.553	3.803	**1.7E-03**	**2.1E-02**	**3.0E-03**	**2.9E-02**	0.084	0.029	0.138
C*03:02	6.1E-02	2.1E-01	2.3E-01	4.3E-01	0.129	-0.079	0.338	3.7E-01	5.0E-01	5.3E-01	6.2E-01	0.766	-1.637	3.170	4.0E-01	5.2E-01	9.3E-01	7.3E-01	-0.004	-0.085	0.078
C*03:05	3.0E-01	4.6E-01	4.2E-01	5.6E-01	0.050	-0.072	0.173	5.8E-01	6.0E-01	4.2E-01	5.6E-01	-0.586	-2.000	0.827	5.2E-01	5.7E-01	7.0E-01	6.8E-01	-0.009	-0.057	0.038
C*08:01	2.4E-01	4.3E-01	2.8E-01	4.6E-01	0.078	-0.063	0.218	**2.6E-02**	**1.3E-01**	**1.0E-02**	**7.2E-02**	2.126	0.506	3.746	1.3E-01	3.2E-01	**2.3E-02**	**1.3E-01**	0.064	0.009	0.119
C*08:02	**1.0E-04**	**2.3E-03**	**1.0E-05**	**1.6E-04**	0.265	0.148	0.381	**4.7E-03**	**4.3E-02**	**2.0E-03**	**2.1E-02**	2.174	0.827	3.522	**4.9E-02**	**1.9E-01**	5.2E-02	2.1E-01	0.045	0.000	0.091
C*12:02	1.0E-01	2.9E-01	8.1E-02	2.7E-01	0.189	-0.024	0.402	2.4E-01	4.3E-01	2.1E-01	4.2E-01	1.555	-0.901	4.011	2.5E-01	4.3E-01	1.8E-01	3.9E-01	0.057	-0.026	0.140
C*12:03	**7.8E-03**	**6.2E-02**	**3.0E-03**	**2.9E-02**	0.201	0.070	0.331	**3.8E-02**	**1.7E-01**	**2.1E-02**	**1.2E-01**	1.769	0.267	3.272	1.0E-01	2.8E-01	6.0E-02	2.3E-01	0.049	-0.002	0.099
C*14:02	**4.6E-03**	**4.2E-02**	**5.0E-03**	**4.4E-02**	0.333	0.100	0.566	6.4E-02	2.2E-01	5.5E-02	2.2E-01	2.624	-0.061	5.308	1.2E-01	3.0E-01	1.6E-01	3.8E-01	0.065	-0.026	0.155
C*14:03	5.7E-02	2.1E-01	**3.1E-02**	**1.6E-01**	0.770	0.070	1.469	3.2E-01	4.7E-01	1.7E-01	3.9E-01	5.633	-2.434	13.700	2.9E-01	4.6E-01	1.3E-01	3.4E-01	0.211	-0.062	0.483
C*17:01	1.3E-01	3.1E-01	8.3E-02	2.7E-01	0.151	-0.020	0.322	**2.5E-02**	**1.3E-01**	**3.3E-02**	**1.6E-01**	2.141	0.173	4.109	**1.3E-02**	**8.6E-02**	6.5E-02	2.4E-01	0.063	-0.004	0.130
C*18:01	**1.0E-05**	**3.2E-04**	**1.0E-05**	**1.6E-04**	0.699	0.413	0.985	**3.4E-03**	**3.4E-02**	**1.0E-03**	**1.2E-02**	5.675	2.371	8.979	**5.0E-04**	**8.4E-03**	**1.0E-05**	**1.6E-04**	0.314	0.201	0.427
A*01:02	7.1E-02	2.3E-01	8.0E-02	2.7E-01	-0.541	-1.146	0.064	4.9E-01	5.6E-01	5.3E-01	6.2E-01	-2.262	-9.233	4.709	1.2E-01	3.0E-01	2.7E-01	4.5E-01	-0.143	-0.394	0.109
A*02:01	5.0E-02	**1.9E-01**	**1.8E-02**	**1.1E-01**	-0.077	-0.140	-0.013	1.3E-01	3.1E-01	1.5E-01	3.6E-01	-0.544	-1.276	0.189	**4.2E-02**	**1.7E-01**	1.2E-01	3.2E-01	-0.020	-0.045	0.005
A*02:06	4.7E-01	5.5E-01	1.5E-01	3.6E-01	-0.067	-0.157	0.023	2.9E-01	4.6E-01	1.9E-01	4.0E-01	-0.704	-1.743	0.336	1.2E-01	3.1E-01	**1.6E-02**	**1.0E-01**	-0.043	-0.078	-0.008
A*24:02	**1.6E-02**	**9.8E-02**	**1.6E-02**	**1.0E-01**	-0.083	-0.151	-0.015	6.7E-01	6.2E-01	9.5E-01	7.3E-01	0.024	-0.757	0.806	1.8E-01	3.7E-01	6.2E-01	6.5E-01	0.007	-0.020	0.033
A*68:01	**8.0E-04**	**1.2E-02**	**1.0E-03**	**1.2E-02**	-0.163	-0.258	-0.068	**2.0E-02**	**1.2E-01**	**1.6E-02**	**1.0E-01**	-1.349	-2.447	-0.251	**9.3E-03**	**6.9E-02**	**4.3E-02**	**1.9E-01**	-0.039	-0.076	-0.001
A*68:03	**1.0E-04**	**2.3E-03**	**1.0E-05**	**1.6E-04**	-0.183	-0.280	-0.086	**1.0E-05**	**3.2E-04**	**1.0E-05**	**1.6E-04**	-2.789	-3.904	-1.673	**1.0E-05**	**3.2E-04**	**1.0E-05**	**1.6E-04**	-0.088	-0.126	-0.050
A*68:05	2.9E-01	4.6E-01	3.8E-01	5.3E-01	-0.101	-0.325	0.124	**3.8E-02**	**1.6E-01**	6.0E-02	2.3E-01	-2.503	-5.112	0.105	**2.5E-02**	**1.3E-01**	2.0E-01	4.1E-01	-0.058	-0.147	0.031
B*13:02	7.7E-02	2.4E-01	1.4E-01	3.5E-01	-0.163	-0.379	0.053	5.8E-02	2.1E-01	1.1E-01	3.1E-01	-2.041	-4.527	0.446	**4.4E-02**	**1.8E-01**	1.5E-01	3.6E-01	-0.062	-0.146	0.022
B*15:30	1.7E-01	3.6E-01	2.5E-01	4.4E-01	-0.233	-0.627	0.161	**1.1E-02**	**7.8E-02**	**1.4E-02**	**9.1E-02**	-5.689	-10.216	-1.161	**1.2E-02**	**8.2E-02**	6.6E-02	2.4E-01	-0.147	-0.304	0.010
B*35:01	**2.4E-02**	**1.3E-01**	**1.7E-02**	**1.0E-01**	-0.096	-0.176	-0.017	**4.3E-02**	**1.8E-01**	**2.2E-02**	**1.2E-01**	-1.066	-1.980	-0.151	2.7E-01	4.5E-01	**3.8E-02**	**1.8E-01**	-0.033	-0.064	-0.002
B*35:02	**4.8E-03**	**4.3E-02**	**3.0E-03**	**2.9E-02**	-0.376	-0.622	-0.130	1.4E-01	3.3E-01	8.3E-02	2.7E-01	-2.506	-5.341	0.328	6.3E-02	2.2E-01	**3.1E-02**	**1.6E-01**	-0.105	-0.201	-0.010
B*35:12	**1.0E-05**	**3.2E-04**	**1.0E-05**	**1.6E-04**	-0.232	-0.345	-0.120	**9.4E-03**	**6.9E-02**	**4.5E-02**	**2.0E-01**	-1.327	-2.624	-0.029	**7.3E-03**	**6.0E-02**	3.7E-01	5.3E-01	-0.020	-0.064	0.024
B*35:14	**3.8E-02**	**1.7E-01**	**4.1E-02**	**1.9E-01**	-0.299	-0.585	-0.012	8.0E-01	6.7E-01	6.8E-01	6.7E-01	-0.709	-4.057	2.638	4.5E-01	5.4E-01	2.5E-01	4.4E-01	-0.067	-0.180	0.046
B*39:01	1.3E-01	3.2E-01	6.1E-02	2.3E-01	-0.155	-0.316	0.007	9.7E-02	2.8E-01	9.2E-02	2.9E-01	-1.607	-3.477	0.263	1.2E-01	3.0E-01	1.3E-01	3.4E-01	-0.049	-0.112	0.015
B*39:05	**1.0E-05**	**3.2E-04**	**1.0E-05**	**1.6E-04**	-0.222	-0.323	-0.122	**1.0E-05**	**3.2E-04**	**1.0E-05**	**1.6E-04**	-2.396	-3.551	-1.240	**1.0E-05**	**3.2E-04**	**1.0E-03**	**1.2E-02**	-0.064	-0.104	-0.025
B*39:06	**2.0E-02**	**1.1E-01**	**1.6E-02**	**1.0E-01**	-0.184	-0.333	-0.035	8.1E-01	6.7E-01	9.7E-01	7.4E-01	-0.031	-1.751	1.690	9.9E-01	7.2E-01	4.6E-01	5.8E-01	-0.022	-0.080	0.036
B*40:01	7.4E-02	2.3E-01	7.7E-02	2.6E-01	-0.232	-0.489	0.025	**1.4E-02**	**9.1E-02**	**3.3E-02**	**1.6E-01**	-3.224	-6.179	-0.269	**1.5E-02**	**9.3E-02**	1.1E-01	3.1E-01	-0.081	-0.181	0.018
C*03:04	9.3E-02	2.7E-01	1.0E-01	3.0E-01	-0.077	-0.169	0.015	1.7E-01	3.6E-01	1.1E-01	3.1E-01	-0.865	-1.928	0.199	1.8E-01	3.8E-01	1.7E-01	3.9E-01	-0.025	-0.061	0.011
C*04:01	**1.0E-04**	**2.3E-03**	**1.0E-05**	**1.6E-04**	-0.136	-0.200	-0.072	1.2E-01	3.0E-01	1.5E-01	3.6E-01	-0.545	-1.285	0.196	1.4E-01	3.2E-01	2.1E-01	4.2E-01	-0.016	-0.041	0.009
C*07:02	**1.0E-05**	**3.2E-04**	**1.0E-05**	**1.6E-04**	-0.155	-0.220	-0.090	**2.0E-04**	**4.1E-03**	**1.0E-05**	**1.6E-04**	-1.479	-2.233	-0.726	**1.0E-05**	**3.2E-04**	**1.0E-05**	**1.6E-04**	-0.046	-0.071	-0.020

Only MEX (n=1679) univariable analysis

HIV outcome	Allele	N	HLA-HIV 5-parameter score (pVL, CD4, Z-score, %CD4, CD4/CD8)		HLA-HIV 2-parameter score (based only in pVL and CD4)		HLA associations using Mann-Whitney U test (●, p<0.05 & q<0.2)					HLA associations using Generalized Linear Model (●, p<0.05 & q<0.2)					Plasma Viral Load							Absolut CD4						
																	MW test		GLM					MW test		GLM				
			MW test	GLM	MW test	GLM	pVL	CD4	Z-score	%CD4	CD4/CD8	pVL	CD4	Z-score	%CD4	CD4/CD8	p-value	q-value	p-value	q-value	Coefficient	95% CI		p-value	q-value	p-value	q-value	Coefficient	95% CI	
ons	A*03:01	137	3	3	1	1		●	●	●			●	●	●		1.8E-01	4.6E-01	3.0E-01	4.9E-01	-0.088	-0.252	0.077	**1.1E-03**	**3.0E-02**	**1.0E-03**	**1.6E-02**	83.495	32.733	134.256
	A*03:02	5	0	1	0	0									●		4.4E-01	6.1E-01	5.1E-01	5.8E-01	-0.271	-1.085	0.544	1.6E-01	4.3E-01	3.0E-01	5.0E-01	128.832	-116.173	373.837
	A*26:01	62	2	2	1	1		●	●				●	●			1.0E-01	3.6E-01	7.9E-02	2.8E-01	-0.211	-0.446	0.024	**1.3E-02**	**1.2E-01**	**1.0E-03**	**1.6E-02**	122.209	51.611	192.807
	A*31:01	223	0	1	0	1						●					6.4E-02	2.9E-01	**2.1E-02**	**1.4E-01**	-0.155	-0.288	-0.023	6.3E-01	6.7E-01	4.1E-01	5.4E-01	16.982	-23.285	57.250
	A*36:01	5	0	1	0	0										●	8.2E-01	7.2E-01	7.4E-01	6.5E-01	-0.136	-0.950	0.679	7.0E-01	6.9E-01	4.6E-01	5.6E-01	93.225	-151.818	338.268
	A*74:01	16	0	2	0	1						●		●			2.6E-01	5.2E-01	**2.1E-02**	**1.4E-01**	-0.535	-0.991	-0.079	1.9E-01	4.7E-01	6.5E-02	2.7E-01	129.425	-7.902	266.751

Allele	Z-score							Percentage CD4							CD4/CD8 ratio						
	MW test		GLM					MW test		GLM					MW test		GLM				
	p-value	q-value	p-value	q-value	Coefficient	95% CI		p-value	q-value	p-value	q-value	Coefficient	95% CI		p-value	q-value	p-value	q-value	Coefficient	95% CI	
A*03:01	**5.4E-03**	**7.2E-02**	**9.0E-03**	**7.6E-02**	0.204	0.050	0.358	**1.3E-02**	**1.2E-01**	**1.3E-02**	**9.7E-02**	2.374	0.495	4.253	**3.4E-02**	2.1E-01	1.3E-01	3.6E-01	0.048	-0.014	0.111
A*03:02	2.5E-01	5.1E-01	3.3E-01	5.1E-01	0.368	-0.375	1.110	8.4E-02	3.3E-01	**3.7E-02**	**2.0E-01**	9.536	0.556	18.515	1.1E-01	3.7E-01	8.2E-02	2.9E-01	0.265	-0.033	0.564
A*26:01	**2.3E-02**	**1.7E-01**	**3.0E-03**	**3.5E-02**	0.326	0.112	0.540	1.0E-01	3.6E-01	1.2E-01	3.4E-01	2.085	-0.533	4.703	4.1E-01	5.9E-01	4.8E-01	5.7E-01	0.032	-0.056	0.120
A*31:01	1.9E-01	4.7E-01	7.9E-02	2.8E-01	0.109	-0.013	0.231	8.1E-01	7.2E-01	8.1E-01	6.6E-01	0.183	-1.296	1.661	9.4E-01	7.5E-01	1.9E-01	4.2E-01	0.033	-0.016	0.082
A*36:01	9.9E-01	7.6E-01	5.3E-01	5.9E-01	0.236	-0.507	0.978	4.0E-01	5.8E-01	2.2E-01	4.4E-01	5.680	-3.308	14.667	4.9E-01	6.3E-01	**1.0E-03**	**1.6E-02**	0.506	0.208	0.804
A*74:01	2.5E-01	5.1E-01	**1.8E-02**	**1.2E-01**	0.502	0.086	0.918	7.9E-01	7.2E-01	5.0E-01	5.8E-01	1.723	-3.319	6.766	8.1E-01	7.2E-01	3.4E-01	5.1E-01	0.082	-0.086	0.249

HLA-HIV PROTECTIVE associati	B*14:02	101	5	5	2	2	•	•	•	•	•	•	•	•	•	•	5.0E-04	1.8E-02	1.0E-03	1.6E-02	-0.327	-0.517	-0.137	1.6E-03	3.6E-02	1.0E-05	2.2E-04	120.245	62.994	177.496	1.0E-04	5.4E-03	1.0E-05	2.2E-04	0.387	0.214	0.561	8.0E-04	2.5E-02	1.0E-05	2.2E-04	3.875	1.750	6.000	3.3E-03	5.8E-02	7.0E-03	6.2E-02	0.099	0.027	0.170
	B*15:17	8	0	1	0	0										•	7.2E-01	7.0E-01	9.5E-01	7.0E-01	0.022	-0.624	0.668	2.2E-01	5.0E-01	2.7E-01	4.7E-01	109.553	-84.270	303.377	3.9E-01	5.7E-01	5.4E-01	5.9E-01	0.186	-0.402	0.774	2.3E-01	5.1E-01	1.4E-01	3.7E-01	5.357	-1.754	12.467	6.3E-01	6.7E-01	6.0E-03	5.8E-02	0.382	0.110	0.655
	B*15:39	6	5	5	2	2	•	•	•	•	•	•	•	•	•	•	2.8E-03	5.2E-02	1.0E-05	2.2E-04	-1.529	-2.270	-0.787	2.6E-02	1.8E-01	4.0E-03	4.4E-02	325.452	102.254	548.650	4.3E-03	6.3E-02	1.0E-05	2.2E-04	1.354	0.678	2.029	2.4E-02	1.7E-01	2.0E-03	2.5E-02	12.954	4.768	21.140	1.3E-02	1.2E-01	2.0E-03	2.5E-02	0.429	0.157	0.702
	B*27:05	46	5	5	2	2	•	•	•	•	•	•	•	•	•	•	3.0E-04	1.3E-02	1.0E-05	2.2E-04	-0.646	-0.922	-0.369	1.0E-05	9.3E-04	1.0E-05	2.2E-04	262.225	179.591	344.858	1.0E-05	9.3E-04	1.0E-05	2.2E-04	0.796	0.545	1.046	1.0E-05	9.3E-04	1.0E-05	2.2E-04	9.234	6.199	12.269	1.0E-05	9.3E-04	1.0E-05	2.2E-04	0.285	0.184	0.386
	B*39:02	68	2	3	1	1	•				•	•			•	•	9.9E-03	1.1E-01	6.0E-03	5.8E-02	-0.319	-0.544	-0.094	2.8E-01	5.2E-01	4.9E-01	5.8E-01	24.018	-44.712	92.747	3.5E-02	2.1E-01	7.1E-02	2.8E-01	0.192	-0.016	0.400	3.4E-02	2.1E-01	2.6E-02	1.6E-01	2.874	0.336	5.411	2.6E-02	1.8E-01	1.0E-03	1.6E-02	0.140	0.056	0.225
	B*40:05	18	1	1	1	1		•					•				1.0E+00	7.6E-01	8.6E-01	6.7E-01	0.040	-0.392	0.472	1.1E-02	1.1E-01	3.7E-02	2.0E-01	138.038	8.548	267.527	9.6E-02	3.5E-01	2.6E-01	4.7E-01	0.228	-0.165	0.621	6.8E-02	2.9E-01	1.3E-01	3.6E-01	3.715	-1.040	8.470	1.0E-01	3.6E-01	3.4E-01	5.1E-01	0.077	-0.081	0.236
	B*51:02	18	0	1	0	0										•	6.3E-02	2.8E-01	5.9E-02	2.6E-01	-0.427	-0.871	0.017	1.1E-01	3.6E-01	2.2E-01	4.5E-01	83.090	-50.229	216.409	5.2E-02	2.6E-01	7.7E-02	2.8E-01	0.364	-0.040	0.768	2.0E-01	4.8E-01	1.8E-01	4.1E-01	3.323	-1.569	8.215	2.4E-01	5.1E-01	1.3E-02	9.7E-02	0.207	0.044	0.369
	B*57:01	31	4	5	1	2	•		•	•	•	•	•	•	•	•	7.0E-04	2.3E-02	1.0E-05	2.2E-04	-0.794	-1.127	-0.460	3.8E-02	2.2E-01	3.0E-03	3.5E-02	155.808	53.585	258.030	4.1E-03	6.3E-02	1.0E-05	2.2E-04	0.685	0.376	0.994	5.3E-03	7.2E-02	1.0E-03	1.6E-02	6.150	2.401	9.900	1.4E-03	3.3E-02	1.0E-05	2.2E-04	0.333	0.209	0.457
	B*57:03	17	2	4	1	2	•		•			•	•	•		•	2.5E-03	4.9E-02	2.0E-03	2.5E-02	-0.755	-1.226	-0.283	7.6E-02	3.1E-01	1.6E-02	1.2E-01	180.484	33.903	327.065	1.8E-02	1.5E-01	2.0E-03	2.5E-02	0.708	0.264	1.152	7.7E-02	3.1E-01	4.8E-02	2.3E-01	5.425	0.043	10.807	3.3E-02	2.1E-01	1.0E-05	2.2E-04	0.326	0.148	0.505
	C*02:02	74	5	5	2	2	•	•	•	•	•	•	•	•	•	•	3.2E-02	2.0E-01	5.0E-03	5.1E-02	-0.317	-0.537	-0.096	1.0E-02	1.1E-01	1.0E-03	1.6E-02	109.216	42.595	175.837	1.1E-02	1.1E-01	1.0E-03	1.6E-02	0.358	0.156	0.560	5.8E-03	7.5E-02	4.0E-03	4.4E-02	3.617	1.170	6.064	1.9E-03	3.9E-02	1.0E-03	1.6E-02	0.133	0.051	0.214
	C*03:03	121	2	2	1	1	•				•	•				•	7.7E-03	8.9E-02	1.9E-02	1.3E-01	-0.208	-0.381	-0.034	2.7E-01	5.2E-01	2.5E-01	4.6E-01	31.102	-21.361	83.564	6.4E-02	2.9E-01	4.9E-02	2.3E-01	0.160	0.001	0.318	6.2E-02	2.8E-01	4.1E-02	2.1E-01	2.020	0.080	3.960	2.9E-02	1.9E-01	2.6E-02	1.6E-01	0.074	0.009	0.139
	C*08:01	143	1	2	1	0	•								•	•	1.9E-02	1.5E-01	4.1E-02	2.1E-01	-0.167	-0.327	-0.007	3.2E-01	5.4E-01	2.4E-01	4.6E-01	29.248	-19.235	77.730	7.6E-02	3.1E-01	7.3E-02	2.8E-01	0.134	-0.013	0.281	5.1E-02	2.5E-01	2.2E-02	1.4E-01	2.076	0.298	3.854	6.3E-02	2.8E-01	8.0E-03	6.9E-02	0.081	0.022	0.140
	C*08:02	132	5	5	2	2	•	•	•	•	•	•	•	•	•	•	4.0E-04	1.6E-02	1.0E-03	1.6E-02	-0.278	-0.445	-0.111	9.0E-04	2.7E-02	1.0E-05	2.2E-04	103.801	53.139	154.463	1.0E-04	5.4E-03	1.0E-05	2.2E-04	0.331	0.178	0.485	5.0E-04	1.8E-02	1.0E-05	2.2E-04	3.489	1.607	5.372	3.7E-03	6.3E-02	1.0E-02	8.1E-02	0.083	0.020	0.146
	C*14:02	25	5	5	2	2	•	•	•	•	•	•	•	•	•	•	1.9E-02	1.5E-01	8.0E-03	6.9E-02	-0.500	-0.866	-0.133	1.7E-03	3.7E-02	2.0E-03	2.5E-02	177.244	67.317	287.171	1.4E-03	3.3E-02	1.0E-03	1.6E-02	0.569	0.236	0.903	7.9E-03	8.9E-02	5.0E-03	5.1E-02	5.732	1.695	9.770	1.7E-02	1.4E-01	2.6E-02	1.6E-01	0.153	0.019	0.288
	C*18:01	8	0	1	0	0										•	5.5E-01	6.5E-01	3.8E-01	5.3E-01	-0.289	-0.934	0.357	2.5E-01	5.1E-01	1.8E-01	4.1E-01	140.611	-66.501	347.724	4.9E-01	6.3E-01	2.4E-01	4.6E-01	0.373	-0.255	1.001	1.7E-01	4.4E-01	7.0E-02	2.8E-01	7.036	-0.562	14.635	9.0E-02	3.4E-01	1.0E-05	2.2E-04	0.522	0.271	0.774
HLA-HIV RISK associations	A*23:01	59	-1	-1	-1	-1	•					•					8.4E-03	9.3E-02	1.1E-02	8.7E-02	0.315	0.072	0.557	8.1E-01	7.2E-01	5.7E-01	5.9E-01	-21.347	-94.495	51.801	1.3E-01	4.0E-01	8.2E-02	2.9E-01	-0.197	-0.418	0.025	5.6E-01	6.5E-01	5.6E-01	5.9E-01	0.808	-1.876	3.492	6.1E-01	6.6E-01	7.6E-01	6.5E-01	0.014	-0.075	0.103
	A*24:03	6	-1	-1	-1	-1	•					•					2.6E-02	1.8E-01	2.0E-02	1.3E-01	0.880	0.138	1.623	3.7E-01	5.7E-01	4.5E-01	5.6E-01	85.759	-138.001	309.520	5.9E-01	6.6E-01	4.0E-01	5.4E-01	-0.289	-0.967	0.389	5.3E-01	6.5E-01	6.7E-01	6.3E-01	1.813	-6.397	10.024	6.7E-01	6.8E-01	8.9E-01	6.8E-01	0.019	-0.254	0.293
	A*68:03	175	-3	-4	-1	-1		•		•	•		•	•	•	•	2.9E-01	5.2E-01	2.2E-01	4.5E-01	0.091	-0.056	0.238	1.8E-02	1.5E-01	7.0E-03	6.2E-02	-61.486	-106.067	-16.906	4.5E-02	2.4E-01	2.8E-02	1.7E-01	-0.151	-0.286	-0.016	1.3E-03	3.2E-02	2.0E-03	2.5E-02	-2.651	-4.295	-1.008	3.0E-04	1.3E-02	1.0E-03	1.6E-02	-0.090	-0.145	-0.035
	B*15:30	17	-2	-1	0	0				•	•				•		7.9E-01	7.2E-01	7.2E-01	6.4E-01	-0.081	-0.526	0.363	3.3E-02	2.1E-01	1.1E-01	3.3E-01	-111.505	-248.841	25.831	2.5E-01	5.1E-01	3.8E-01	5.3E-01	-0.186	-0.603	0.231	1.5E-02	1.3E-01	1.7E-02	1.2E-01	-6.139	-11.174	-1.105	2.9E-02	1.9E-01	1.2E-01	3.4E-01	-0.135	-0.303	0.033
	B*35:12	159	-3	-2	-1	-1		•		•	•		•		•		3.0E-01	5.3E-01	5.1E-01	5.8E-01	0.052	-0.104	0.208	4.8E-03	6.6E-02	5.0E-03	5.1E-02	-68.411	-115.882	-20.941	3.6E-02	2.1E-01	5.0E-02	2.4E-01	-0.144	-0.288	0.000	1.1E-02	1.1E-01	3.6E-02	2.0E-01	-1.871	-3.615	-0.126	9.6E-03	1.0E-01	4.1E-01	5.5E-01	-0.025	-0.083	0.034
	B*37:01	11	-1	-1	-1	-1	•					•					2.1E-02	1.5E-01	2.4E-02	1.5E-01	0.632	0.081	1.182	3.4E-01	5.6E-01	3.2E-01	5.1E-01	-83.677	-249.134	81.781	6.1E-02	2.8E-01	6.7E-02	2.7E-01	-0.468	-0.969	0.033	1.4E-01	4.2E-01	1.6E-01	3.9E-01	-4.311	-10.380	1.759	1.3E-01	4.0E-01	1.7E-01	4.0E-01	-0.143	-0.345	0.059
	B*39:05	246	-4	-4	-1	-1		•	•	•	•		•	•	•	•	6.1E-02	2.8E-01	7.8E-02	2.8E-01	0.117	-0.013	0.248	1.0E-03	2.8E-02	3.0E-03	3.5E-02	-60.630	-100.086	-21.175	1.7E-03	3.7E-02	4.0E-03	4.4E-02	-0.178	-0.298	-0.059	1.0E-05	9.3E-04	1.0E-05	2.2E-04	-2.843	-4.292	-1.395	1.0E-05	9.3E-04	2.0E-03	2.5E-02	-0.078	-0.127	-0.029
	C*04:01	668	-2	-2	-1	-1		•	•				•	•			6.9E-02	3.0E-01	7.5E-02	2.8E-01	0.085	-0.008	0.178	7.9E-03	8.9E-02	6.0E-03	5.8E-02	-39.106	-67.250	-10.962	6.1E-03	7.6E-02	7.0E-03	6.2E-02	-0.117	-0.202	-0.032	4.5E-02	2.4E-01	6.5E-02	2.7E-01	-0.975	-2.012	0.062	3.9E-02	2.2E-01	1.7E-01	4.0E-01	-0.024	-0.059	0.010
	C*06:02	140	-2	-1	-1	-1	•		•			•					1.2E-02	1.2E-01	3.7E-02	2.0E-01	0.174	0.011	0.337	1.4E-01	4.2E-01	2.1E-01	4.4E-01	-31.845	-81.139	17.449	3.0E-02	1.9E-01	6.2E-02	2.7E-01	-0.142	-0.291	0.007	9.0E-02	3.4E-01	7.9E-02	2.8E-01	-1.621	-3.430	0.188	1.7E-01	4.4E-01	1.6E-01	3.9E-01	-0.044	-0.104	0.017
	C*07:02	666	-4	-5	-1	-2		•	•	•	•	•	•	•	•	•	4.5E-02	2.4E-01	2.3E-02	1.5E-01	0.108	0.015	0.201	4.0E-03	6.3E-02	1.0E-03	1.6E-02	-46.269	-74.466	-18.073	4.1E-03	6.3E-02	1.0E-03	1.6E-02	-0.142	-0.228	-0.057	8.0E-04	2.5E-02	1.0E-03	1.6E-02	-1.803	-2.841	-0.764	2.9E-03	5.3E-02	8.0E-03	6.9E-02	-0.047	-0.082	-0.012
	C*16:01	98	-2	-2	-1	-1		•	•				•	•			1.1E-01	3.6E-01	1.2E-01	3.5E-01	0.152	-0.041	0.345	3.1E-02	2.0E-01	1.8E-02	1.2E-01	-70.136	-128.448	-11.825	2.6E-02	1.8E-01	2.2E-02	1.4E-01	-0.207	-0.384	-0.030	7.0E-02	3.0E-01	5.0E-02	2.4E-01	-2.165	-4.329	-0.001	1.4E-01	4.2E-01	6.7E-02	2.7E-01	-0.067	-0.139	0.005

Only CAM (n=1534) univariable analysis

HIV outcome	Allele	N	HLA-HIV 5-parameter score (pVL, CD4, Z-score, %CD4, CD4/CD8)		HLA-HIV 2-parameter score (based only in pVL and CD4)		HLA associations using Mann-Whitney U test (•, p<0.05 & q<0.2)					HLA associations using Generalized Linear Model (•, p<0.05 & q<0.2)					Plasma Viral Load							Absolut CD4							Z-score							Percentage CD4							CD4/CD8 ratio						
																	MW test		GLM					MW test		GLM					MW test		GLM					MW test		GLM					MW test		GLM				
			MW test	GLM	MW test	GLM	pVL	CD4	Z-score	%CD4	CD4/CD8	pVL	CD4	Z-score	%CD4	CD4/CD8	p-value	q-value	p-value	q-value	Coefficient	95% CI		p-value	q-value	p-value	q-value	Coefficient	95% CI		p-value	q-value	p-value	q-value	Coefficient	95% CI		p-value	q-value	p-value	q-value	Coefficient	95% CI		p-value	q-value	p-value	q-value	Coefficient	95% CI	
HLA-HIV PROTECTIVE associations	A*02:04	6	0	1	0	0										•	7.1E-01	8.8E-01	6.7E-01	8.8E-01	-0.185	-1.025	0.656	3.0E-01	6.8E-01	4.3E-01	7.9E-01	89.630	-133.809	313.069	3.9E-01	7.4E-01	4.9E-01	8.1E-01	0.252	-0.468	0.972	3.3E-01	6.9E-01	1.7E-01	5.9E-01	5.559	-2.309	13.427	7.5E-02	3.9E-01	1.5E-02	1.4E-01	0.336	0.066	0.607
	A*02:05	38	2	3	1	1		•	•				•	•	•		6.5E-02	3.6E-01	4.3E-02	2.9E-01	-0.348	-0.685	-0.011	1.0E-03	3.6E-02	1.0E-05	4.1E-04	216.004	126.906	305.101	2.9E-03	6.3E-02	1.0E-05	4.1E-04	0.561	0.273	0.849	2.3E-02	2.1E-01	1.6E-02	1.4E-01	3.871	0.715	7.027	2.4E-02	2.1E-01	6.5E-02	3.7E-01	0.103	-0.007	0.213
	A*03:01	162	1	1	1	1		•					•				4.1E-01	7.5E-01	3.1E-01	7.2E-01	-0.089	-0.262	0.084	1.4E-03	4.6E-02	5.0E-03	7.3E-02	66.284	20.219	112.349	2.3E-02	2.1E-01	3.7E-02	2.6E-01	0.158	0.010	0.307	1.4E-01	5.2E-01	1.4E-01	5.5E-01	1.235	-0.391	2.861	1.1E-01	4.6E-01	2.1E-01	6.5E-01	0.036	-0.020	0.092
	A*11:01	94	4	2	1	1		•	•	•	•		•	•			2.6E-02	2.2E-01	8.7E-02	4.3E-01	-0.196	-0.420	0.028	1.0E-02	1.3E-01	1.0E-03	2.7E-02	98.643	39.158	158.128	4.6E-03	8.0E-02	5.0E-03	7.3E-02	0.274	0.082	0.466	1.8E-02	1.8E-01	3.6E-02	2.5E-01	2.242	0.142	4.342	1.5E-02	1.6E-01	1.3E-01	5.4E-01	0.056	-0.016	0.128
	A*25:01	20	3	4	1	1		•		•	•		•	•	•	•	2.7E-01	6.7E-01	3.4E-01	7.3E-01	-0.225	-0.687	0.237	3.2E-03	6.4E-02	2.0E-03	4.2E-02	192.349	69.753	314.946	2.9E-02	2.3E-01	2.4E-02	1.9E-01	0.457	0.061	0.853	2.2E-03	5.9E-02	2.0E-03	4.2E-02	6.726	2.407	11.045	1.5E-03	4.6E-02	1.0E-05	4.1E-04	0.268	0.119	0.416
	A*30:02	116	2	2	1	1	•		•			•		•			1.0E-04	6.8E-03	1.0E-05	4.1E-04	-0.401	-0.598	-0.203	4.9E-02	3.0E-01	1.1E-01	4.9E-01	43.240	-9.503	95.984	1.5E-03	4.6E-02	1.0E-03	2.7E-02	0.278	0.108	0.447	4.2E-01	7.6E-01	3.4E-01	7.3E-01	0.908	-0.951	2.767	4.7E-01	7.8E-01	2.7E-01	7.0E-01	0.036	-0.029	0.100
	A*30:04	11	0	3	0	1							•		•	•	5.5E-01	8.2E-01	4.0E-01	7.7E-01	-0.269	-0.891	0.352	7.2E-02	3.8E-01	5.0E-03	7.3E-02	234.311	69.405	399.216	1.3E-01	5.0E-01	4.1E-02	2.8E-01	0.554	0.022	1.087	5.4E-02	3.2E-01	1.4E-02	1.3E-01	7.317	1.505	13.130	4.2E-02	2.8E-01	1.2E-02	1.2E-01	0.255	0.055	0.455
	A*80:01	5	0	2	0	0									•	•	3.0E-01	6.8E-01	1.8E-01	6.1E-01	-0.626	-1.545	0.294	1.9E-01	5.8E-01	5.1E-02	3.1E-01	243.814	-0.611	488.238	2.3E-01	6.3E-01	6.2E-02	3.6E-01	0.751	-0.037	1.539	1.1E-01	4.5E-01	1.4E-02	1.3E-01	10.773	2.168	19.377	1.4E-01	5.2E-01	1.0E-05	4.1E-04	0.554	0.259	0.849
	B*14:02	88	0	1	0	1							•				4.4E-01	7.7E-01	3.1E-01	7.2E-01	-0.119	-0.350	0.112	4.9E-02	3.1E-01	1.2E-02	1.2E-01	78.760	17.520	140.000	1.4E-01	5.1E-01	4.8E-02	3.0E-01	0.199	0.002	0.397	6.4E-01	8.7E-01	4.9E-01	8.1E-01	0.761	-1.400	2.923	8.8E-01	9.4E-01	7.1E-01	8.9E-01	0.014	-0.060	0.088
	B*15:10	38	0	1	0	1						•					5.9E-02	3.4E-01	1.3E-02	1.3E-01	-0.435	-0.776	-0.093	2.1E-01	6.1E-01	2.4E-01	6.8E-01	54.520	-36.419	145.459	7.3E-02	3.8E-01	3.5E-02	2.5E-01	0.315	0.022	0.608	3.0E-01	6.8E-01	3.8E-01	7.6E-01	1.444	-1.760	4.648	3.6E-01	7.2E-01	4.7E-01	7.9E-01	0.042	-0.070	0.153
	B*40:06	6	0	1	0	1							•				5.6E-01	8.3E-01	7.8E-01	9.1E-01	-0.129	-1.050	0.793	3.7E-02	2.7E-01	8.0E-03	9.8E-02	332.219	87.946	576.492	6.1E-02	3.4E-01	1.0E-01	4.7E-01	0.659	-0.130	1.448	1.3E-01	4.9E-01	1.6E-01	5.9E-01	6.137	-2.482	14.756	2.2E-01	6.2E-01	5.3E-01	8.2E-01	0.096	-0.200	0.393
	B*42:01	27	2	2	0	0				•	•				•	•	3.0E-01	6.8E-01	3.3E-01	7.3E-01	-0.201	-0.608	0.206	1.1E-01	4.6E-01	1.1E-01	5.1E-01	88.871	-21.273	199.014	7.1E-02	3.8E-01	1.3E-01	5.4E-01	0.277	-0.078	0.633	3.2E-03	6.4E-02	1.0E-03	2.7E-02	6.748	2.880	10.616	6.0E-04	2.5E-02	1.0E-05	4.1E-04	0.290	0.154	0.425
	B*50:01	18	0	2	0	1							•	•			1.0E-01	4.5E-01	1.2E-01	5.3E-01	-0.382	-0.869	0.105	4.5E-02	2.9E-01	4.0E-03	6.4E-02	189.916	60.664	319.169	5.4E-02	3.2E-01	1.3E-02	1.3E-01	0.531	0.114	0.949	3.7E-01	7.2E-01	2.2E-01	6.6E-01	2.828	-1.735	7.391	3.0E-01	6.8E-01	4.2E-02	2.8E-01	0.163	0.006	0.319
	B*51:07	6	1	3	0	1					•		•	•	•		2.6E-02	2.2E-01	7.9E-02	4.2E-01	-0.753	-1.593	0.088	5.7E-02	3.3E-01	1.6E-02	1.4E-01	273.406	50.243	496.568	2.4E-02	2.1E-01	1.8E-02	1.6E-01	0.868	0.148	1.588	3.0E-02	2.3E-01	2.2E-02	1.8E-01	9.220	1.358	17.082	1.5E-02	1.6E-01	4.6E-02	2.9E-01	0.276	0.005	0.546
	B*57:01	30	4	4	1	2		•	•	•	•	•	•	•	•		3.3E-02	2.5E-01	2.1E-02	1.8E-01	-0.478	-0.884	-0.071	1.8E-02	1.8E-01	2.0E-02	1.7E-01	128.219	20.283	236.156	1.3E-02	1.5E-01	8.0E-03	9.8E-02	0.469	0.121	0.817	3.6E-03	7.0E-02	4.0E-03	6.4E-02	5.574	1.776	9.372	7.7E-03	1.1E-01	2.9E-02	2.2E-01	0.145	0.015	0.276
	B*57:02	5	5	5	2	2	•	•	•	•	•	•	•	•	•	•	2.0E-02	1.9E-01	2.2E-02	1.8E-01	-1.078	-1.998	-0.159	1.4E-02	1.6E-01	8.0E-03	9.8E-02	331.617	87.342	575.893	8.2E-03	1.1E-01	5.0E-03	7.3E-02	1.136	0.348	1.924	4.2E-03	7.5E-02	1.0E-05	4.1E-04	17.775	9.198	26.353	1.1E-02	1.3E-01	1.0E-05	4.1E-04	1.093	0.766	1.420
	B*57:03	21	3	3	2	2	•	•	•			•	•	•			2.0E-04	1.1E-02	1.0E-05	4.1E-04	-0.973	-1.445	-0.500	1.9E-02	1.9E-01	5.0E-03	7.3E-02	181.444	55.585	307.304	2.0E-04	1.1E-02	1.0E-05	4.1E-04	0.813	0.408	1.219	4.2E-02	2.8E-01	5.7E-02	3.4E-01	4.308	-0.132	8.747	3.2E-02	2.5E-01	5.7E-02	3.4E-01	0.148	-0.005	0.301
	C*08:02	114	0	1	0	1							•				3.4E-01	7.1E-01	2.7E-01	7.0E-01	-0.114	-0.318	0.090	3.4E-02	2.6E-01	6.0E-03	8.5E-02	76.019	21.717	130.322	1.2E-01	4.7E-01	3.2E-02	2.4E-01	0.192	0.017	0.367	6.7E-01	8.8E-01	4.9E-01	8.1E-01	0.684	-1.241	2.608	8.5E-01	9.4E-01	9.1E-01	9.2E-01	0.004	-0.063	0.070
	C*12:03	96	2	2	1	1		•	•				•	•			7.9E-02	3.9E-01	5.6E-02	3.4E-01	-0.214	-0.433	0.005	6.5E-03	9.8E-02	1.0E-03	2.7E-02	96.669	38.269	155.069	7.5E-03	1.1E-01	3.0E-03	5.7E-02	0.283	0.095	0.472	6.4E-02	3.6E-01	3.5E-02	2.5E-01	2.223	0.154	4.292	1.6E-01	5.5E-01	6.8E-02	3.8E-01	0.066	-0.005	0.137
	C*17:01	60	3	4	1	1		•		•	•		•	•	•	•	1.8E-01	5.7E-01	1.3E-01	5.4E-01	-0.211	-0.484	0.062	9.1E-03	1.2E-01	7.0E-03	9.3E-02	100.928	27.793	174.062	2.5E-02	2.1E-01	1.5E-02	1.4E-01	0.294	0.058	0.530	1.4E-02	1.6E-01	8.0E-03	9.8E-02	3.520	0.934	6.106	6.4E-03	9.7E-02	4.0E-03	6.4E-02	0.131	0.042	0.221
	C*18:01	29	5	5	2	2	•	•	•	•	•	•	•	•	•	•	1.0E-04	6.8E-03	1.0E-05	4.1E-04	-0.825	-1.208	-0.441	5.0E-04	2.1E-02	1.0E-03	2.7E-02	181.381	79.126	283.636	1.0E-05	1.3E-03	1.0E-05	4.1E-04	0.739	0.410	1.068	4.9E-03	8.2E-02	2.0E-03	4.2E-02	5.789	2.171	9.407	2.6E-03	6.0E-02	1.0E-05	4.1E-04	0.261	0.135	0.387
HLA-HIV PROTECTIVE associations	A*02:01	538	-1	0	-1	0		•									4.9E-01	8.0E-01	6.0E-01	8.5E-01	0.030	-0.082	0.143	2.2E-02	1.9E-01	3.5E-02	2.5E-01	-32.362	-62.360	-2.363	1.4E-01	5.2E-01	1.6E-01	5.9E-01	-0.069	-0.166	0.028	3.8E-01	7.3E-01	4.6E-01	7.9E-01	-0.399	-1.457	0.659	2.1E-01	6.2E-01	3.1E-01	7.2E-01	-0.019	-0.055	0.018
	A*02:06	130	-1	-2	-1	-1		•					•			•	7.8E-01	9.1E-01	9.8E-01	9.3E-01	0.002	-0.189	0.193	1.3E-02	1.5E-01	5.0E-03	7.3E-02	-73.843	-124.911	-22.774	2.5E-01	6.5E-01	1.4E-01	5.5E-01	-0.125	-0.290	0.040	3.9E-02	2.8E-01	2.9E-02	2.2E-01	-2.006	-3.807	-0.205	2.3E-02	2.1E-01	1.3E-02	1.3E-01	-0.078	-0.140	-0.016
	A*24:02	466	-2	-2	-1	-1	•		•			•		•			1.1E-02	1.3E-01	1.4E-02	1.3E-01	0.147	0.030	0.264	9.3E-02	4.3E-01	3.3E-02	2.4E-01	-34.012	-65.213	-2.812	1.3E-02	1.5E-01	7.0E-03	9.3E-02	-0.137	-0.238	-0.037	7.8E-01	9.1E-01	4.1E-01	7.8E-01	-0.467	-1.568	0.633	6.4E-01	8.7E-01	6.4E-01	8.7E-01	-0.009	-0.047	0.029
	A*36:01	16	-1	0	-1	0	•										2.1E-02	2.0E-01	2.7E-02	2.1E-01	0.582	0.067	1.097	3.8E-01	7.3E-01	4.4E-01	7.9E-01	-54.467	-191.752	82.817	7.8E-02	3.9E-01	8.2E-02	4.3E-01	-0.392	-0.835	0.050	1.2E-01	4.9E-01	1.4E-01	5.5E-01	-3.690	-8.523	1.144	2.0E-01	6.1E-01	2.2E-01	6.6E-01	-0.105	-0.271	0.061
	A*68:01	148	-5	-5	-2	-2	•	•	•	•	•	•	•	•	•	•	3.9E-03	7.3E-02	8.0E-03	9.8E-02	0.245	0.065	0.424	3.0E-04	1.4E-02	1.0E-03	2.7E-02	-81.704	-129.388	-34.021	3.0E-04	1.4E-02	1.0E-03	2.7E-02	-0.272	-0.425	-0.118	1.0E-03	3.6E-02	3.0E-03	5.7E-02	-2.522	-4.204	-0.841	7.0E-04	2.8E-02	2.5E-02	2.0E-01	-0.067	-0.125	-0.008
	A*68:03	195	-5	-5	-2	-2	•	•	•	•	•	•	•	•	•	•	5.1E-03	8.4E-02	1.1E-02	1.2E-01	0.211	0.049	0.372	1.0E-04	6.8E-03	3.0E-03	5.7E-02	-64.070	-107.022	-21.118	3.0E-04	1.4E-02	2.0E-03	4.2E-02	-0.223	-0.361	-0.085	1.0E-04	6.8E-03	1.0E-05	4.1E-04	-2.820	-4.331	-1.310	2.0E-04	1.1E-02	1.0E-03	2.7E-02	-0.087	-0.139	-0.035
	B*35:02	20	-2	-2	-1	-1	•		•			•		•			1.2E-02	1.5E-01	7.0E-03	9.3E-02	0.668	0.182	1.154	8.8E-02	4.1E-01	7.1E-02	3.9E-01	-119.438	-248.905	10.029	9.7E-03	1.2E-01	1.0E-02	1.1E-01	-0.552	-0.970	-0.135	1.9E-01	5.8E-01	1.5E-01	5.7E-01	-3.357	-7.919	1.205	2.5E-01	6.5E-01	2.1E-01	6.5E-01	-0.100	-0.257	0.057
	B*35:12	114	-3	-3	-2	-2	•	•	•			•	•	•			1.0E-05	1.3E-03	1.0E-05	4.1E-04	0.408	0.202	0.615	8.7E-03	1.2E-01	1.6E-02	1.4E-01	-67.652	-122.845	-12.458	1.0E-05	1.3E-03	1.0E-05	4.1E-04	-0.336	-0.514	-0.158	2.5E-01	6.5E-01	4.5E-01	7.9E-01	-0.755	-2.702	1.192	2.7E-01	6.7E-01	7.0E-01	8.8E-01	-0.013	-0.080	0.054
	B*39:05	114	-4	-2	-1	-1		•	•	•	•		•	•			1.2E-01	4.9E-01	1.3E-01	5.4E-01	0.163	-0.047	0.374	1.0E-04	6.8E-03	1.0E-03	2.7E-02	-98.018	-153.845	-42.190	1.1E-03	3.9E-02	5.0E-03	7.3E-02	-0.260	-0.440	-0.080	1.1E-02	1.3E-01	3.2E-02	2.4E-01	-2.159	-4.130	-0.188	4.2E-03	7.5E-02	2.9E-01	7.1E-01	-0.036	-0.104	0.032
	C*03:03	75	-1	-1	-1	-1		•					•				5.0E-01	8.0E-01	5.1E-01	8.1E-01	0.083	-0.164	0.330	1.3E-02	1.5E-01	2.3E-02	1.9E-01	-76.201	-141.774	-10.628	7.0E-02	3.8E-01	9.5E-02	4.6E-01	-0.180	-0.392	0.031	7.3E-02	3.8E-01	1.1E-01	5.0E-01	-1.886	-4.206	0.435	1.5E-01	5.3E-01	1.6E-01	5.8E-01	-0.057	-0.137	0.023
	C*04:01	569	-2	-2	-1	-1	•		•			•		•			1.0E-04	6.8E-03	1.0E-05	4.1E-04	0.222	0.111	0.334	3.5E-01	7.1E-01	1.6E-01	5.8E-01	-21.461	-51.318	8.396	6.2E-03	9.6E-02	2.0E-03	4.2E-02	-0.150	-0.246	-0.054	8.1E-01	9.2E-01	8.1E-01	9.1E-01	-0.128	-1.184	0.929	9.9E-01	9.5E-01	7.3E-01	8.9E-01	-0.006	-0.043	0.030
	C*07:02	505	-3	-4	-1	-1		•	•		•		•	•	•	•	3.9E-02	2.8E-01	4.4E-02	2.9E-01	0.120	0.003	0.236	2.3E-03	5.9E-02	2.0E-03	4.2E-02	-50.231	-81.256	-19.206	2.3E-03	5.9E-02	3.0E-03	5.7E-02	-0.154	-0.254	-0.054	2.9E-02	2.3E-01	2.3E-02	1.9E-01	-1.273	-2.371	-0.174	4.5E-03	7.9E-02	2.5E-02	2.0E-01	-0.043	-0.081	-0.006

Supplementary Table S9. Summary of multivariable analyses in the MEX/CAM (top table) and individual cohorts (bottom tables). Independent models were constructed relating each HLA allele to each HIV clinical parameter, while adjusting for gender, age, geographical origin (country/region coded as n-1 binary variables) and the effect of the most significant HLA associations for that parameter (defined as the HLA alleles with p<0.001 in the corresponding Mann-Whitney univariable analysis). Accordingly, the MEX/CAM models were adjusted for the following alleles, pVL: B*57:03, C*18:01, C*04:01, B*57:01, A*30:02, B*35:12, B*27:05, C*07:02 & A*68:01; CD4: B*27:05, A*03:01, C*07:02, A*68:03, B*39:05, C*08:02, B*35:12, A*02:05, B*14:02, C*18:01; Z-score: B*57:03, C*18:01, B*27:05, C*07:02, B*39:05, B*35:12, B*57:01, B*14:02, C*08:02, C*04:01, A*68:03, A*03:01 & A*68:01; %CD4: B*27.05, A*68:03, B*39:05, B*57:01 & C*07:02; CD4/CD8 ratio: B*27:05, B*57:01, A*68:03, C*07:02, B*39:05, B*42:01 & C*18:01. MEX models were adjusted for the following, pVL: B*27:05, C*08:02, B*14:02 & B5701; CD4: B*27:05 & C*08:02; Z-score: B*27:05, B*14:02 & C*08:02; %CD4: B*27:05, B*39:05, C*08:02, B*14:02 & C*07:02; CD4/CD8 ratio: B*27:05, B*39:05 & A*68:03. CAM models were adjusted for next alleles, pVL: B*35:12, A*30:02, C*18:01, C*04:01 & B*57:03; CD4: B*39:05, A*68:03, A*68:01 & C*18:01; Z-score: C*18:01, B*35:12, B*57:03, A*68:01 & A*68:03; %CD4: A*68:03; CD4/CD8 ratio: A*68:03, B*42:01 & A*68:01. Associations were evaluated using linear regressions (Generalized Linear Model) and multiple comparisons were addresed with q-values. Significant (p<0.05, q<0.2) associations are shown in bold. Coefficients and 95% confidence intervals are shown.

Pooled MEX/CAM (n=3213) multivariable analysis

HIV outcome	Allele	N	HLA-HIV 5-parameter score (pVL, CD4, Z-score, %CD4, CD4/CD8)	HLA-HIV 2-parameter score (based only in pVL and CD4)	HLA associations using Generalized Linear Model (●, p<0.05 & q<0.2)					Plasma Viral Load					Absolut CD4					Z-score					Percentage CD4					CD4/CD8 ratio				
					pVL	CD4	Z-score	%CD4	CD4/CD8	p-value	q-value	Coef.	95% Conf. Interval		p-value	q-value	Coef.	95% Conf. Interval		p-value	q-value	Coef.	95% Conf. Interval		p-value	q-value	Coef.	95% Conf. Interval		p-value	q-value	Coef.	95% Conf. Interval	
HLA-HIV PROTECTIVE associations	***A*02:05***	71	3	1		●	●	●		1.7E-01	2.5E-01	-0.158	-0.384	0.068	**1.0E-05**	**1.6E-05**	137.171	73.919	200.423	**2.0E-03**	**1.9E-03**	0.304	0.108	0.501	**1.7E-02**	**1.9E-02**	2.769	0.505	5.033	2.7E-01	**7.5E-02**	0.045	-0.034	0.124
	A*02:06	441	1	1	●					**2.1E-02**	**6.5E-02**	-0.119	-0.220	-0.018	1.0E-01	**2.3E-02**	-24.062	-52.778	4.654	6.7E-01	**7.9E-02**	0.020	-0.070	0.109	1.9E-01	**8.1E-02**	-0.695	-1.723	0.333	6.3E-02	**4.0E-02**	-0.033	-0.068	0.002
	A*03:01	299	3	1		●	●	●		2.7E-01	3.3E-01	-0.066	-0.183	0.051	**1.0E-05**	**1.6E-05**	62.509	29.394	95.624	**1.8E-02**	**9.5E-03**	0.124	0.021	0.227	**7.0E-03**	**1.1E-02**	1.644	0.456	2.831	8.4E-02	**4.5E-02**	0.036	-0.005	0.076
	A*11:01	202	1	1		●				4.0E-01	4.4E-01	-0.060	-0.200	0.079	**1.5E-02**	**7.8E-03**	48.378	9.326	87.429	1.4E-01	**2.5E-02**	0.092	-0.029	0.214	1.6E-01	**7.2E-02**	1.014	-0.387	2.416	2.5E-01	**7.3E-02**	0.028	-0.020	0.076
	A*25:01	44	3	1		●		●	●	5.6E-01	5.4E-01	-0.085	-0.368	0.199	**5.0E-03**	**3.9E-03**	113.640	34.278	193.002	5.0E-02	**1.7E-02**	0.247	0.000	0.494	**1.2E-02**	**1.5E-02**	3.623	0.781	6.466	**9.0E-03**	**1.0E-02**	0.130	0.033	0.226
	A*26:01	143	1	1		●				3.9E-01	4.4E-01	-0.071	-0.234	0.092	**2.6E-02**	**9.9E-03**	51.819	6.202	97.436	1.0E-01	**2.4E-02**	0.118	-0.024	0.260	3.0E-01	**1.2E-01**	0.859	-0.775	2.494	7.9E-01	**1.6E-01**	0.008	-0.048	0.063
	A*30:02	171	1	1	●					**1.4E-02**	**5.5E-02**	-0.189	-0.340	-0.039	4.8E-01	**6.9E-02**	15.084	-27.131	57.299	1.6E-01	**2.6E-02**	0.095	-0.036	0.227	5.4E-01	**1.9E-01**	0.477	-1.035	1.989	6.2E-01	**1.3E-01**	0.013	-0.039	0.065
	B*07:02	301	2	1		●		●		2.6E-01	3.2E-01	-0.075	-0.203	0.054	**7.0E-03**	**4.9E-03**	52.651	14.196	91.105	8.1E-02	**2.0E-02**	0.107	-0.013	0.227	**4.0E-02**	**3.2E-02**	1.420	0.067	2.774	4.0E-01	**1.0E-01**	0.020	-0.026	0.066
	B*14:02	189	3	1	●			●	●	**7.0E-03**	**4.0E-02**	-0.201	-0.346	-0.056	1.6E-01	**2.9E-02**	45.733	-17.874	109.340	1.3E-01	**2.5E-02**	0.153	-0.044	0.351	**1.0E-05**	**4.2E-05**	2.624	1.167	4.082	**1.4E-02**	**1.4E-02**	0.063	0.013	0.113
	B*15:39	8	4	2	●	●	●		●	**1.0E-03**	**1.0E-02**	-1.164	-1.824	-0.504	**2.7E-02**	**9.9E-03**	209.068	24.219	393.917	**1.0E-03**	**1.5E-03**	0.958	0.384	1.533	6.4E-02	**4.3E-02**	6.264	-0.363	12.890	**4.4E-02**	**3.3E-02**	0.232	0.006	0.458
	B*27:05	65	5	2	●	●	●	●	●	**1.0E-05**	**2.0E-04**	-0.529	-0.768	-0.289	**1.0E-05**	**1.6E-05**	203.067	136.197	269.937	**1.0E-05**	**2.9E-05**	0.630	0.422	0.839	**1.0E-05**	**4.2E-05**	7.438	5.045	9.832	**1.0E-05**	**2.5E-05**	0.242	0.160	0.323
	B*39:02	98	3	1	●			●	●	**2.8E-02**	**7.5E-02**	-0.222	-0.419	-0.025	2.3E-01	**3.9E-02**	34.246	-21.586	90.078	5.4E-02	**1.7E-02**	0.171	-0.003	0.344	**3.0E-02**	**2.5E-02**	2.228	0.221	4.236	**2.0E-03**	**2.9E-03**	0.111	0.042	0.179
	B*40:05	18	1	1		●				9.2E-01	7.8E-01	0.023	-0.432	0.478	**2.1E-02**	**9.2E-03**	149.796	22.592	277.000	2.1E-01	**3.2E-02**	0.252	-0.144	0.649	1.2E-01	**5.9E-02**	3.659	-0.901	8.218	4.0E-01	**1.0E-01**	0.067	-0.089	0.223
	B*40:06	8	2	1		●	●			2.8E-01	3.4E-01	-0.388	-1.098	0.321	**1.7E-02**	**8.2E-03**	242.866	44.258	441.473	**3.8E-02**	**1.6E-02**	0.653	0.036	1.271	2.7E-01	**1.1E-01**	3.988	-3.130	11.105	5.8E-01	**1.3E-01**	0.069	-0.174	0.312
	B*42:01	42	2	0				●	●	1.7E-01	2.5E-01	-0.205	-0.501	0.090	5.6E-02	**1.6E-02**	81.431	-2.180	165.042	6.5E-02	**1.8E-02**	0.245	-0.015	0.505	**5.0E-03**	**9.3E-03**	4.293	1.300	7.285	**1.0E-03**	**1.7E-03**	0.169	0.066	0.273
	B*44:03	221	0	0						9.7E-01	7.8E-01	0.002	-0.133	0.138	5.6E-01	**7.8E-02**	11.195	-26.880	49.269	7.7E-01	**8.9E-02**	0.018	-0.101	0.136	3.3E-01	**1.3E-01**	0.676	-0.687	2.040	7.3E-02	**4.4E-02**	0.043	-0.004	0.089
	B*57:01	61	5	2	●	●	●	●	●	**1.0E-05**	**2.0E-04**	-0.588	-0.842	-0.333	**1.0E-05**	**1.6E-05**	133.403	62.381	204.425	**1.0E-05**	**2.9E-05**	0.531	0.310	0.753	**1.0E-05**	**4.2E-05**	5.259	2.709	7.808	**1.0E-05**	**2.5E-05**	0.234	0.148	0.321
	B*57:02	7	2	0				●	●	9.6E-01	7.8E-01	0.020	-0.737	0.776	6.3E-01	**8.5E-02**	51.714	-156.487	259.915	5.9E-01	**7.2E-02**	0.180	-0.477	0.838	**3.0E-03**	**8.4E-03**	10.840	3.755	17.925	**1.0E-05**	**2.5E-05**	0.595	0.325	0.866
	B*57:03	38	4	2	●	●	●	●		**1.0E-03**	**1.0E-02**	-0.604	-0.972	-0.235	**2.2E-02**	**9.2E-03**	119.893	16.950	222.836	**1.0E-03**	**1.5E-03**	0.535	0.215	0.855	**1.1E-02**	**1.5E-02**	4.279	1.001	7.558	1.8E-01	**6.4E-02**	0.087	-0.039	0.213
	B*58:01	85	0	0						4.2E-01	4.5E-01	-0.087	-0.296	0.122	5.7E-01	**7.8E-02**	16.968	-41.991	75.928	2.9E-01	**4.2E-02**	0.098	-0.084	0.280	5.1E-01	**1.9E-01**	0.704	-1.392	2.800	8.8E-01	**1.8E-01**	-0.005	-0.077	0.066
	C*02:02	162	0	0						5.3E-01	5.3E-01	-0.054	-0.221	0.114	6.8E-01	**9.7E-02**	9.769	-37.151	56.688	5.4E-01	**6.8E-02**	0.046	-0.100	0.192	4.5E-01	**1.7E-01**	0.653	-1.026	2.331	2.2E-01	**7.0E-02**	0.036	-0.022	0.093
	C*03:02	71	0	0						8.5E-01	7.5E-01	-0.022	-0.253	0.209	6.5E-01	**8.5E-02**	15.035	-49.495	79.565	7.8E-01	**1.1E-01**	0.029	-0.172	0.230	8.4E-01	2.9E-01	0.244	-2.065	2.554	6.2E-01	**1.3E-01**	-0.020	-0.099	0.059
	C*03:05	222	0	0						1.8E-01	2.5E-01	-0.094	-0.230	0.042	7.7E-01	**1.2E-01**	-5.748	-44.136	32.639	4.7E-01	**6.4E-02**	0.044	-0.076	0.164	9.6E-01	3.2E-01	-0.037	-1.407	1.333	7.7E-01	**1.6E-01**	0.007	-0.040	0.054
	C*08:01	160	1	0					●	9.8E-02	**1.9E-01**	-0.133	-0.291	0.025	4.7E-01	**6.8E-02**	16.228	-27.847	60.302	1.6E-01	**2.6E-02**	0.099	-0.039	0.237	8.6E-02	**4.8E-02**	1.384	-0.194	2.962	**2.8E-02**	**2.4E-02**	0.060	0.006	0.115
	C*08:02	246	4	2	●	●		●	●	**9.0E-03**	**4.0E-02**	-0.172	-0.301	-0.043	**4.9E-02**	**1.6E-02**	56.555	0.131	112.980	1.1E-01	**2.4E-02**	0.143	-0.033	0.319	**1.0E-05**	**4.2E-05**	2.421	1.129	3.713	**2.3E-02**	**2.1E-02**	0.051	0.007	0.095
	C*12:02	67	0	0						7.1E-02	**1.5E-01**	-0.216	-0.451	0.019	3.2E-01	**4.9E-02**	33.158	-32.509	98.825	1.1E-01	**2.4E-02**	0.166	-0.039	0.370	1.4E-01	**6.9E-02**	1.753	-0.598	4.104	1.9E-01	**6.4E-02**	0.054	-0.027	0.135
	C*12:03	189	3	1		●	●	●		1.8E-01	2.5E-01	-0.099	-0.244	0.045	**4.0E-03**	**3.6E-03**	59.418	18.972	99.864	**1.1E-02**	**7.1E-03**	0.163	0.037	0.290	**2.3E-02**	**2.4E-02**	1.675	0.232	3.118	8.2E-02	**4.5E-02**	0.044	-0.005	0.093
	C*14:02	55	4	2	●	●	●	●		**3.5E-02**	**8.8E-02**	-0.277	-0.533	-0.020	**2.0E-03**	**2.1E-03**	111.151	39.302	183.000	**4.0E-03**	**3.3E-03**	0.328	0.104	0.551	**3.0E-02**	**2.5E-02**	2.854	0.281	5.426	1.7E-01	**6.4E-02**	0.061	-0.027	0.149
	C*14:03	6	2	1	●		●			**1.7E-02**	**5.7E-02**	-0.930	-1.693	-0.168	1.4E-01	**2.8E-02**	162.804	-50.536	376.143	**4.3E-02**	**1.7E-02**	0.684	0.021	1.348	1.1E-01	**5.7E-02**	6.328	-1.325	13.982	9.6E-02	**4.8E-02**	0.221	-0.039	0.482
	C*17:01	106	0	0						9.3E-01	7.8E-01	0.009	-0.180	0.198	1.5E-01	**2.9E-02**	39.029	-13.886	91.943	5.3E-01	**6.8E-02**	0.053	-0.112	0.219	1.8E-01	**7.9E-02**	1.311	-0.584	3.205	4.5E-01	**1.1E-01**	-0.030	-0.107	0.048
	C*18:01	37	4	1		●	●	●	●	8.4E-02	**1.7E-01**	-0.312	-0.666	0.041	**1.0E-03**	**1.3E-03**	149.939	61.968	237.911	**2.8E-02**	**1.3E-02**	0.346	0.038	0.653	**1.0E-03**	**3.3E-03**	5.363	2.216	8.509	**1.0E-05**	**2.5E-05**	0.299	0.190	0.408
…tions	***A*01:02***	8	-2	-1	●		●			**5.0E-03**	**3.4E-02**	0.950	0.289	1.611	1.4E-01	**2.8E-02**	-139.058	-324.166	46.050	**1.5E-02**	**8.7E-03**	-0.715	-1.290	-0.140	2.7E-01	**1.1E-01**	-3.754	-10.385	2.878	1.6E-01	**6.3E-02**	-0.175	-0.416	0.066
	A*02:01	1278	0	0						5.3E-01	5.3E-01	0.023	-0.049	0.094	2.5E-01	**4.2E-02**	-11.795	-32.015	8.425	1.9E-01	**3.1E-02**	-0.042	-0.106	0.021	6.9E-02	**4.3E-02**	-0.657	-1.364	0.050	1.4E-01	**6.1E-02**	-0.018	-0.042	0.006
	A*24:02	966	0	0						5.0E-02	**1.2E-01**	0.075	0.000	0.150	4.4E-01	**6.6E-02**	-8.307	-29.492	12.878	6.4E-02	**1.8E-02**	-0.063	-0.129	0.004	7.7E-01	2.7E-01	-0.111	-0.864	0.641	4.0E-01	**1.0E-01**	0.011	-0.015	0.037
	A*68:01	382	-5	-2	●	●	●	●	●	**9.0E-03**	**4.0E-02**	0.140	0.035	0.244	**8.0E-03**	**5.0E-03**	-39.877	-69.388	-10.367	**2.0E-03**	**1.9E-03**	-0.149	-0.240	-0.057	**4.0E-03**	**9.3E-03**	-1.567	-2.622	-0.512	**4.5E-02**	**3.3E-02**	-0.037	-0.073	-0.001
	A*68:03	370	-2	0				●	●	1.6E-01	2.5E-01	0.079	-0.031	0.189	1.5E-01	**2.9E-02**	-23.637	-56.167	8.892	1.4E-01	**2.5E-02**	-0.076	-0.177	0.026	**5.0E-03**	**9.3E-03**	-1.655	-2.822	-0.488	**3.0E-03**	**3.8E-03**	-0.060	-0.100	-0.020
	A*68:05	60	-1	0				●		6.2E-01	5.8E-01	0.063	-0.187	0.314	6.1E-01	**8.5E-02**	-18.457	-88.616	51.703	5.3E-01	**6.8E-02**	-0.070	-0.288	0.148	**1.5E-02**	**1.8E-02**	-3.129	-5.659	-0.599	1.5E-01	**6.3E-02**	-0.063	-0.149	0.023
	B*13:02	65	0	0						1.8E-01	2.5E-01	0.164	-0.074	0.403	3.1E-01	**4.8E-02**	-34.629	-101.203	31.946	1.3E-01	**2.5E-02**	-0.159	-0.366	0.049	1.4E-01	**6.7E-02**	-1.811	-4.194	0.572	2.5E-01	**7.3E-02**	-0.048	-0.129	0.034

HIV outcome	Allele	N	HLA-HIV 5-parameter score (pVL, CD4, Z-score, %CD4, CD4/CD8)	HLA-HIV 2-parameter score (based only in pVL and CD4)	pVL	CD4	Z-score	%CD4	CD4/CD8	PVL p-value	PVL q-value	PVL Coef.	PVL 95% Conf. Interval		CD4 p-value	CD4 q-value	CD4 Coef.	CD4 95% Conf. Interval		Z p-value	Z q-value	Z Coef.	Z 95% Conf. Interval		%CD4 p-value	%CD4 q-value	%CD4 Coef.	%CD4 95% Conf. Interval		CD4/CD8 p-value	CD4/CD8 q-value	CD4/CD8 Coef.	CD4/CD8 95% Conf. Interval	
HLA-HIV RISK associat	B*15:30	20	-1	0				●		9.9E-01	7.8E-01	0.003	-0.427	0.433	7.2E-02	1.9E-02	-110.308	-230.662	10.047	3.1E-01	4.4E-02	-0.192	-0.567	0.182	6.0E-03	1.0E-02	-6.064	-10.373	-1.756	1.0E-01	4.8E-02	-0.126	-0.277	0.024
	B*35:01	645	-1	0				●		5.3E-01	5.3E-01	0.031	-0.064	0.126	5.7E-02	1.6E-02	-23.885	-48.491	0.720	3.2E-01	4.4E-02	-0.043	-0.126	0.041	3.0E-02	2.5E-02	-0.974	-1.855	-0.093	1.4E-01	6.1E-02	-0.023	-0.053	0.007
	B*35:02	51	-2	-1	●		●			1.5E-02	5.5E-02	0.340	0.067	0.613	9.8E-02	2.3E-02	-63.581	-138.873	11.711	2.9E-02	1.3E-02	-0.265	-0.503	-0.027	8.5E-02	4.8E-02	-2.368	-5.064	0.328	5.7E-02	3.9E-02	-0.089	-0.181	0.002
	B*35:12	273	-1	-1		●				1.8E-01	2.5E-01	0.092	-0.042	0.225	1.4E-02	7.8E-03	-43.890	-79.004	-8.777	7.9E-02	2.0E-02	-0.105	-0.223	0.012	6.2E-02	4.3E-02	-1.193	-2.447	0.062	5.6E-01	1.3E-01	-0.013	-0.056	0.030
	B*35:14	38	0	0						4.5E-01	4.7E-01	0.121	-0.191	0.434	7.3E-02	1.9E-02	-80.439	-168.232	7.353	1.1E-01	2.4E-02	-0.222	-0.498	0.053	3.7E-01	1.4E-01	-1.460	-4.650	1.729	2.3E-01	7.0E-02	-0.067	-0.176	0.042
	B*39:01	127	0	0						7.7E-01	7.1E-01	0.026	-0.152	0.205	1.1E-01	2.3E-02	-41.867	-92.441	8.708	2.7E-01	4.1E-02	-0.088	-0.246	0.069	1.2E-01	5.9E-02	-1.462	-3.281	0.356	2.9E-01	8.0E-02	-0.033	-0.095	0.029
	B*39:05	360	-2	-1		●		●		2.3E-01	3.0E-01	0.076	-0.047	0.200	3.1E-02	1.1E-02	-40.215	-76.836	-3.595	9.2E-02	2.2E-02	-0.098	-0.212	0.016	2.4E-02	2.4E-02	-1.507	-2.818	-0.196	3.4E-01	9.2E-02	-0.022	-0.066	0.023
	B*39:06	153	0	0						2.5E-01	3.2E-01	0.101	-0.070	0.273	2.0E-01	3.4E-02	-32.438	-81.488	16.611	1.2E-01	2.4E-02	-0.122	-0.274	0.031	9.8E-01	3.2E-01	-0.020	-1.778	1.738	9.4E-01	1.9E-01	-0.002	-0.062	0.057
	B*40:01	46	0	0						7.2E-02	1.5E-01	0.257	-0.023	0.538	1.8E-01	3.3E-02	-53.532	-132.182	25.117	6.3E-02	1.8E-02	-0.232	-0.477	0.013	6.2E-02	4.3E-02	-2.680	-5.497	0.136	1.9E-01	6.4E-02	-0.065	-0.161	0.031
	B*81:01	16	-1	0					●	8.4E-01	7.5E-01	-0.062	-0.654	0.529	1.0E-01	2.3E-02	-129.254	-284.657	26.150	5.5E-01	6.8E-02	-0.157	-0.671	0.357	7.8E-01	2.7E-01	0.680	-4.026	5.385	1.0E-03	1.7E-03	-0.317	-0.508	-0.127
	C*03:04	427	-1	0				●		1.4E-01	2.5E-01	0.078	-0.025	0.181	2.8E-01	4.4E-02	-15.866	-44.387	12.656	2.1E-01	3.2E-02	-0.058	-0.149	0.032	4.2E-02	3.2E-02	-1.054	-2.072	-0.037	1.7E-01	6.4E-02	-0.024	-0.059	0.010
	C*04:01	1237	-2	-1	●		●			3.0E-03	2.4E-02	0.116	0.039	0.193	8.7E-02	2.2E-02	-18.893	-40.558	2.772	9.0E-03	6.5E-03	-0.089	-0.157	-0.022	6.8E-02	4.3E-02	-0.669	-1.388	0.050	4.3E-01	1.0E-01	-0.010	-0.035	0.015
	C*07:02	1171	-1	-1	●					2.5E-02	7.2E-02	0.084	0.011	0.158	5.2E-02	1.6E-02	-22.708	-45.631	0.215	5.0E-02	1.7E-02	-0.072	-0.144	0.000	7.6E-02	4.5E-02	-0.737	-1.553	0.078	2.1E-01	6.7E-02	-0.018	-0.046	0.010

MEX (n=1679) multivariable analysis

HIV outcome	Allele	N	HLA-HIV 5-parameter score (pVL, CD4, Z-score, %CD4, CD4/CD8)	HLA-HIV 2-parameter score (based only in pVL and CD4)	HLA associations using Generalized Linear Model (●, p<0.05 & q<0.2)					Plasma Viral Load					Absolut CD4					Z-score					Percentage CD4					CD4/CD8 ratio				
					pVL	CD4	Z-score	%CD4	CD4/CD8	p-value	q-value	Coef.	95% Conf. Interval		p-value	q-value	Coef.	95% Conf. Interval		p-value	q-value	Coef.	95% Conf. Interval		p-value	q-value	Coef.	95% Conf. Interval		p-value	q-value	Coef.	95% Conf. Interval	
HLA-HIV PROTECTIVE associations	A*03:01	137	3	1		●	●	●		2.0E-01	3.8E-02	-0.105	-0.266	0.056	1.0E-03	1.0E-03	87.157	37.971	136.344	5.0E-03	4.2E-03	0.214	0.065	0.363	9.0E-03	3.4E-04	2.425	0.608	4.241	1.3E-01	6.6E-02	0.047	-0.014	0.108
	A*26:01	62	2	1		●	●			2.8E-01	4.9E-02	-0.130	-0.365	0.105	1.7E-02	9.6E-03	85.535	15.614	155.457	3.1E-02	1.9E-02	0.235	0.021	0.448	6.5E-01	6.1E-03	0.600	-1.990	3.189	9.6E-01	3.3E-01	-0.002	-0.089	0.084
	B*14:02	101	1	0					●	1.7E-01	3.7E-02	-0.202	-0.487	0.084	1.1E-01	3.8E-02	70.855	-14.816	156.526	7.5E-02	3.6E-02	0.236	-0.024	0.496	1.9E-01	2.1E-03	2.111	-1.037	5.259	2.0E-02	2.1E-02	0.084	0.013	0.154
	B*15:39	6	5	2	●	●	●	●	●	1.0E-05	1.1E-05	-1.402	-2.121	-0.684	4.0E-03	2.8E-03	318.107	102.483	533.731	1.0E-05	1.4E-05	1.267	0.615	1.920	5.0E-03	2.8E-04	11.334	3.490	19.178	6.0E-03	1.0E-02	0.372	0.108	0.636
	B*27:05	46	5	2	●	●	●	●	●	1.0E-05	1.1E-05	-0.678	-0.948	-0.409	1.0E-05	2.1E-05	281.774	201.062	362.485	1.0E-05	1.4E-05	0.845	0.600	1.090	1.0E-05	2.3E-06	9.249	6.298	12.199	1.0E-05	2.9E-05	0.289	0.190	0.388
	B*39:02	68	3	1	●			●	●	1.2E-02	5.0E-03	-0.284	-0.505	-0.063	5.5E-01	1.4E-01	20.225	-46.158	86.609	8.4E-02	3.6E-02	0.177	-0.024	0.378	2.3E-02	5.8E-04	2.913	0.402	5.424	4.0E-03	8.6E-03	0.121	0.038	0.203
	B*40:05	18	1	1		●				8.9E-01	1.2E-01	-0.030	-0.460	0.399	1.9E-02	9.8E-03	153.089	24.732	281.446	1.5E-01	4.9E-02	0.288	-0.102	0.678	1.2E-01	1.8E-03	3.707	-0.972	8.387	3.3E-01	1.4E-01	0.078	-0.080	0.235
	B*57:01	31	5	2	●	●	●	●	●	1.0E-05	1.1E-05	-0.809	-1.139	-0.478	5.0E-03	3.1E-03	142.452	43.758	241.146	1.0E-05	1.4E-05	0.661	0.363	0.960	4.0E-03	2.8E-04	5.297	1.701	8.893	1.0E-05	2.9E-05	0.311	0.190	0.431
	B*57:03	17	5	2	●	●	●	●	●	1.0E-03	6.7E-04	-0.816	-1.288	-0.344	3.0E-03	2.7E-03	211.301	69.935	352.667	1.0E-05	1.4E-05	0.785	0.357	1.213	2.7E-02	6.1E-04	5.833	0.671	10.995	1.0E-05	2.9E-05	0.331	0.158	0.504
	C*02:02	74	0	0						1.9E-01	3.8E-02	-0.169	-0.420	0.082	3.7E-01	1.0E-01	34.934	-40.883	110.752	2.2E-01	6.5E-02	0.144	-0.086	0.374	7.7E-01	7.0E-03	0.410	-2.352	3.172	4.5E-01	1.9E-01	0.036	-0.057	0.129
	C*03:03	121	4	1	●		●	●	●	2.0E-03	1.1E-03	-0.267	-0.436	-0.097	1.1E-01	3.9E-02	41.231	-9.880	92.342	1.3E-02	9.8E-03	0.197	0.042	0.352	2.1E-02	5.8E-04	2.220	0.332	4.108	1.4E-02	1.7E-02	0.080	0.016	0.144
	C*08:01	143	1	0					●	5.3E-02	1.4E-02	-0.156	-0.314	0.002	3.6E-01	1.0E-01	21.940	-25.480	69.360	1.2E-01	4.8E-02	0.113	-0.030	0.257	1.7E-01	2.1E-03	1.224	-0.510	2.958	4.2E-02	3.3E-02	0.061	0.002	0.119
	C*08:02	132	2	1		●			●	3.1E-01	5.2E-02	-0.131	-0.384	0.122	1.0E-05	2.1E-05	108.668	59.084	158.252	1.4E-01	4.9E-02	0.172	-0.057	0.402	2.1E-01	2.3E-03	1.768	-1.014	4.551	2.6E-02	2.5E-02	0.070	0.008	0.132
	C*14:02	25	5	2	●	●	●	●	●	1.0E-03	6.7E-04	-0.596	-0.957	-0.234	1.0E-05	2.1E-05	217.806	109.801	325.811	1.0E-05	1.4E-05	0.701	0.374	1.029	1.0E-03	1.1E-04	6.614	2.669	10.558	1.4E-02	1.7E-02	0.167	0.034	0.300
HLA-HIV RISK associations	A*23:01	59	-1	-1	●					3.0E-02	1.0E-02	0.260	0.025	0.495	9.0E-01	2.2E-01	-4.490	-75.155	66.174	1.8E-01	5.4E-02	-0.147	-0.361	0.067	4.9E-01	4.8E-03	0.917	-1.659	3.492	8.0E-01	2.9E-01	0.011	-0.076	0.098
	A*24:03	6	-1	-1	●					3.8E-02	1.2E-02	0.758	0.040	1.476	2.1E-01	6.8E-02	138.916	-76.892	354.724	6.8E-01	2.4E-01	-0.137	-0.791	0.517	4.0E-01	4.2E-03	3.346	-4.508	11.199	6.9E-01	2.6E-01	0.055	-0.210	0.319
	A*68:03	175	-3	-1		●	●		●	3.9E-01	6.2E-02	0.064	-0.081	0.209	4.0E-03	2.8E-03	-64.045	-107.786	-20.303	2.6E-02	1.8E-02	-0.150	-0.283	-0.018	1.8E-01	2.1E-03	-1.222	-3.015	0.572	4.0E-02	3.3E-02	-0.063	-0.123	-0.003
	B*15:30	17	-1	0				●		6.6E-01	9.6E-02	-0.099	-0.542	0.344	1.0E-01	3.8E-02	-110.855	-243.351	21.642	4.4E-01	1.2E-01	-0.157	-0.559	0.245	1.3E-02	4.2E-04	-6.096	-10.912	-1.280	1.2E-01	6.6E-02	-0.129	-0.292	0.033
	B*35:12	159	-1	-1		●				7.5E-01	1.0E-01	0.025	-0.129	0.180	2.9E-02	1.4E-02	-52.026	-98.636	-5.415	1.5E-01	4.9E-02	-0.103	-0.245	0.038	9.6E-02	1.7E-03	-1.449	-3.153	0.255	6.6E-01	2.6E-01	-0.013	-0.070	0.045
	B*37:01	11	-1	-1	●					2.8E-02	1.0E-02	0.600	0.066	1.133	3.9E-01	1.0E-01	-70.261	-230.038	89.517	8.6E-02	3.6E-02	-0.425	-0.910	0.060	1.4E-01	1.9E-03	-4.352	-10.176	1.472	1.3E-01	6.6E-02	-0.151	-0.347	0.045
	B*39:05	246	-3	-1		●	●	●		9.3E-02	2.2E-02	0.111	-0.018	0.240	1.0E-03	1.0E-03	-66.059	-104.814	-27.304	2.0E-03	1.9E-03	-0.190	-0.307	-0.072	8.0E-03	3.4E-04	-2.192	-3.808	-0.576	6.2E-02	4.1E-02	-0.051	-0.104	0.003
	C*04:01	668	0	0						4.2E-01	6.4E-02	0.038	-0.055	0.130	8.4E-02	3.5E-02	-24.341	-51.949	3.267	7.5E-02	3.6E-02	-0.076	-0.160	0.008	1.3E-01	1.8E-03	-0.801	-1.825	0.222	2.7E-01	1.2E-01	-0.019	-0.053	0.015
	C*06:02	140	-1	-1	●					3.0E-03	1.4E-03	0.250	0.085	0.416	3.7E-01	1.0E-01	-22.165	-70.312	25.981	1.5E-01	4.9E-02	-0.108	-0.254	0.038	6.3E-02	1.2E-03	-1.668	-3.427	0.091	9.7E-02	6.0E-02	-0.050	-0.110	0.009
	C*07:02	666	-3	-2	●	●	●			4.7E-02	1.3E-02	0.094	0.001	0.187	1.0E-03	1.0E-03	-45.566	-73.440	-17.693	2.0E-03	1.9E-03	-0.135	-0.219	-0.050	1.2E-01	1.8E-03	-0.921	-2.085	0.244	2.7E-01	1.2E-01	-0.022	-0.062	0.017
	C*16:01	98	-1	0				●		2.0E-01	3.8E-02	0.124	-0.066	0.313	5.7E-02	2.5E-02	-54.903	-111.537	1.730	5.2E-02	2.9E-02	-0.171	-0.342	0.001	3.2E-02	6.6E-04	-2.285	-4.379	-0.191	5.3E-02	3.8E-02	-0.070	-0.140	0.001

CAM (n=1534) multivariable analysis

HIV outcome	Allele	N	HLA-HIV 5-parameter score (pVL, CD4, Z-score, %CD4, CD4/CD8)	HLA-HIV 2-parameter score (based only in pVL and CD4)	HLA associations using Generalized Linear Model (●, p<0.05 & q<0.2)					Plasma Viral Load					Absolut CD4					Z-score					Percentage CD4					CD4/CD8 ratio				
					pVL	CD4	Z-score	%CD4	CD4/CD8	p-value	q-value	Coef.	95% Conf. Interval		p-value	q-value	Coef.	95% Conf. Interval		p-value	q-value	Coef.	95% Conf. Interval		p-value	q-value	Coef.	95% Conf. Interval		p-value	q-value	Coef.	95% Conf. Interval	
:CTIVE associations	A*02:05	38	3	1		●	●	●		1.0E-01	4.9E-02	-0.273	-0.600	0.053	1.0E-05	3.2E-05	187.488	101.809	273.166	2.0E-03	6.4E-03	0.446	0.170	0.722	1.8E-02	2.2E-03	3.622	0.633	6.610	9.3E-02	1.4E-01	0.091	-0.015	0.197
	A*03:01	162	1	1		●				6.1E-01	1.5E-01	-0.044	-0.213	0.124	4.1E-02	2.4E-02	46.582	1.988	91.176	3.3E-01	9.5E-02	0.072	-0.072	0.216	1.5E-01	8.5E-03	1.124	-0.422	2.670	4.0E-01	3.2E-01	0.023	-0.031	0.078
	A*11:01	94	2	1		●	●			8.8E-02	4.8E-02	-0.189	-0.405	0.028	6.0E-03	6.4E-03	80.225	22.991	137.458	2.1E-02	1.2E-02	0.217	0.033	0.401	8.7E-02	6.6E-03	1.737	-0.253	3.727	2.5E-01	2.4E-01	0.041	-0.029	0.111
	A*25:01	20	4	1		●	●	●	●	3.3E-01	9.8E-02	-0.223	-0.669	0.223	1.0E-03	2.1E-03	197.811	80.585	315.037	2.0E-02	1.2E-02	0.450	0.072	0.827	1.0E-03	5.0E-04	7.157	3.081	11.232	1.0E-05	5.1E-05	0.262	0.119	0.405
	A*30:02	116	2	1	●		●			3.0E-03	1.8E-02	-0.292	-0.486	-0.097	4.6E-01	2.3E-01	19.150	-32.021	70.321	3.9E-02	1.7E-02	0.174	0.009	0.339	6.1E-01	2.5E-02	0.464	-1.310	2.238	4.8E-01	3.6E-01	0.023	-0.040	0.085
	B*42:01	27	2	0				●	●	6.0E-01	1.5E-01	-0.106	-0.501	0.289	4.1E-01	1.1E-01	45.178	-61.063	151.419	4.6E-01	1.3E-01	0.127	-0.214	0.469	1.4E-02	2.0E-03	4.631	0.944	8.319	1.0E-03	3.8E-03	0.224	0.092	0.356
	B*51:07	6	3	1		●	●	●		1.4E-01	6.0E-02	-0.608	-1.419	0.203	2.4E-02	1.7E-02	246.924	33.116	460.731	3.8E-02	1.7E-02	0.729	0.042	1.416	2.2E-02	2.4E-03	8.680	1.244	16.116	5.5E-02	9.3E-02	0.255	-0.006	0.516

HLA-HIV PROTE	B*57:01	30	5	2	●	●	●	●	●	**2.5E-02**	**3.0E-02**	-0.449	-0.841	-0.057	**2.1E-02**	**1.7E-02**	121.753	18.301	225.206	**7.0E-03**	**9.0E-03**	0.456	0.123	0.788	**3.0E-03**	**6.0E-04**	5.480	1.888	9.071	**3.0E-02**	**6.5E-02**	0.139	0.013	0.265
	B*57:02	5	2	0				●	●	2.8E-01	**8.7E-02**	-0.513	-1.442	0.416	2.2E-01	**7.0E-02**	151.914	-90.174	394.001	1.8E-01	**5.9E-02**	0.543	-0.244	1.330	**1.0E-03**	**5.0E-04**	13.440	5.291	21.588	**1.0E-05**	**5.1E-05**	0.984	0.668	1.301
	B*57:03	21	0	0						8.6E-02	**4.8E-02**	-0.466	-0.998	0.066	2.3E-01	**7.0E-02**	85.914	-53.950	225.778	7.5E-02	**3.0E-02**	0.408	-0.042	0.857	1.6E-01	**8.5E-03**	2.996	-1.210	7.203	1.4E-01	**1.7E-01**	0.111	-0.036	0.259
	C*12:03	96	3	1		●	●	●		2.1E-01	**7.0E-02**	-0.136	-0.351	0.078	**4.0E-03**	**5.1E-03**	83.953	27.429	140.478	**1.6E-02**	**1.2E-02**	0.224	0.042	0.407	**3.7E-02**	**3.3E-03**	2.091	0.123	4.060	1.5E-01	**1.7E-01**	0.051	-0.018	0.120
	C*17:01	60	0	0						5.1E-01	**1.4E-01**	-0.089	-0.355	0.178	1.4E-01	**5.3E-02**	53.361	-17.477	124.199	2.2E-01	**6.8E-02**	0.141	-0.086	0.369	1.6E-01	**8.5E-03**	1.763	-0.699	4.224	9.6E-01	5.9E-01	-0.003	-0.105	0.099
	C*18:01	29	5	2	●	●	●	●	●	**4.1E-02**	**3.5E-02**	-0.450	-0.881	-0.019	**4.0E-03**	**5.1E-03**	146.358	48.008	244.709	**1.8E-02**	**1.2E-02**	0.441	0.076	0.806	**7.0E-03**	**1.2E-03**	4.725	1.311	8.139	**1.0E-05**	**5.1E-05**	0.233	0.112	0.354
HLA-HIV RISK associations	A*02:01	538	0	0						4.0E-01	**1.1E-01**	-0.048	-0.160	0.064	5.9E-02	**2.9E-02**	-28.389	-57.858	1.081	5.2E-01	**1.4E-01**	-0.032	-0.128	0.065	5.8E-01	**2.5E-02**	-0.289	-1.302	0.724	3.2E-01	2.7E-01	-0.018	-0.054	0.018
	A*02:06	130	-1	0					●	1.6E-01	**6.4E-02**	-0.135	-0.325	0.054	1.5E-01	**5.5E-02**	-36.884	-87.592	13.824	9.0E-01	2.3E-01	-0.010	-0.173	0.152	7.7E-02	**6.4E-03**	-1.583	-3.336	0.170	**4.6E-02**	**8.7E-02**	-0.063	-0.124	-0.001
	A*24:02	466	-2	-1		●	●			8.9E-02	**4.8E-02**	0.100	-0.015	0.215	**3.1E-02**	**2.0E-02**	-33.484	-63.970	-2.998	**7.0E-03**	**9.0E-03**	-0.136	-0.234	-0.038	2.9E-01	**1.4E-02**	-0.570	-1.624	0.483	5.5E-01	4.0E-01	-0.011	-0.049	0.026
	A*36:01	16	-3	-1	●		●	●		**3.4E-02**	**3.4E-02**	0.558	0.041	1.075	2.9E-01	**8.5E-02**	-72.959	-208.659	62.741	**3.5E-02**	**1.7E-02**	-0.469	-0.905	-0.034	**3.0E-02**	**3.0E-03**	-5.065	-9.632	-0.498	1.2E-01	**1.6E-01**	-0.132	-0.297	0.033
	A*68:01	148	-5	-2	●	●	●	●	●	**1.5E-02**	**2.5E-02**	0.217	0.043	0.391	**1.0E-05**	**3.2E-05**	-83.968	-130.048	-37.889	**1.0E-05**	**6.4E-05**	-0.270	-0.418	-0.122	**2.0E-03**	**5.0E-04**	-2.513	-4.104	-0.922	**2.5E-02**	**6.3E-02**	-0.064	-0.121	-0.008
	A*68:03	195	-3	0			●	●	●	1.1E-01	**4.9E-02**	0.131	-0.028	0.291	1.3E-01	**5.3E-02**	-33.621	-77.569	10.327	**1.4E-02**	**1.2E-02**	-0.169	-0.304	-0.034	**2.0E-03**	**5.0E-04**	-2.261	-3.716	-0.805	**6.0E-03**	**1.8E-02**	-0.071	-0.123	-0.020
	B*35:02	20	-2	-1	●		●			**1.7E-02**	**2.5E-02**	0.577	0.101	1.052	7.3E-02	**3.3E-02**	-113.149	-236.853	10.555	**7.0E-03**	**9.0E-03**	-0.547	-0.944	-0.150	1.4E-01	**8.5E-03**	-3.220	-7.524	1.085	2.3E-01	2.4E-01	-0.093	-0.244	0.058
	B*35:12	114	-1	0			●			8.0E-02	**4.8E-02**	0.195	-0.023	0.413	2.2E-01	**7.0E-02**	-33.825	-87.771	20.122	**1.1E-02**	**1.2E-02**	-0.226	-0.399	-0.053	8.5E-01	**3.4E-02**	-0.186	-2.062	1.690	7.7E-01	5.1E-01	0.010	-0.056	0.076
	B*39:05	114	-1	-1		●				1.9E-01	**6.8E-02**	0.138	-0.068	0.345	**2.0E-02**	**1.7E-02**	-67.453	-124.337	-10.570	8.4E-02	**3.2E-02**	-0.161	-0.344	0.022	1.5E-01	**8.5E-03**	-1.464	-3.439	0.512	9.7E-01	5.9E-01	-0.001	-0.071	0.068
	C*03:03	75	0	0						7.3E-01	2.1E-01	0.043	-0.196	0.282	5.6E-02	**2.9E-02**	-61.404	-124.267	1.459	2.2E-01	**6.8E-02**	-0.126	-0.328	0.077	1.2E-01	**8.5E-03**	-1.737	-3.923	0.450	2.4E-01	2.4E-01	-0.046	-0.123	0.030
	C*04:01	569	-2	-1	●		●			**6.0E-03**	**1.8E-02**	0.163	0.046	0.279	1.3E-01	**5.3E-02**	-22.035	-50.830	6.760	**2.5E-02**	**1.3E-02**	-0.113	-0.212	-0.014	5.6E-01	**2.5E-02**	-0.295	-1.295	0.705	7.7E-01	5.1E-01	-0.005	-0.041	0.030
	C*07:02	505	0	0						1.9E-01	**6.8E-02**	0.078	-0.040	0.195	4.1E-01	**1.1E-01**	-13.706	-46.481	19.069	1.5E-01	**5.5E-02**	-0.072	-0.172	0.027	2.1E-01	**1.0E-02**	-0.689	-1.766	0.389	3.0E-01	2.7E-01	-0.020	-0.058	0.018

Supplementary Table S10. Summary of univariable and multivariable analysis of PROTECTIVE and RISK HLA-HIV associations using 5 HIV clinical parameters in the pooled MEX/CAM and individual cohorts. ● denotes HLA-HIV clinical parameter associations that were significant in univariable (Mann-Whitney) analysis as well as multivariable linear regression (Generalized Linear Model, GLM) analyses correcting for gender, age, country/region of residence, and the effect of the most significant HLA associations for that parameter (see Methods and Supplementary Table S9, model 1); and every HLA in LD with the allele associated with an HIV clinical parameter (model 2). Multiple comparisons were addressed using q-values, with p-values <0.05 and q-values <0.2 considered as significant. PDA, previously described association in literature (see last column for references). α, HLA-HIV association found with a contrary effect in our cohorts. New association, HLA-HIV association found in the present study and to our knowledge not previously reported.

Pooled MEX/CAM cohort (n=3213)

HIV outcome	PDA	New association	HLA allele	UNIVARIABLE (Mann-Whitney U test [p<0.05, q<0.2])							MULTIVARIABLE model 1 (Linear regression [GLM] with gender, geographic origin, age, and HLA alleles with p<0.001 for every clinical parameter as covariables [p<0.05, q<0.2])						MULTIVARIABLE model 2 (Linear regression [GLM] with HLA alleles in linkage disequilibrium as covariables [p<0.05, q<0.2])							
				N	HLA-HIV score	pVL	CD4	Z-score	%CD4	CD4/CD8	HLA-HIV score	pVL	CD4	Z-score	%CD4	CD4/CD8	Linkage disequilibrium	HLA-HIV score	pVL	CD4	Z-score	%CD4	CD4/CD8	Observations
PROTECTIVE HLA-HIV ASSOCIATIONS	•		A*02:05	71	4		•	•	•	•	3		•	•	•		B*41:01, C*07:01, B*50:01, B*58:01	4	•	•	•	•		Remained significant after accounting for the presence of HLA alleles in LD.
		•	A*03:01	299	4		•	•	•	•	3		•	•	•		B*07:02, B*39:05	3		•	•	•		Remained significant after accounting for the presence of HLA alleles in LD.
	•		A*11:01	202	4		•	•	•	•	1		•				B*39:05	3		•	•	•		Remained significant after accounting for the presence of HLA alleles in LD.
	•		A*25:01	44	3		•		•	•	3		•		•	•	C*12:03, B*18:01	1					•	Remained significant after accounting for the presence of HLA alleles in LD. C*12:03 also had a significant effect in the model.
	•		A*26:01	143	2		•	•			1		•				B*38:01, C*12:03	2		•	•			Remained significant after accounting for the presence of HLA alleles in LD. C*12:03 also had a significant effect in the model.
	•		A*30:02	171	2	•		•			1	•					C*05:01, B*18:01, C*07:02	2	•		•			Remained significant after accounting for the presence of HLA alleles in LD.
	• α		B*07:02	301	1		•				2		•		•		C*07:02, B*07:02, C*15:05, C*04:01	3		•	•	•		Remained significant after accounting for the presence of HLA alleles in LD.
	•		B*14:02	189	5	•	•	•	•	•	3	•			•	•	C*08:02, B*14:02, C*07:02, B*14:02	0						Cannot define association due to strong LD with C*08:02 (166/184 of B*14:02+ are also C*08:02+, p=7.96E-195).
		•	B*15:39	8	2	•		•			4	•	•	•		•	C*03:03	5	•	•	•	•	•	Remained significant after accounting for the presence of HLA alleles in LD.
	•		B*27:05	65	5	•	•	•	•	•	5	•	•	•	•	•	C*02:02, C*07:02, C*01:02	5	•	•	•	•	•	Remained significant after accounting for the presence of HLA alleles in LD.
		•	B*39:02	98	2				•	•	3	•			•	•	C*07:02	2				•	•	Remained significant after accounting for the presence of HLA alleles in LD.
	•		B*40:05	18	2		•		•		1		•				C*03:04	2		•		•		Remained significant after accounting for the presence of HLA alleles in LD.
	•		B*40:06	8	2		•	•			2		•	•			---	NA						No LD found.
	•		B*42:01	42	4		•	•	•	•	2				•	•	C*17:01, A*30:01	3			•	•	•	Remained significant after accounting for the presence of HLA alleles in LD.
	•		B*44:03	221	1					•	0						C*16:01, A*29:02, C*07:02	1					•	Remained significant after accounting for the presence of HLA alleles in LD.
	•		B*51:07	NA	0						NA						NA	NA						---
	•		B*57:01	61	5	•	•	•	•	•	5	•	•	•	•	•	A*01:01, C*06:02, C*07:01	5	•	•	•	•	•	Remained significant after accounting for the presence of HLA alleles in LD.
	•		B*57:02	7	2				•	•	2				•	•	C*18:01	2				•	•	Remained significant after accounting for the presence of HLA alleles in LD.
	•		B*57:03	38	5	•	•	•	•	•	4	•	•	•	•		C*18:01	3	•	•	•			Remained significant after accounting for the presence of HLA alleles in LD.
	•		B*58:01	85	2	•		•			0						C*03:02, C*07:01, A*33:03, A*02:05	0						LD with A*02:05 could explain significant effect.
	•		B*81:01	16	2	•		•			-1					•	C*18:01	1					•	Remained significant after accounting for the presence of HLA alleles in LD.
	•		C*02:02	162	5	•	•	•	•	•	0						B*27:05, B*15:03	0						LD with B*27:05 could explain significant effect.
	•		C*03:02	71	1	•					0						A*33:03, B*58:01	0						Cannot define association due to strong LD with with B*58:01 (33/69 of C*03:02+ are also B*58:01+, p=1.83E-36)
	•		C*03:03	NA	0						NA						NA	NA						---
	•		C*03:05	222	1	•					0						B*40:02, A*24:02	1	•					Remained significant after accounting for the presence of HLA alleles in LD.
	•		C*08:01	160	1				•		1					•	B*48:01, B*51:02, A*02:06	0						Cannot define association due to strong LD with B*48:01 and B*51:02 (PDA) (116/160 of C*08:01+ are also B*48:01+ or B*5102+; p=2.03E 133, p=8.78E-21, respectively).
	•		C*08:02	246	5	•	•	•	•	•	4	•	•		•	•	B*14:02, A*33:01, B*14:01, B*35:01	0						Cannot define association due to strong LD with B*14:02 (PDA) (166/243 of C*08:02+ are also B*14:02+; p=7.96E-195).
	•		C*12:02	67	1	•					0						B*52:01	0						Cannot define association due to strong LD with B*52:01 (54/67 of C*12:02+ are also B*52:01+; p=2.30E-64).
	•		C*12:03	189	3		•	•	•		3		•	•	•		B*38:01, B*18:01, A*26:01, A*25:01, B*39:01	3		•	•	•		Remained significant after accounting for the presence of HLA alleles in LD.
	•		C*14:02	55	3	•	•	•			4	•	•	•	•		B*51:01, B*51:07, B*15:16	2		•	•			Remained significant after accounting for the presence of HLA alleles in LD.
	•		C*14:03	6	1	•					2	•		•			---	NA						No LD found
	•		C*17:01	106	3		•		•	•	0						B*42:01, A*30:01, B*41:02, B*41:01, B*42:02	0						LD with B*42:01 could explain significant effect.
	•		C*18:01	37	5	•	•	•	•	•	4		•	•	•	•	B*57:03, B*81:01, B*57:02	1					•	Remained significant after accounting for the presence of HLA alleles in LD. B*57:03 also had a significant effect in the model.
		•	A*01:02	8	-1	•					-2	•		•			B*49:01	-1	•					Remained significant after accounting for the presence of HLA alleles in LD.
	•		A*02:01	1278	-2		•			•	0						B*35:12, B*15:15, C*03:03, B*44:02, B*35:17	0						LD with B*35:12 could explain significant effect.
		•	A*02:06	441	-1		•				1	•					C*07:02, B*39:05, B*39:08, C*08:01	-1		•				Remained significant after accounting for the presence of HLA alleles in LD. C*07:02 (LD with B*39 alleles) and B*39:05 also had a significant effect in the model.
	•		A*23:01	NA	0						NA						NA	NA						---
	•		A*24:02	966	-2	•		•			0						C*03:05, B*39:06, B*40:02	-2	•		•			Remained significant after accounting for the presence of HLA alleles in LD.
		•	A*24:03	NA	0						NA						NA	NA						---
	•		A*36:01	NA	0												NA	NA						---

MEX cohort (n=1679)

HLA allele	UNIVARIABLE (Mann-Whitney U test [p<0.05, q<0.2])							MULTIVARIABLE model 1 (Linear regression [GLM] with gender, geographic origin, age, and HLA alleles with p<0.001 for every clinical parameter as covariables [p<0.05, q<0.2])						MULTIVARIABLE model 2 (Linear regression [GLM] with HLA alleles in linkage disequilibrium as covariables [p<0.05, q<0.2])							
	N	HLA-HIV score	pVL	CD4	Z-score	%CD4	CD4/CD8	HLA-HIV score	pVL	CD4	Z-score	%CD4	CD4/CD8	Linkage disequilibrium	HLA-HIV score	pVL	CD4	Z-score	%CD4	CD4/CD8	Observations
A*02:05	NA	0						NA						---	NA						__
A*03:01	137	3		•	•	•		3		•	•	•		B*07:02	3		•	•	•		Remained significant after accounting for the presence of HLA alleles in LD.
A*11:01	NA	0						NA						---	NA						---
A*25:01	NA	0						NA						---	NA						---
A*26:01	62	2		•	•			2		•	•			B*38:01, C*12:03	2		•	•			Remained significant after accounting for the presence of HLA alleles in LD.
A*30:02	NA	0						NA						---	NA						---
B*07:02	NA	0						NA						---	NA						---
B*14:02	101	5	•	•	•	•	•	1					•	C*08:02, A*33:01, A*68:02	1			•			Remained significant after accounting for the presence of HLA alleles in LD.
B*15:39	6	5	•	•	•	•	•	5	•	•	•	•	•	---	5	•	•	•	•	•	Remained significant after accounting for the presence of HLA alleles in LD.
B*27:05	46	5	•	•	•	•	•	5	•	•	•	•	•	C*02:02	5	•	•	•	•	•	Remained significant after accounting for the presence of HLA alleles in LD.
B*39:02	68	2	•				•	3	•			•	•	C*07:02	4	•		•	•	•	Remained significant after accounting for the presence of HLA alleles in LD.
B*40:05	18	1		•				1		•				C*03:04	2		•		•		Remained significant after accounting for the presence of HLA alleles in LD.
B*40:06	NA	0						NA						---	NA						---
B*42:01	NA	0						NA						---	NA						---
B*44:03	NA	0						NA						---	NA						---
B*51:07	NA	0						NA						---	NA						---
B*57:01	31	4	•		•	•	•	5	•	•	•	•	•	A*01:01, C*06:02, C*07:01	5	•	•	•	•	•	Remained significant after accounting for the presence of HLA alleles in LD. C*06:02 also had a significant effect in the model.
B*57:02	NA	0						NA						---	NA						---
B*57:03	17	2	•		•			5	•	•	•	•	•	C*18:01	4	•	•	•		•	Remained significant after accounting for the presence of HLA alleles in LD.
B*58:01	NA	0						NA						---	NA						---
B*81:01	NA	0						NA						---	NA						---
C*02:02	74	4		•	•	•	•	0						B*15:03, B*27:05	0						LD with B*27:05 could explain significant effect.
C*03:02	NA	0						NA						---	NA						---
C*03:03	121	2	•				•	4	•		•	•	•	B*15:01, B*52:01, B*55:01	5	•	•	•	•	•	Remained significant after accounting for the presence of HLA alleles in LD.
C*03:05	NA	0						NA						---	NA						---
C*08:01	143	1	•					1					•	B*48:01, B*51:02	3	•		•		•	Cannot define association due to strong LD with B*48:01 and B*51:02 (PDA) (113/143 of C*08:01+ are also B*48:01+ or B*5102+; p=1.42E 107, p=4.87E-15, respectively).
C*08:02	132	5	•	•	•	•	•	2		•			•	B*14:02, A*33:01, B*14:01, A*68:02	0						Cannot define association due to strong LD with B*14:02 (PDA) (89/134 of C*08:02+ are also B*14:02+; p=2.94E-101).
C*12:02	NA	0						NA						---	NA						---
C*12:03	NA	0						NA						---	NA						---
C*14:02	25	5	•	•	•	•	•	5	•	•	•	•	•	B*51:01	5	•	•	•	•	•	Remained significant after accounting for the presence of HLA alleles in LD.
C*14:03	NA	0						NA						---	NA						---
C*17:01	NA	0						NA						---	NA						---
C*18:01	NA	0						NA						---	NA						---
A*01:02	NA	0						NA						---	NA						---
A*02:01	NA	0						NA						---	NA						---
A*02:06	NA	0						NA						---	NA						---
A*23:01	59	-1	•					-1	•					B*15:03	-1	•					Remained significant after accounting for the presence of HLA alleles in LD.
A*24:02	NA	0						NA						---	NA						---
A*24:03	6	-1	•					-1	•					---	NA						No LD found.
A*36:01	NA	0						NA						---	NA						---

CAM cohort (n=1534)

HLA allele	UNIVARIABLE (Mann-Whitney U test [p<0.05, q<0.2])							MULTIVARIABLE model 1 (Linear regression [GLM] with gender, geographic origin, age, and HLA alleles with p<0.001 for every clinical parameter as covariables [p<0.05, q<0.2])						MULTIVARIABLE model 2 (Linear regression [GLM] with HLA alleles in linkage disequilibrium as covariables [p<0.05, q<0.2])								Reference of previously described associations
	N	HLA-HIV score	pVL	CD4	Z-score	%CD4	CD4/CD8	HLA-HIV score	pVL	CD4	Z-score	%CD4	CD4/CD8	Linkage disequilibrium	HLA-HIV score	pVL	CD4	Z-score	%CD4	CD4/CD8	Observations	
A*02:05	38	2		•	•			3		•	•	•		B*50:01	3		•	•	•		Remained significant after accounting for the presence of HLA alleles in LD.	Kipiela 2004 (LD B5801), Koehler 2010, Leslie 2010
A*03:01	162	1		•				1		•				B*07:02	2		•	•			Remained significant after accounting for the presence of HLA alleles in LD.	
A*11:01	94	4		•	•	•	•	2		•	•			---	NA						---	O'Brien 2001 (A11)
A*25:01	20	3		•		•	•	4		•	•	•	•	B*18:01, C*12:03	3		•		•	•	Remained significant after accounting for the presence of HLA alleles in LD. C*12:03 also had a significant effect in the model.	Kaslow 1996 (A25), Fellay 2009
A*26:01	NA	0						NA						---	NA						---	Kaslow 1996 (A26), O'Brien 2001 (A26)
A*30:02	116	2	•		•			2	•		•			B*18:01, C*05:01	2	•		•			Remained significant after accounting for the presence of HLA alleles in LD.	Tang 2002 (A30/C03), Koehler 2010(LD B5703), Tang 2010 (A30+C03)
B*07:02	NA	0						NA						---	NA						---	Fellay 2009 (opposite effect)
B*14:02	NA	0						NA						---	NA						---	Lazaryan 2011
B*15:39	NA	0						NA						---	NA						---	
B*27:05	NA	0						NA						---	NA						---	O'Brien 2001 (B27), Gao 2001 (B27), Fellay 2009, Kaslow 1996 (B27)
B*39:02	NA	0						NA						---	NA						---	
B*40:05	NA	0						NA						---	NA						---	Matthews 2012 (B40)
B*40:06	NA	0						NA						---	NA						---	Naruto 2012
B*42:01	27	2				•	•	2				•	•	C*17:01, A*30:01	2				•	•	Remained significant after accounting for the presence of HLA alleles in LD. C*17:01 also had a significant effect in the model.	Matthews 2012, Leslie 2010, Carlson 2012
B*44:03	NA	0						NA						---	NA						---	Naruto 2012, Leslie 2010, Carlson 2012
B*51:07	6	1					•	3		•	•	•		C*14:02	4		•	•	•	•	Remained significant after accounting for the presence of HLA alleles in LD.	O'Brien 2001 (B51)
B*57:01	30	4		•	•	•	•	5	•	•	•	•	•	C*07:01, C*06:02	4	•		•	•	•	Remained significant after accounting for the presence of HLA alleles in LD.	Kaslow 1996 (B57), Migueles 2000, Gao 2001 (B57), Tang 2002 (B57), O'Brien 2001 (B57), Kipiela 2004, Lazaryan 2011, Matthews 2012, Tang 2010, Fellay 2009
B*57:02	5	5	•	•	•	•	•	2				•	•	---	NA						No LD found.	O'Brien 2001 (B57), Leslie 2010, Kloverpris 2012
B*57:03	21	3	•	•	•			0						C*18:01	2	•		•			Remained significant after accounting for the presence of HLA alleles in LD. C*18:01 (LD B*81:01) also had a significant effect in the model.	O'Brien 2001 (B57), Leslie 2010, Costello, 1999, Lazaryan 2011, Lazaryan 2006, Matthews 2011, Carlson 2012
B*58:01	NA	0						NA						---	NA						---	Leslie 2010, Kipiela 2004, Naruto 2012, Koehler 2010 (ce, ACDclade), Matthews 2012, Carlson 2012
B*81:01	NA	0						NA						---	NA						---	Kipiela 2004, Matthews 2012, Leslie 2010, Carlson 2012
C*02:02	NA	0						NA						---	NA						---	O'Brien 2001 (C02), Fellay 2009
C*03:02	NA	0						NA						---	NA						---	Leslie 2010
C*03:03	NA	0						NA						---	NA						---	Naruto 2012 (ce), Tang 2010 (A30+C03)
C*03:05	NA	0						NA						---	NA						---	Tang 2010 (A30+C03)
C*08:01	NA	0						NA						---	NA						---	Matthews 2012 (C08)
C*08:02	NA	0						NA						---	NA						---	Matthews 2012 (C08)
C*12:02	NA	0						NA						---	NA						---	Naruto 2012, Matthews 2012 (C12)
C*12:03	96	2		•	•			3		•	•	•		B*38:01, B*18:01, A*26:01, B*39:01, A*25:01	2		•		•		Remained significant after accounting for the presence of HLA alleles in LD. A*25:01 also had a significant effect in the model.	Matthews 2012 (C12), Leslie 2010, Carlson 2012
C*14:02	NA	0						NA						---	NA						---	O'Brien 2001 (C14), Carlson 2012
C*14:03	NA	0						NA						---	NA						---	O'Brien 2001 (C14), Naruto 2012
C*17:01	60	3		•		•	•	0						B*42:01, A*30:01, B*41:02, B*42:02	1		•				Remained significant after accounting for the presence of HLA alleles in LD. B*42:01 also had a significant effect in the model.	Matthews 2012 (C17), Carlson 2012 (ce)
C*18:01	29	5	•	•	•	•	•	5	•	•	•	•	•	B*81:01, B*57:03	4		•	•	•	•	Remained significant after accounting for the presence of HLA alleles in LD. B*81:01 and B*57:03 also had significant effects in the model.	Tang 2002 (C18), Koehler 2010(LD B5703), Matthews 2012 (C18), Leslie 2010
A*01:02	NA	0						NA						---	NA						---	
A*02:01	538	-1		•				0						B*35:12	0						LD with B*35:12 could explain significant effect.	Naruto 2012
A*02:06	130	-1		•				-1					•	B*39:08, C*07:02	-2		•			•	Remained significant after accounting for the presence of HLA alleles in LD. C*07:02 (LD with B*39 alleles) also had a significant effect in the model.	
A*23:01	NA	0						NA						---	NA						---	Kaslow 1996 (A23), McDonald 2000
A*24:02	466	-2	•		•			-2		•	•			C*03:05, B*40:02, C*01:02	-2	•		•			Remained significant after accounting for the presence of HLA alleles in LD.	Kaslow 1996 (A24), McDonald 2000 (C-clade), Fellay 2009.
A*24:03	NA	0						NA						---	NA						---	
A*36:01	16	-1	•					-3	•		•	•		B*53:01	-1	•					Remained significant after accounting for the presence of HLA alleles in LD.	Tang 2010, Carlson 2012

DISADVANTAGEOUS HLA-HIV ASSOCIATIONS

•		A*68:01	382	-5	•	•	•	•	•	-5	•	•	•	•	•	C*03:04	-4	•	•	•	•		Remained significant after accounting for the presence of HLA alleles in LD.	NA	0						NA						---	NA						---	148	-5	•	•	•	•	•	-5	•	•	•	•	•	---	0						No LD found.	Matthews 2012, Leslie 2010
	•	A*68:03	370	-5	•	•	•	•	•	-2				•	•	B*39:05, C*07:02, B*35:43	-3		•		•	•	Remained significant after accounting for the presence of HLA alleles in LD. B*39:05 and C*07:02 (LD with B*39 alleles) also had a significant effect in the model.	175	-3		•		•	•	-3		•	•		•	B*39:05, C*07:02	0						Cannot define association due to strong LD with B*39:05 (102/175 of A*68:03+ are also B*39:05+; p=5.23E-51).	195	-5	•	•	•	•	•	-3			•	•	•	B*39:05, C*07:02, B*35:43	-4	•		•	•	•	Remained significant after accounting for the presence of HLA alleles in LD. B*39:05 and C*07:02 (LD with B*39 alleles) also had a significant effect in the model.	
	•	A*68:05	60	-2				•	•	-1				•		---	NA						No LD found	NA	0						NA						---	NA						---	NA	0						NA						---	NA						---	
• α		B*13:02	65	-1					•	0						C*06:02, A*30:01	-1					•	Remained significant after accounting for the presence of HLA alleles in LD.	NA	0						NA						---	NA						---	NA	0						NA						---	NA						---	Tang 2002 (B13), Tang 2010, Leslie 2010, Fellay 2009 (opposite effect)
	•	B*15:30	20	-3		•		•	•	-1				•		C*01:02	-1				•		Remained significant after accounting for the presence of HLA alleles in LD.	17	-2				•	•	-1				•		C*01:02	-1				•		Remained significant after accounting for the presence of HLA alleles in LD.	NA	0						NA						---	NA						---	
•		B*35:01	645	-4	•	•	•	•		-1				•		C*04:01, C*07:01, C*08:02, C*01:02	0						Cannot define association due to strong LD with C*04:01 (420/583 of B*35:01+ are also C*04:01+; p=1.93E-96).	NA	0						NA						---	NA						---	NA	0						NA						---	NA						---	Klein 1994 (B35), Kaslow 1996 (B35), Carrington 1999 (B35-C04), O'Brien 2001 (B35), Naruto 2012, Carlson 2012
•		B*35:02	51	-2	•		•			-2	•		•			C*04:01	-3	•		•		•	Remained significant after accounting for the presence of HLA alleles in LD.	NA	0						NA						---	NA						---	20	-2	•		•			-2	•		•			C*04:01	-2	•		•			Remained significant after accounting for the presence of HLA alleles in LD. C*04:01 (LD with B*35 alleles) also had a significant effect in the model.	Klein 1994 (B35), Kaslow 1996 (B35), Carrington 1999 (B35-C04), O'Brien 2001 (B35), Gao 2001, Fellay 2009
	•	B*35:12	273	-5	•	•	•	•	•	-1		•				C*04:01, A*02:01	-2		•	•			Remained significant after accounting for the presence of HLA alleles in LD.	159	-3		•		•	•	-1		•				A*02:01, C*04:01	0						Cannot define association due to strong LD with A*02:01 and C*04:01 (94/151 of B*35:12+ are also A*02:01+ and C*04:01+; p=1.37E-10, p=9.19E-60, respectively).	114	-3	•	•	•			-1			•			C*04:01, A*02:01	-2	•		•			Remained significant after accounting for the presence of HLA alleles in LD.	
	•	B*35:14	38	-1			•			0						C*04:01	0						LD with C*04:01 (LD with B*35 alleles) could explain significant effect. Potential lack of statistical power due to low N.	NA	0						NA						---	NA						---	NA	0						NA						---	NA						---	
•		B*37:01	NA	0						NA						NA	NA						---	11	-1	•					-1	•					C*06:02	0						Cannot define association due to strong LD with C*06:02 (11/11 of B*37:01+ are also C*06:02+; p=5.42E-13). Potentially lack of statistical power due to low N.	NA	0						NA						---	NA						---	Kaslow 1996 (B37), Naruto 2012
	•	B*39:01	127	-1		•				0						C*07:02, C*12:03	0						LD with C*07:02 (LD with other B*39 alleles) could explain significant effect.	NA	0						NA						---	NA						---	NA	0						NA						---	NA						---	
	•	B*39:05	360	-5	•	•	•	•	•	-2		•		•		C*07:02, A*68:03, A*02:06, A*23:01, A*03:01, A*11:01	0						Cannot define association due to strong LD with C*07:02 (317/337 of B*39:05+ are also C*07:02+; p=3.82E-144)	246	-4		•	•	•	•	-3		•	•	•		C*07:02, A*68:03, A*02:06	0						Cannot define association due to strong LD with C*07:02 (223/235 of B*39:05+ are also C*07:02+; p=4.62E-97).	114	-4		•	•	•	•	-1		•				A*68:03, C*07:02	0						Remained significant after accounting for the presence of HLA alleles in LD. A*68:03 (LD C*07:02 and B*35:43) also had a significant effect in the model.	
	•	B*39:06	153	-2	•		•			0						C*07:02, A*24:02	0						LD with C*07:02 (LD with other B*39 alleles) could explain significant effect.	NA	0						NA						---	NA						---	NA	0						NA						---	NA						---	
	•	B*40:01	46	-2				•	•	0						C*03:04	0						Cannot define association due to strong LD with C*03:04 (35/46 of B*40:01+ are also C*03:04+; p=2.54E-23), also potentially lack of statistical power due to low N.	NA	0						NA						---	NA						---	NA	0						NA						---	NA						---	
•		C*03:03	NA	0						NA						NA	NA						LD with B*15:39 could explain significant effect.	NA	0						NA						---	NA						---	75	-1		•				0						B*55:01, B*15:01, B*52:01	-1		•				Remained significant after accounting for the presence of HLA alleles in LD.	Naruto 2012
	•	C*03:04	427	-1	•					-1				•		B*40:02, B*40:01, B*15:10, B*40:08, B*40:05, A*68:01, B*40:11	-1	•					Remained significant after accounting for the presence of HLA alleles in LD. B*15:10, B*40:05, A*68:01 also had a significant effect in the model.	NA	0						NA						---	NA						---	NA	0						NA						---	NA						---	
•		C*04:01	1237	-3	•	•	•			-2	•		•			B*35:12, B*35:01, B*35:17, B*53:01, B*35:02, B*35:14, B*35:20, B*40:02, B*35:16, B*49:01, B*35:08, B*07:02	0						LD with B*35:12, B*35:01, and B*35:02 could explain significant effect.	668	-2		•	•			0						B*35:12, B*35:01, B*35:17, B*35:02, B*35:14, B*53:01, B*35:16, B*14:01	0						LD with B*35:12 could explain significant effect.	569	-2	•		•			-2	•		•			B*35:12, B*35:01, B*53:01, B*35:17, B*35:20, B*35:02	0						LD with B*35:12, and B*35:02 could explain significant effect.	Carrington 1999 (B35-C04), Naruto 2012, Lazaryan 2011 (LDB35). O'Brien 2001 (B35), Fellay 2009
•		C*06:02	NA	0						NA						NA	NA						---	140	-2	•		•			-1	•					B*13:02, A*30:01, B*37:01, B*57:01, B*45:01, B*50:01, B*58:02, A*01:01	-4		•	•	•	•	Remained significant after accounting for the presence of HLA alleles in LD. B*57:01 also had a significant effect in the model.	NA	0						NA						---	NA						---	Matthews 2012 (C06)
•		C*07:02	1171	-5	•	•	•	•	•	-1	•					B*39:05, B*07:02, B*39:06, A*68:03, B*39:08, B*39:02, B*39:01, B*39:11, A*02:06, B*44:03, B*15:01, B*40:02, A*01:01, B*14:02, B*27:05, A*30:02	-4	•	•	•	•		Remained significant after accounting for the presence of HLA alleles in LD. B*07:02, B*39:02, B*39:08, B*14:02, and B*27:05 also had a significant effect in the model.	666	-4		•	•	•	•	-3	•	•	•			B*39:05, B*39:06, B*07:02, A*68:03, B*39:01, B*39:02, B*39:08, B*15:01, B*44:03	-1				•		Remained significant after accounting for the presence of HLA alleles in LD. B*39:02, and B*07:02 also had a significant effect in the model.	505	-3		•	•		•	0						B*07:02, B*39:05, B*39:08, B*39:11, A*68:03, B*39:06, A*02:06	-3	•	•	•			Remained significant after accounting for the presence of HLA alleles in LD. A*68:03, and A*02:06 also had a sigficant effect in the model.	Koehler 2010. Fellay 2009.
•		C*16:01	NA	0						NA						NA	NA						---	98	-1			•			-1				•		B*44:03, A*29:02, B*45:01	-4		•	•	•	•	Remained significant after accounting for the presence of HLA alleles in LD.	NA	0						NA						---	NA						---	Matthews 2012 (C16)
